# Cytogenetic and Sequence Analyses of Mitochondrial DNA Insertions in Nuclear Chromosomes of Maize

**DOI:** 10.1534/g3.115.020677

**Published:** 2015-09-01

**Authors:** Ashley N. Lough, Kaitlyn M. Faries, Dal-Hoe Koo, Abid Hussain, Leah M. Roark, Tiffany L. Langewisch, Teresa Backes, Karl A. G. Kremling, Jiming Jiang, James A. Birchler, Kathleen J. Newton

**Affiliations:** * Division of Biological Sciences, University of Missouri, Columbia, Missouri 65211; † Department of Horticulture, University of Wisconsin, Madison, Wisconsin 53706

**Keywords:** NUMT, FISH, variation, diversity

## Abstract

The transfer of mitochondrial DNA (mtDNA) into nuclear genomes is a regularly occurring process that has been observed in many species. Few studies, however, have focused on the variation of nuclear-mtDNA sequences (NUMTs) within a species. This study examined mtDNA insertions within chromosomes of a diverse set of *Zea mays* ssp. *mays* (maize) inbred lines by the use of fluorescence *in situ* hybridization. A relatively large NUMT on the long arm of chromosome 9 (9L) was identified at approximately the same position in four inbred lines (B73, M825, HP301, and Oh7B). Further examination of the similarly positioned 9L NUMT in two lines, B73 and M825, indicated that the large size of these sites is due to the presence of a majority of the mitochondrial genome; however, only portions of this NUMT (∼252 kb total) were found in the publically available B73 nuclear sequence for chromosome 9. Fiber-fluorescence *in situ* hybridization analysis estimated the size of the B73 9L NUMT to be ∼1.8 Mb and revealed that the NUMT is methylated. Two regions of mtDNA (2.4 kb and 3.3 kb) within the 9L NUMT are not present in the B73 mitochondrial NB genome; however, these 2.4-kb and 3.3-kb segments are present in other *Zea* mitochondrial genomes, including that of *Zea mays* ssp. *parviglumis*, a progenitor of domesticated maize.

The endosymbiotic theory posits that mitochondria originated from an independent organism related to modern α-proteobacteria (reviewed by Timmis *et al.* 2004; Kleine *et al.* 2009). During the formation of present-day eukaryotes, much of the original DNA from the endosymbiont was transferred to the nuclear genome and what remains in the organelle is the current mitochondrial genome. The process of mitochondrial DNA (mtDNA) transfer is continual, although most integrations into chromosomes result in mtDNA sequences that become degraded over time (Richly and Leister 2004b). The existence of **nu**clear copies of **mt**DNA (NUMTs) has been observed in humans, animals, plants, fungi, and protists (Bensasson *et al.* 2001; Hazkani-Covo *et al.* 2010). These NUMTs provide a source of chromosomal diversity and contribute to genome evolution (Ricchetti *et al.* 2004, Lough *et al.* 2008, Martis *et al.* 2012).

Intraspecific variation of NUMTs has been reported from genomic sequence analyses in a small number of species. In humans, 12 of 40 examined NUMTs were identified as polymorphic (Hazkani-Covo *et al.* 2010), all of them smaller than 1228 bp (Yuan *et al.* 1999; Giampieri *et al.* 2004; Ricchetti *et al.* 2004). In contrast, little variation was detected among 21 NUMTs examined in 27 strains of the yeast *Debaryomyces hansenii* (Jacques *et al.* 2010). All of these were less than 400 bp (Sacerdot *et al.* 2008). In Arabidopsis (*Arabidopsis thaliana*), a 3.9-kb NUMT was reported to be present within the polyubiquitin gene UBQ13 in the Columbia ecotype (Sun and Callis 1993) and three additional ecotypes (Ullrich *et al.* 1997); however, the NUMT was not present in this gene in five other ecotypes examined (Sun and Callis 1993). A different 104-bp NUMT was identified at the subtelomere region of the short arm of chromosome 1 (1S) in the Columbia ecotype (Kuo *et al.* 2006). Approximately one-third of 35 Arabidopsis accessions examined contained this NUMT (Kuo *et al.* 2006).

The examination of larger NUMTs can be challenging because mtDNA sequences may be discarded as contamination during the assembly process of nuclear genomes. Therefore, even in “fully sequenced” nuclear genomes, NUMTs may not be entirely documented. For example, in Arabidopsis, sequence analysis identified a ∼270-kb NUMT near the centromere of chromosome 2S (Lin *et al.* 1999); however, cytological data determined that the NUMT was actually ∼620 kb in length (Stupar *et al.* 2001). Sequence data alone are likely to underestimate the number, sizes, and variation of NUMTs within a species.

Maize (*Zea mays* ssp. *mays*) contains an immense amount of genetic and phenotypic diversity (Buckler *et al.* 2006; Yu *et al.* 2008), which is ideal for studying intraspecific variation. Maize breeders keep detailed records of the lines used to form new inbreds and select for specific traits. For this important crop plant, a reference nuclear genome has been sequenced from the inbred line B73 (Schnable *et al.* 2009). In addition, multiple mitochondrial genomes have been fully sequenced from maize and related grasses (Clifton *et al.* 2004; Allen *et al.* 2007; Darracq *et al.* 2010). Furthermore, a set of tools exists for karyotyping chromosomes from any inbred maize line with fluorescence *in situ* hybridization (FISH; Kato *et al.* 2004). Thus, maize provides an ideal opportunity to study intraspecific variation of NUMTs using a combination of cytogenetic and sequence analysis methods.

The present study examines NUMT variation in a set of inbred lines that capture the diversity of maize. The presence of a large NUMT was found at a similar location on the long arm of chromosome 9 (9L) in four distantly related lines. Further investigation of this NUMT in B73 was possible because the B73 reference nuclear genome has been sequenced (Schnable *et al.* 2009). In addition to analyzing sequences, the B73 9L NUMT has been examined with the use of FISH and fiber-FISH. This work has led to a clearer picture of the mtDNA content and size of the 9L NUMT and also illustrates the benefits of complementing sequence analysis with cytogenetics.

## Materials and Methods

### Inbred lines

**Figure 1:**
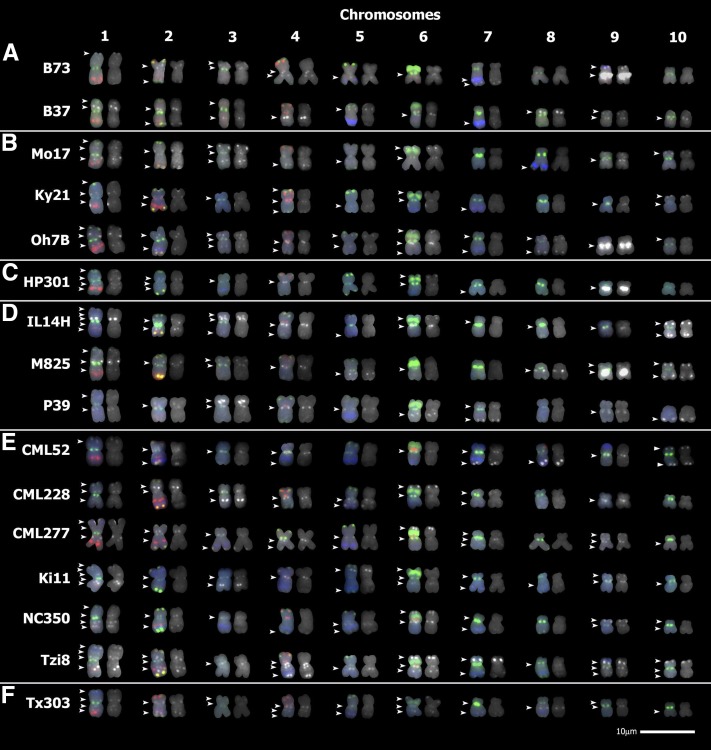
Mitochondrial DNA (mtDNA) insertion sites in diverse maize lines. mtDNA was hybridized to the chromosomes of diverse maize lines using a 19-cosmid mix probe. Sites of mtDNA hybridization are shown in white. Chromosomes were identified using a mix of eight karyotyping probes (shown in color). Chromosome on left: karyotyping probes and mtDNA probes. Chromosome on right: mtDNA probes only. White arrowheads indicate mtDNA insertions. (A) Stiff stalk maize lines. (B) Nonstiff stalk maize lines. (C) Popcorn maize line. (D) Sweet corn maize lines. (E) Tropical/subtropical maize lines. (F) Maize line of mixed background. Chromosomes from the lines B73, B37, and Mo17 are reproduced with permission (from Genetics; Lough *et al.* 2008). Scale = 10 μm.

Each of the 16 inbred lines examined in the diversity screening is a founder line of the maize nested association mapping population (Yu *et al.* 2008), with the exception of B37, Mo17, and M825 (Figure 1). At least one line from each maize genetic subgroup has been included: two stiff stalk lines (B73 and B37); three nonstiff stalk lines (Mo17, Ky21, and Oh7B); one popcorn line (HP301); three sweet corn lines (IL14H, M825, and P39); six tropical/subtropical lines (CML52, CML228, CML277, Ki11, NC350, and Tzi8); and one mixed line (Tx303). Additional lines from the M825 pedigree also were examined, including Ia5125, R825, Wf9, and IP39.

### Cytogenetic and sequence analyses

**Figure 2:**
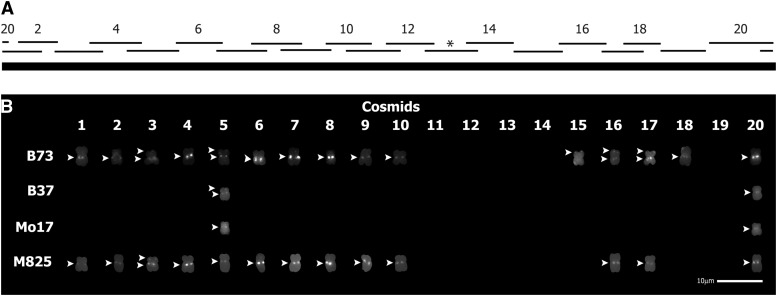
NB mitochondrial genome sections present at chromosome 9L mitochondrial DNA (mtDNA) insertions. (A) Simplified map of cosmids containing NB maize mtDNA (Lough *et al.* 2008). Upper cosmids are labeled with their corresponding number. Asterisk indicates cosmid 13, which contains approximately 16.7 kb of chloroplast DNA. (B) Individual mtDNA-containing cosmid probes hybridized to chromosomes from four inbred lines. Sites of mtDNA hybridization are shown in white. White arrowheads indicate mtDNA insertions. Only chromosome 9 is shown. Scale = 10 μm.

The FISH protocols used for preparing slides of root tip chromosome spreads and the labeling of both karyotyping and cosmid probes were described previously by Lough *et al.* (2008). Alterations to those methods are detailed in this paragraph. The karyotyping probes used to identify chromosomes included eight regions of repetitive DNA (Kato *et al.* 2004) that were made using the fluorescence-labeled nucleotides: Cascade Blue-7-dUTP, Alexa Fluor 488-5-dUTP, or Cyanine 5-dUTP (Cy5). mtDNA-containing cosmid probes were produced using segments of the NB maize mitochondrial genome and were labeled with Texas Red-5-dCTP (Lough *et al.* 2008). The 570-kb NB mitochondrial genome was sequenced from the B37 stiff stalk line (Clifton *et al.* 2004) and also is present in B73. These mtDNA probes are either 20 individually labeled segments of the mitochondrial genome (Supporting Information, Table S1) or a combination of segments in a 19-cosmid mix probe covering nearly the entire mitochondrial genome (Figure 2A). Cosmid 13 is not included in this mix of 19 segments because it contains plastid DNA (Lough *et al.* 2008). The 2.4- and 3.3-kb probes also were labeled with Texas Red-5-dCTP. The 2.4-kb region primers were designed based on the bacterial artificial chromosome (BAC) AC183911 sequence, and the 3.3-kb region primers were designed based on BACs AC187467 and AC183911 sequences (Table S2). Mitotic metaphase chromosome spreads were obtained from root tips digested with cellulase and pectolyase. The fiber-FISH procedures used for preparing slides, making labels, hybridizing slides, capturing FISH images, and adjusting images in this study were defined previously by Koo *et al.* (2011). To detect mtDNA insertions on B73 DNA fibers, different combinations of a two-color fiber-FISH procedure were used. The first combination included only labels of mtDNA and the second combination included mtDNA and methylation labels (see supporting information in File S1). The maize NB mitochondrial genome sequence was compared with the nuclear reference genome by use of the ZeAlign program available through Maize Genetics and Genomics Database (MaizeGDB; Sen *et al.* 2010). All of the data presented here was produced with the B73 maize reference nuclear genome assembly version 2. The resulting files from this comparison were uploaded as a custom track on the maize genome browser at MaizeGDB to visualize the mtDNA insertions relative to the chromosome 9 centromere, BACs, and the gene models (Sen *et al.* 2010). The nuclear gene models were given a functional identification based on the location of corresponding mitochondrial genes in the NB genome (Clifton *et al.* 2004), and identification of protein domains using InterProScan (Zdobnov and Apweiler 2001). Retrotransposons were detected with the program RepeatMasker (Smit *et al.* 1996−2010).

### Data availability

Sequence data are available at MaizeGDB and GenBank. GenBank mitochondrial genome accessions used in this analysis (Clifton *et al.* 2004; Allen *et al.* 2007): NB maize [National Center for Biotechnology Information (NCBI) accession no. AY506529.1], NA maize (NCBI accession no. DQ490952.1), CMS-S maize (NCBI accession no. DQ490951.2), CMS-T maize (NCBI accession no. DQ490953.1), and *Zea mays* ssp. *parviglumis* (NCBI accession no. DQ645539.1). Further details about the materials and methods can be found in File S1.

## Results

### Screening a set of diverse maize inbred lines for NUMTs

The NB mitochondrial genome used in this study was previously sequenced from the line B37 (Clifton *et al.* 2004), and it is present in many other inbred lines, including B73 and Mo17. For the NUMT analyses, maize inbred lines representing the six different genetic subgroups of maize (Flint-Garcia *et al.* 2005; Yu *et al.* 2008) were selected to survey NUMT variation using the mtDNA-containing 19-cosmid mix probe (Figure 1). Two lines are shown from the temperate stiff stalk subgroup created to select plants for high stalk quality (Troyer 2001): B73 and B37 (Figure 1A). Three lines from the temperate nonstiff stalk subgroup were examined: Mo17, Ky21, and Oh7B (Figure 1B). One popcorn line, HP301, was included in this survey (Figure 1C). Three sweet corn lines were analyzed: IL14H, M825, and P39 (Figure 1D). Six different tropical/subtropical inbred lines were chosen for this analysis: CML52, CML228, CML277, Ki11, NC350, and Tzi8 (Figure 1E). Lastly, one line of mixed origin, Tx303, was included (Figure 1F).

Variation in the number and location of NUMTs was observed in this set of lines by the use of FISH (Figure 1). The lowest number of NUMTs found was in the sweet corn line P39, which had only 12 detectable NUMTs. The greatest number of NUMTs found in a single line was in Tzi8, a tropical line that contained 24 detectable NUMTs. This number exceeds the largest number of NUMTs previously observed in a maize line using FISH, which was 19 NUMTs in the nonstiff stalk line Oh43 (Lough *et al.* 2008). Chromosome 1 in the lines IL14H and Ki11 contains five NUMTs, which is the greatest number of NUMTs detected on a single maize chromosome using FISH. Consistent with our previous publication (Lough *et al.* 2008), a NUMT is present at a similar location on chromosome 2S near the centromere in every line examined (Figure 1). In the lines analyzed here, at least one NUMT was observed on chromosomes 1−4, 6, and 9. Among the 20 maize lines for which NUMT observations have been published, nine lines have no detectable NUMTs on chromosome 8 (Figure 1; see also Lough *et al.* 2008). The lines B37, Oh7B, IL14H, Ki11, Tzi8, and Tx303 have at least one detectable NUMT on every chromosome.

In this set of maize inbred lines, a NUMT with a strong hybridization signal was observed near the centromere of 9L in a total of four lines (Figure 1): B73 (stiff stalk), Oh7B (nonstiff stalk), HP301 (popcorn), and M825 (sweet corn). Thus, in addition to the previously published large 9L NUMT in B73 (Lough *et al.* 2008), three comparable 9L NUMTs have been identified in lines from different genetic subgroups of maize. All other lines analyzed here have a chromosome 9L proximal NUMT with a much weaker hybridization signal.

### Cytogenetic characterization of the chromosome 9L NUMT in four maize lines

To further analyze the NUMTs near the centromere on the long arm of chromosome 9, a detailed examination of the NUMT region in four inbred lines was completed using the 20 individual mtDNA-containing cosmid probes (Figure 2A). The previously examined B73 large NUMT (Lough *et al.* 2008) is compared here with the 9L NUMT in M825, B37, and Mo17. M825 was chosen because it contains a strong NUMT signal on 9L (Figure 1). B37 and Mo17 were chosen because they contain a weaker NUMT signal at a similar position on 9L (Figure 1).

A majority of the 20 individual mtDNA-containing cosmid probes (Figure 2A) hybridized to the chromosome 9L NUMT region of the two lines with the strong hybridization signal, B73 and M825 (Figure 2B). Fourteen of the 20 probes hybridized to the B73 9L NUMT, and 13 of these probes also hybridized to the M825 9L NUMT. The mtDNA contained within cosmid 18 was present in the B73 NUMT but not in the M825 NUMT. To test whether the NUMT could be at a similar site on chromosome 9 in both B73 and M825, a preliminary segregation analysis was performed on F2 progeny from a cross between B73 and M825 (Figure S1). No evidence of recombination was seen in 92 meiotic events, which would be indicated by a chromosome with either no NUMT or a doubled site. This lack of recombination suggests that the 9L proximal NUMTs in B73 and M825 are present at the same or very closely linked sites.

The two lines with a weaker chromosome 9L NUMT signal, B37 and Mo17, were examined for the extent of the mitochondrial genome present at that location. Only two of the mtDNA-containing cosmid probes (5 and 20) hybridized to the 9L NUMT in these lines (Figure 2B). Cosmid 5 and 20 probes hybridized to all four lines examined (Figure 2B). If the strong (B73) and weak (Mo17) signal NUMTs were located at different positions on chromosome 9L, then recombination could result in chromosomes containing no NUMT. A preliminary analysis performed on recombinant inbred lines derived from a cross between B73 and Mo17 found no evidence for such recombination (Figure S2).

### Variation in the 9L NUMT region in a subset of the maize diversity lines

**Figure 3:**
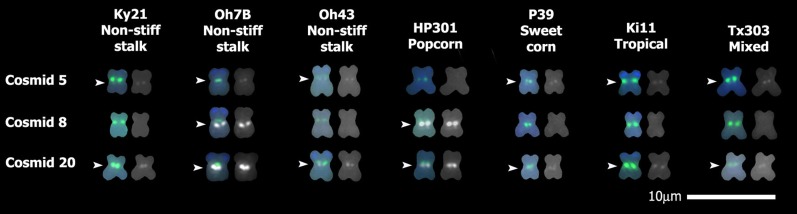
Variation in the 9L nuclear copies of mtDNA (NUMT) region in a subset of the maize diversity lines. Probes of mitochondrial DNA (mtDNA)-containing cosmids 5, 8, or 20 were hybridized to chromosomes from a diverse group of inbred lines. Cosmid 8 was used as a diagnostic tool for the large 9L NUMT (with a strong hybridization signal). Only chromosome 9 is shown. Sites of mtDNA hybridization are shown in white. Chromosomes were identified using a mix of three probes: Cent C, Knob, and 4-12-1 (shown in color). Chromosome on left: karyotyping probes and mtDNA probes. Chromosome on right: mtDNA probes only. White arrowheads indicate mtDNA insertions. Scale = 10 μm.

After observing that mtDNA present within cosmid probes 5 and 20 (Figure 2A) hybridized to a similar location on chromosome 9L in B73, B37, Mo17, and M825, the hybridization of these probes in a more diverse group of maize lines was examined (Figure 3). Seven lines were chosen for this investigation: Ky21, Oh7B, Oh43, HP301, P39, Ki11, and Tx303. Oh43 was previously analyzed using the 19-cosmid mix and found to contain a weak signal on chromosome 9L proximal (Lough *et al.* 2008).

On the basis of the detailed analysis with B73 and M825, the cosmid 8 probe was selected as a diagnostic tool for the presence of the strong NUMT signal at this location. Indeed, the cosmid 8 probe hybridized only to the strong signal 9L NUMTs: B73, M825, Oh7B, and HP301 (Figure 2B and Figure 3). The cosmid 20 probe hybridized to the 9L NUMT region for all tested lines, regardless of whether the NUMT showed a strong or weak hybridization signal when we used the 19-cosmid mix probe (Figure 2B and Figure 3). The cosmid 5 probe hybridized to the 9L NUMT region of every tested line except HP301 (Figure 2B and Figure 3). This absence of cosmid 5 probe hybridization indicates that there is at least one difference between the strong hybridization signal 9L NUMT of HP301 and the other three lines with similar 9L NUMT signals (B73, M825, and Oh7B).

### NUMTs within lines contributing to M825

**Figure 4:**
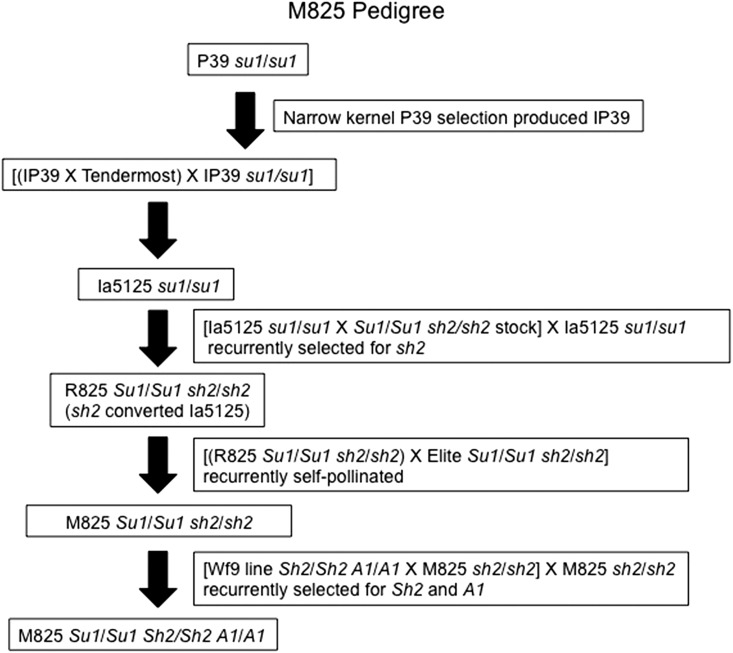
Pedigree of the sweet corn-derived line M825. Listed here are the members of the M825 pedigree tracing to the earliest member included in this study, the inbred line P39. Also included are the observed genotypes for the *sugary1* (*su1*), *shrunken2* (*sh2*), and *anthocyaninless1* (*A1*) maize genes.

The lineage studied here follows the development of the sweet corn−derived line M825 (Figure 4). M825 was chosen because it contains a large-sized NUMT on chromosome 9L (Figure 1), and many of the lines that contributed to its pedigree are still available.

**Figure 5:**
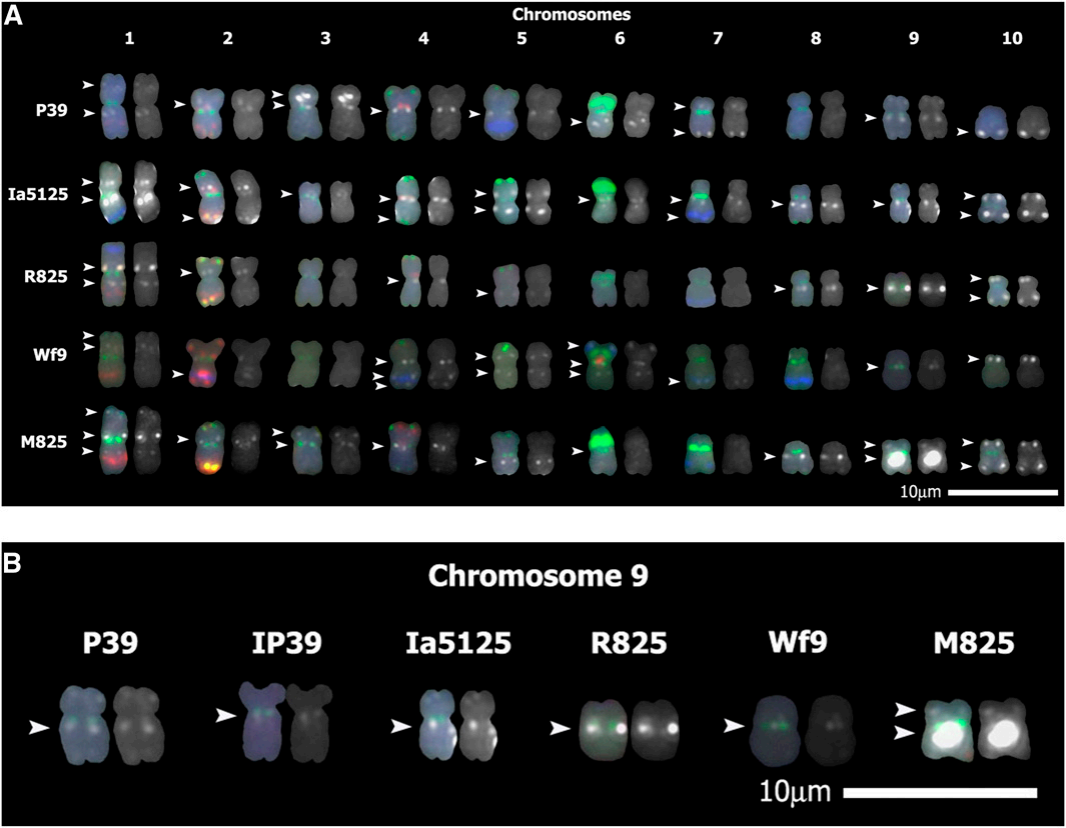
Nuclear copies of mtDNA (NUMTs) observed in lines contributing to the M825 pedigree. The Texas Red−labeled 19-cosmid mix was used with a mix of eight karyotyping probes to identify NUMTs on mitotic metaphase chromosomes of maize lines. Chromosome sites of hybridization with mitochondrial DNA (mtDNA) are shown in white; karyotyping probes shown in color. Chromosome on left: karyotyping probes and mtDNA probes. Chromosome on right: mtDNA probes only. White arrowheads indicate mtDNA insertions. (A) All 10 chromosomes from five inbred lines in the M825 pedigree are shown. (B) Chromosome 9 only is shown from each of six inbred lines in the M825 pedigree. Scale = 10 μm.

All 10 chromosomes of five of the lines contributing to the M825 pedigree were examined for NUMTs with the 19-cosmid mix probe (Figure 5A). In all five lines, there are two insertion sites at the same approximate location: a similarly sized NUMT on chromosome 4L near the centromere and a variably sized NUMT at the 9L site. In several lines, other NUMTs appear to be at comparable positions. For example, in all lines but Wf9, there is a NUMT on chromosome 2S near the centromere and chromosome 10L near the telomere. There are also NUMTs present in several lines on the proximal part of chromosome arm 1L, on the interstitial region of 5L, near the centromere of 8L, and near the telomere of 10S.

Chromosome 9 was examined in six lines of the M825 pedigree with the 19-cosmid mix probe (Figure 5B). The NUMTs located at approximately the same position near the centromere of chromosome 9L in these lines were compared. The NUMTs in lines P39, IP39, and Wf9 have a weak hybridization signal. The NUMT present in R825 appears to have a slightly stronger signal than the previously mentioned lines, indicating a possible increase in the amount of mtDNA present at that location. M825, however, contains a NUMT of strong signal strength, indicating a large amount of mtDNA at that position. The data presented in Figure 2B indicate most of the mitochondrial genome is present in this M825 NUMT. On the basis of the M825 pedigree (Figure 4), a contributing line from which the large 9L site might have originated could not be documented in our studies. In contrast, Supergold, one of the progenitor strains for the popcorn line HP301, has a strong hybridization signal on 9L (Figure S3). Thus, Supergold is the likely origin of the HP301 9L NUMT.

### Sequence analysis of the B73 chromosome 9L NUMT

The cytogenetic location of the B73 9L NUMT was further analyzed by hybridizing fluorescently labeled probes onto meiotic pachytene chromosome spreads. Using fluorescently labeled mtDNA cosmid probes (cosmids 3 and 9) together with 9L-specific BAC and gene probes (Danilova and Birchler 2008), we confirmed that the NUMT is located between the *glossy15* (*gl15*) locus and the centromere (Figure S4). In the sequence assembly (version 2) of B73 chromosome 9, *gl15* is reported to be between 95,739,338 and 95,742,681 bp and the chromosome 9 centromere is estimated to be between 72,200,000 and 72,700,000 bp (Sen *et al.* 2010). Therefore, we could narrow our search for the NUMT sequences on 9L to this 23 Mb region.

The maize NB mitochondrial genome (Clifton *et al.* 2004) was compared with the maize reference nuclear genome (version 2) with the ZeAlign program at MaizeGDB (Sen *et al.* 2010). The mtDNA insertions identified by the use of ZeAlign were found across a ∼302-kb region of the nuclear genome (chromosome 9: 72,709,800−73,012,289 bp). Of the ∼302 kb in this region, ∼252 kb of mtDNA was identified as a series of discontinuous NUMTs (Table 1). Disparate regions of the NB mitochondrial genome were found adjacent to each other in the nuclear sequence (*e.g.*, mtDNA contained within cosmid 20 adjacent to cosmid 10 from 72,911,607 to 72,920,084 bp on chromosome 9). The longest, continuous, unrearranged fragment of mtDNA is 80,240 bp, and the shortest length is 56 bp. The majority of the continuous NUMT pieces are less than 7 kb. Percent identities between the 9L NUMT and the NB mitochondrial genome ranged from 93.81 to 100% (with the exception of the 2.4- and 3.3-kb regions discussed in *Comparisons of B73 NUMT sequence to mitochondrial genomes*). The presence of many NUMT segments less than 1 kb is common in nuclear genomes, such as in Arabidopsis, where the average NUMT size is only 346 bp (Richly and Leister 2004a).

**Table 1:** Locations of NB mtDNA in B73 chromosome 9L NUMT region

Cosmid Number / 2.4 kb / 3.3 kb	Length, bp	Nuclear Start Position, bp	Nuclear End Position, bp	NB Start Position, bp	NB End Position, bp	% Identity	e-Value
6	210	72710009	72709800	138491	138689	93.81	5.00E-79
20-1	171	72710160	72709990	564633	564803	98.83	2.00E-81
3.3 kb region	3350	72710161	72713511	−	−	−	−
9	78	72710437	72710361	236657	236730	89.74	2.00E-11
20	19072	72713512	72732571	536974	556020	99.72	0
2.4 kb region	2398	72732572	72734970	−	−	−	−
4-5	77	72733754	72733830	101485	101561	96.10	3.00E-25
1-2	81	72734179	72734251	21426	21506	87.65	2.00E-12
16	81	72734179	72734251	433322	433402	87.65	2.00E-12
9	56	72734290	72734235	237278	237333	96.43	8.00E-15
6	72	72734378	72734307	139157	139228	97.22	5.00E-24
17	1788	72736758	72734971	452232	454019	100.00	0
17	2181	72746025	72743845	450057	452237	99.95	0
16-17	18871	72768137	72749281	431199	450061	99.84	0
16	12891	72788398	72775508	418316	431203	99.91	0
8-10	32001	72820411	72788411	218668	250632	99.82	0
6-9	80240	72908103	72827872	138491	218672	99.84	0
20-1	171	72908254	72908084	564633	564803	98.83	2.00E-81
3.3 kb region	3351	72908255	72911606	−	−	−	−
9	78	72908531	72908455	236657	236730	88.46	1.00E-09
20	6781	72911607	72918381	536974	543749	99.81	0
10	1705	72918380	72920084	248929	250632	99.82	0
16-17	31759	72920097	72951839	418316	450061	99.84	0
16	684	72967167	72966486	431199	431881	99.27	0
16	656	72975193	72974538	430548	431203	99.70	0
17	2181	72977692	72979872	450057	452237	99.95	0
17	1788	72980457	72982244	452232	454019	100.00	0
2.4 kb region	2398	72982245	72984643	−	−	−	−
6	72	72982837	72982908	139157	139228	97.22	5.00E-24
9	56	72982925	72982980	237278	237333	96.43	8.00E-15
16	81	72983036	72982964	433322	433402	87.65	2.00E-12
1-2	81	72983036	72982964	21426	21506	87.65	2.00E-12
4-5	77	72983461	72983385	101485	101561	96.10	3.00E-25
20	19068	73003698	72984644	536974	556020	99.75	0
3.3 kb region	3350	73003699	73007049	−	−	−	−
9	78	73006773	73006849	236657	236730	89.74	2.00E-11
20-1	171	73007050	73007220	564633	564803	98.83	2.00E-81
6	5089	73007201	73012289	138491	143568	99.67	0

Locations of mtDNA on B73 chromosome 9L near the centromere were identified using the ZeAlign program available through MaizeGDB (Sen *et al.* 2010). Shown here is the region of the B73 nuclear genome sequence (version 2) starting with the most proximal segment of NB mtDNA (National Center for Biotechnology Information accession no. AY506529.1) and ending with the most distal segment of NB mtDNA contained in this NUMT region. The cosmid(s) corresponding to each mtDNA segment is included. The locations of the two copies of the 2.4-kb region are labeled. The locations of the three copies of the 3.3 kb region are labeled. Intact 2.4- and 3.3-kb regions do not exist in the NB mitochondrial genome (indicated by a dash in the table); only short fragments of these sequences are present in the NB mtDNA-containing cosmids. The cosmids containing such fragments are indented beneath the 2.4- and 3.3-kb region labels. mtDNA, mitochondrial DNA; NUMT, nuclear copies of mtDNA.

Multiple retrotransposons were identified throughout the 9L NUMT region (Table S3). Four retrotransposons in the 9L NUMT region had identifiable long terminal repeats (LTRs) that matched with 100% identity when compared using the Align Sequences Nucleotide BLAST program (Altschul *et al.*1990; Camacho *et al.* 2008). The identical LTRs present in these retrotransposons indicate that they are very recent insertions (SanMiguel *et al.* 1998; Miclaus *et al.* 2011). These retrotransposons are: a Gypsy LTR retrotransposon found at 72,736,759−72,743,845 bp with an LTR that is 584 bp long; a Gypsy LTR retrotransposon at 72,768,138−72,775,507 bp with a 669-bp LTR; a Gypsy LTR retrotransposon at 72,820,412−72,827,871 bp with a 663-bp LTR; and a Gypsy LTR retrotransposon at 72,967,168−72,974,537 bp with a 669-bp LTR. Each of the LTRs of these retrotransposons is flanked by a 5- to 6-bp target site duplication of mtDNA (Table 1). Short target-site duplications are commonly found flanking LTR retrotransposons (Gao *et al.* 2012). These duplications occur during the insertion of a retrotransposon because of double-strand breaks that are staggered (Linheiro and Bergman 2012). Retrotransposons that interrupt segments of mtDNA in the 9L NUMT region create discontinuous stretches of mtDNA from segments that were previously long and continuous. If the retrotransposons are removed, a continuous stretch of mtDNA is present.

The ∼302 kb NUMT region was scanned for protein-coding genes from the NB mitochondrial genome. Twelve protein-coding gene models were functionally annotated (Table 2): 1) using InterProScan to identify protein domains with a known function (Zdobnov and Apweiler 2001); and 2) comparing the gene model locations to corresponding genes in the NB mitochondrial genome (Clifton *et al.* 2004).

**Table 2:** Gene models within the B73 9L NUMT region identified as mitochondrial protein-coding genes

Gene ID	Length, bp	Nuclear Start Position, bp	Nuclear End Position, bp	NB Start Position, bp	NB End Position, bp	Functional Annotation of Gene Models
GRMZM5G820250	940	72756833	72757773	442509	441569	Cytochrome c oxidase subunit 3 (cox3)
GRMZM5G863208	1462	72765346	72766808	433996	432534	Ribosomal protein S7 (rps7)
GRMZM5G889138	5997	72795479	72801476	243564	237567	NADH dehydrogenase subunit 7 (nad7)
GRMZM5G827449	2481	72839436	72841917	207108	204627	NADH dehydrogenase subunit 5 (nad5)
GRMZM2G407837	3404	72844969	72848373	201575	198171	Cytochrome c biogenesis C (ccmC)
GRMZM2G109550	3928	72883368	72887296	163176	159248	Ribosomal protein L16 (rpl16)
GRMZM2G109418	1689	72906347	72908036	140197	138508	ATPase subunit 4 (atp4)
GRMZM2G109332	2258	72916000	72918258	541367	543625	Cytochrome c oxidase subunit 2 (cox2)
GRMZM5G880319	446	72935328	72935774	433547	433993	Ribosomal protein S7 (rps7)
GRMZM2G156536	2853	72942112	72944965	440331	443184	Cytochrome c oxidase subunit 3 (cox3)
GRMZM2G142109	2588	72997120	72999708	543544	538460	Cytochrome c oxidase subunit 2 (cox2)
GRMZM2G470035	665	73007268	73007933	138558	139223	ATPase subunit 4 (atp4)

These gene models were functionally annotated using InterProScan (Zdobnov and Apweiler 2001), and the locations of genes in the NB mitochondrial genome are shown. NUMT, nuclear copies of mtDNA; NADH, nicotinamide adenine dinucleotide.

The B73 reference genome sequence for the 9L NUMT (Table 1) was compared with the preceding FISH data to determine whether all of the mitochondrial genome segments observed at this location with the use of FISH are represented in the nuclear sequence (version 2). The reliable detection of FISH signals on metaphase chromosomes has a lower limit of ∼2−3 kb (Yu *et al.* 2007), which means that only stretches of mtDNA within the NUMT that are at least that long can be compared with the FISH data. FISH analyses previously published reported that mtDNA-containing cosmids 1−10, 16−18, and 20 hybridized to the B73 9L NUMT (Figure 2B; Lough *et al.* 2008). Compared with the available nuclear sequence, only the mtDNA within cosmids 1, 3−4, 6−10, 16, 17, and 20 is present in long enough stretches to be visible using FISH. However, the mtDNA within three other cosmid probes (2, 5, and 18) also hybridized to the B73 9L NUMT. These mtDNA sequences were each present at less than 100 bp in the reference nuclear genome (Table 1). This comparison of FISH data to the available nuclear sequence (version 2) shows that the sequence of the B73 9L NUMT is incomplete.

### Comparisons of the B73 NUMT sequence to mitochondrial genomes

The 9L NUMT sequence also was compared with fully sequenced *Zea* mitochondrial genomes. The mitochondrial genomes of maize include two normal fertile genomes, NB (Clifton *et al.* 2004) and NA (Allen *et al.* 2007), as well as three cytoplasmic male-sterile genomes, CMS-S, CMS-T, and CMS-C (Allen *et al.* 2007). The most common mitochondrial genome is NB, which is present in the B73 line. Sequenced mitochondrial genomes also are available for a number of teosintes, including *Zea mays* ssp. *parviglumis* (Zmp), which is believed to be the progenitor of maize (Doebley 2004). Phylogenetic analyses have determined that the NA and Zmp mitochondrial genomes are very closely related (Darracq *et al.* 2010), agreeing with the recent domestication (∼9000 years ago) of maize (Piperno and Flannery 2001; Matsuoka *et al.* 2002; Yamasaki *et al.* 2007).

Cytogenetic data described previously indicate that sections of the NB mitochondrial genome are missing from the 9L NUMT in the B73 reference genome version 2 sequence; however, within the available NUMT sequence, we identified mtDNA originating from other mitochondrial genomes. The B73 9L NUMT sequence includes copies of mtDNA segments that are ∼2.4 and 3.3 kb in length. There are two identical copies of the 2.4-kb region in the 9L NUMT. There are three copies of the 3.3-kb region in the 9L NUMT. The first and third copies are identical to each other, but the second copy differs slightly (5/3352 nucleotide differences). These segments are mtDNA, but they do not appear to have originated from NB, the mitochondrial genome present in the B73 inbred line (Table 1).

The 2.4- and 3.3-kb regions from the NUMT sequence were then compared with other sequenced *Zea* mitochondrial genomes using NCBI BLAST (bl2seq; Altschul *et al.* 1990; Camacho *et al.* 2008) and ClustalW2 (McWilliam *et al.* 2013). The 2.4-kb region is present within both the NA (2/2399 nucleotide differences) and Zmp (3/2399 nucleotide differences) mitochondrial genomes and most closely matches the NA genome (Figure S5). The 3.3-kb region is present in the NA, Zmp, CMS-S, and CMS-T mitochondrial genomes. The first and third copies of the 3.3-kb region have 3/3352 nucleotide differences with NA, Zmp, and CMS-S, and 12/3357 nucleotide differences with CMS-T. The second copy of the 3.3-kb region has 2/3352 nucleotide differences with NA, Zmp, and CMS-S, and 11/3357 nucleotide differences with CMS-T. These sequence comparisons indicate that the second 3.3-kb region is more similar to the NA, Zmp, and CMS-S mitochondrial genomes than the first and third copies of the 3.3-kb region (Figure S6).

The 2.4-kb region is intergenic in both the NA and Zmp mitochondrial genomes. The 3.3-kb region is intergenic within the NA, Zmp, and CMS-S mitochondrial genomes. In the CMS-T mitochondrial genome,165 bp of the 3.3-kb region overlaps the end of a chimeric orf (orf118-b).

### Presence of the 2.4- and 3.3-kb mtDNA regions on 9L of inbred lines

**Figure 6:**
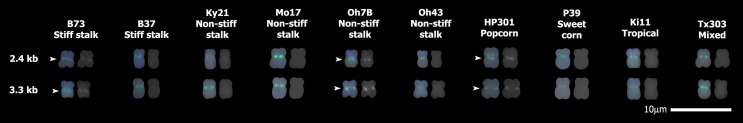
Two mitochondrial DNA (mtDNA) regions (2.4- and 3.3-kb) within the B73 9L genomic sequence but missing from the NB mitochondrial genotype are associated with strong hybridization signals on the 9L nuclear copies of mtDNA (NUMTs). Genomic sequences within the B73 9L NUMT match two regions of *Zea* mitochondrial genomes that are not present in the B73 NB mitochondrial genome. A Texas Red−labeled fluorescence *in situ* hybridization probe of the 2.4-kb or 3.3-kb region was hybridized to chromosomes from inbred lines. Only chromosome 9 is shown. The chromosome on the left shows both the Cent C and knob probes (color) and the 2.4- or 3.3-kb probe in white. The chromosome on the right shows only the 2.4- or 3.3-kb probe layer. White arrowheads indicate mtDNA insertion sites. Scale = 10 μm.

FISH probes were prepared for the 2.4- and 3.3-kb regions and hybridized separately to chromosomes of 10 inbred lines (Figure 6), including eight nested association mapping parental lines (Yu *et al.* 2008), B37, and Mo17. These lines represent the diversity of maize: B73 (stiff stalk), B37 (stiff stalk), Ky21 (nonstiff stalk), Mo17 (nonstiff stalk), Oh7B (nonstiff stalk), Oh43 (nonstiff stalk), HP301 (popcorn), P39 (sweet corn), Ki11 (tropical), and Tx303 (mixed). The 2.4- and 3.3-kb region probes only hybridized to 9L of lines that were observed to have a relatively large NUMT near the centromere of 9L: B73, Oh7B, and HP301 (Figure 6, also Figure 1). Because the 2.4- and 3.3-kb regions are present in the NA genome, the use of these probes creates cytoplasmic background (hybridization to mtDNA originating from ruptured mitochondria) in lines with the NA genome (*e.g.*, Ky21; Figure S7).

### Fiber-FISH analysis of the B73 chromosome 9L NUMT

**Figure 7:**
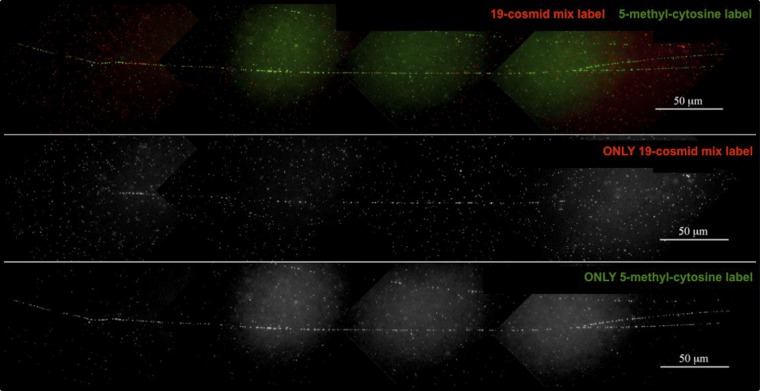
Fiber-fluorescence *in situ* hybridization (FISH) analysis shows methylation of the ∼1.8 Mb B73 chromosome 9L nuclear copies of mtDNA (NUMT). B73 DNA fibers were examined by fiber-FISH using a 19-cosmid mix label (red) and a 5-methyl-cytosine label (green). After examining multiple chromatin fibers, we estimated the size of the B73 9L NUMT to be ∼1.8 Mb. Presence of the 5-methyl-cytosine label (green) mixed with the 19-cosmid mix label (red) indicates nearly complete methylation of this site. Top panel overlays red and green labels. Middle panel shows only the red label. Bottom panel shows only the green label. The fiber shown here is ∼500 μm (∼1500 kb). 1 μm = ∼3 kb (Cheng *et al.* 2002). Multiple images were captured and then aligned with the use of Adobe Photoshop.

The two-color fiber-FISH technique was used to: 1) determine whether the B73 9L NUMT is methylated; and 2) estimate the size of the B73 9L NUMT. Twenty-four DNA fibers were analyzed for methylation of the NUMT. Each DNA fiber showed a similar methylation pattern. The overlap in hybridization of the methylation (5-methyl-cytosine; green) and mtDNA (19-cosmid mix; red) labels indicates that the NUMT is methylated (Figure 7). To clearly define the length of the NUMT, we performed fiber-FISH using only mtDNA labels. The cosmids known to hybridize to the B73 9L NUMT (Figure 2) were used as labels: 1−7 (red), and 8−10, 16−18, and 20 (green). The measurement of intact DNA fibers has estimated the size of the 9L NUMT to be ∼1.8 Mb (1811.6 ± 229.3 kb, n = 5; Figure S8).

## Discussion

Using FISH, we show that the distribution of NUMTs varies greatly among the surveyed maize lines, which included members of each maize genetic subgroup. Because there is a lower limit of ∼2−3 kb for reliably detecting sites on maize metaphase chromosomes using FISH (Yu *et al.* 2007), even more variation in mtDNA insertion sites may exist than we have detected. A NUMT at approximately the same position on chromosome 2S near the centromere is present in every maize inbred line examined thus far except for Wf9 (Figure 1 and Figure 5B; also Lough *et al.* 2008). This 2S NUMT was likely lost from this location at some point during the formation of the Wf9 lineage. Alternatively, this NUMT could have accumulated mutations and/or transposable element insertions that would prevent FISH probes from hybridizing.

In addition to documenting the extensive variation of NUMTs in maize, we have also discovered mtDNA insertion sites that likely contain a majority of the maize mitochondrial genome at a similar location on the long arm of chromosome 9 in four inbred lines from different maize subgroups (Flint-Garcia *et al.* 2005): B73 (stiff stalk), Oh7B (nonstiff stalk), HP301 (popcorn), and M825 (sweet corn derivative). A weaker hybridization signal, corresponding to much less mtDNA, was detected in this region in all the other lines examined in this study (Figure 1) and most of the previously analyzed maize inbreds (Lough *et al.* 2008). Studies examining B73/M825 F2 individuals and the Intermated B73 × Mo17 recombinant inbred lines for recombination in this region have yet to separate the chromosome 9L NUMT sites present in different lines (Figure S1 and Figure S2). These results suggest that the detectable 9L NUMTs with a strong hybridization signal (B73 and M825) or weak hybridization signal (Mo17) are at approximately the same location.

The chromosome 9L NUMT is located near the centromere. Large segments of organellar DNA have been known to insert into chromosome regions near centromeres (Michalovova *et al.* 2013); examples include chloroplast DNA insertions in rice (Matsuo *et al.* 2005), a cucumber NUMT on chromosome 4 (Koo *et al.* 2010), and the ∼620 kb Arabidopsis chromosome 2 NUMT (Stupar *et al.* 2001). The relative absence of essential genes in these regions may allow NUMTs to enter the chromosome without creating harmful mutations (Matsuo *et al.* 2005; Kleine *et al.* 2009).

A detailed FISH examination of the chromosome 9L NUMT region was performed on lines with either a strong or weak NUMT signal with the use of 20 mtDNA-containing cosmid probes. Nearly all lines tested contain detectable mtDNA fragments at the 9L NUMT region corresponding to cosmids 5 and 20 (Figure 2 and Figure 3). Thus, some mtDNA at this 9L NUMT site may predate the domestication of maize. Identification of larger NUMTs in this region of a few lines could result from: 1) many different segments of the mitochondrial genome present at approximately the same chromosomal location; 2) many copies of only a few mtDNA segments; or 3) multiple copies of many mtDNA segments. The strong signal of the chromosome 9L NUMT in both B73 and M825 is at least partially due the presence of a majority of the NB maize mitochondrial genome at that location, rather than only repeats of shorter mtDNA sections (Figure 2B).

To trace the 9L NUMT within a pedigree, six lines in the M825 pedigree were examined using the 19-cosmid mix mtDNA probe. A much larger NUMT is clearly present at the M825 9L site than can be found in the other lines contributing to this pedigree (Figure 5). Differences in the probe strength and exposure times could affect the recorded signal strengths; however, attempts were made to maintain consistency with exposure times for each image. Our examination suggests that the amount of mtDNA at this 9L NUMT increased in the M825 lineage after the R825 line was developed but before the current version of M825. A simple explanation of this finding might be that the large insertion was introduced into the lineage from another inbred. However, our data provide no evidence for such an insertion in the available contributing lines. This lack of evidence does not rule out the possibility that the large NUMT was contributed by a line within the pedigree that is no longer available. If such a line existed, the mtDNA could have been lost from the 9L NUMT in the currently available progenitor lines (Sheppard and Timmis 2009). Alternatively, the analysis of the M825 pedigree raises the possibility that larger NUMTs can result from the addition of mtDNA to smaller preexisting NUMTs.

After mtDNA inserts into a chromosome, the resulting NUMT gradually accumulates mutations, including nucleotide substitutions and the insertion of other sequences, such as transposable elements, all of which contribute to the degradation of the NUMT over time (Ueda *et al.* 2005; Kleine *et al.* 2009; Michalovova *et al.* 2013). The presence of retrotransposons that have inserted into the NUMT can provide some indication of the NUMT age. Identical LTRs within a retrotransposon suggest recent integration (Miclaus *et al.* 2011). Accordingly, the identical LTRs in the retrotransposons within the B73 9L NUMT indicate that the retrotransposons inserted very recently.

Two mtDNA regions, 2.4 and 3.3 kb in length, have thus far shown detectable hybridization only to the 9L NUMTs with a strong hybridization signal (B73, Oh7B, and HP301). These segments of mtDNA are found only in certain maize mitochondrial genotypes, particularly NA and Zmp (from a progenitor of domesticated maize). The 2.4- and 3.3-kb regions of mtDNA found intact in the B73 9L NUMT are not present in the NB mitochondrial genome of the B73 line. The association between lines with a large 9L NUMT and the presence of the 2.4- and 3.3-kb regions suggests a possible common origin for the NUMT in these diverse lines. One hypothesis is that the 9L NUMT was generated before the development of these inbred lines and most of it has since been lost in other inbred lines. An alternative hypothesis is that the large 9L NUMTs arose independently in each of these diverse lines. It is also possible that the 2.4-kb and 3.3-kb regions became incorporated into the B73 9L NUMT through exposure to another line carrying the NA mitochondrial genome. However, it should be noted that only ∼252 kb of the NUMT is available in the B73 nuclear sequence data. Without the entire sequence of this NUMT, further details about the NUMT’s origin cannot be determined.

The B73 chromosome 9L NUMT has been examined in more detail using the available sequence data to complement the FISH and fiber-FISH methods. Our fiber-FISH data estimates the size of the B73 9L NUMT to be ∼1.8 Mb (Figure 7). This size is 3× greater than the previously published largest organellar DNA insertion site, which was ∼620 kb in Arabidopsis (Stupar *et al.* 2001). Additionally, FISH analysis using different segments of the ∼570-kb NB mitochondrial genome has indicated that a majority of this mitochondrial genome is present in the B73 9L NUMT (Lough *et al.* 2008). However, several segments of the NB mitochondrial genome shown to be present through FISH were missing from the available sequence data for this region of chromosome 9L. Indeed, only ∼252 kb of mtDNA is reported to be present within a ∼302-kb segment adjacent to the centromere. Collectively, it is clear that sequence data are missing for this large B73 NUMT.

Plant organelle DNA has very low levels of methylated DNA, whereas nuclear DNA can be methylated (Huang *et al.* 2005). Previous studies have found nuclear insertions of organellar DNA to be methylated in plants (Huang *et al.* 2005). Here we have shown that the large 9L NUMT in B73 is methylated (Figure 7).

The extensive variation of mtDNA insertion sites shown here illustrates the frequent and continuous nature of organellar DNA transfers to and losses from the nuclear genome. This variation demonstrates the impact of NUMTs on maize chromosomal diversity and genome evolution. As seen in the present work and previous publications (Stupar *et al.* 2001), obtaining a complete sequence for a large NUMT can be difficult. BACs that contain only mtDNA are often thought to be contamination and routinely are removed from the assembly process. To ensure that organellar DNA insertion sites are correctly represented in the sequence of nuclear genomes, cytogenetic techniques such as FISH and fiber-FISH should be used to complement sequence analyses.

## 

## Supplementary Material

Supporting Information
