# Supplementary material for: Cytogenetic and Sequence Analyses of Mitochondrial DNA Insertions in Nuclear Chromosomes of Maize
Source: G3 (Bethesda). 2015 Sep 1;5(11):2229–39. doi: 10.1534/g3.115.020677 (PMC4632043; doi:10.1534/g3.115.020677)
Supplement: Supporting Information [file supp_5_11_2229__index.html]

Cytogenetic and Sequence Analyses of Mitochondrial DNA Insertions in Nuclear Chromosomes of Maize — Supporting Information 

# Cytogenetic and Sequence Analyses of Mitochondrial DNA Insertions in Nuclear Chromosomes of Maize

## Supporting Information for Lough *et al.*, 2015

**Files in this Data Supplement:**

- Supporting Information - Figures S1-S8, File S1, Tables S1-S3, and References (PDF, 826 KB)
- Figure S1 - Test for recombination between the 9L NUMT in B73 and M825. (PDF, 399 KB)
- Figure S2 - Test for recombination between the 9L NUMT in B73 and Mo17 using recombinant inbred lines derived from a B73 x Mo17 F1 hybrid. (PDF, 93 KB)
- Figure S3 - The 9L NUMT HP301 is present in the progenitor line Supergold. (PDF, 114 KB)
- Figure S4 - The B73 9L NUMT is located between the centromere and 9L-specific FISH probes on pachytene chromosomes. (PDF, 108 KB)
- Figure S5 - Multiple sequence alignment of the two B73 2.4-kb NUMT regions with the corresponding NA and Zmp mitochondrial genome regions. (PDF, 63 KB)
- Figure S6 - Multiple sequence alignment of the three B73 3.3-kb NUMT regions with the corresponding NA, Zmp, CMS-S, and CMS-T mitochondrial genome regions. (PDF, 68 KB)
- Figure S7 - The 2.4- and 3.3-kb probes hybridized to the 9L NUMT in the B73 line. (PDF, 299 KB)
- Figure S8 - Measurement of the B73 9L NUMT using fiber-FISH. (PDF, 84 KB)
- File S1 - Supporting Materials and Methods (PDF, 89 KB)
- Table S1 - Segments of the NB mitochondrial genome within cosmids. (PDF, 59 KB)
- Table S2 - Primers used to amplify the 2.4 and 3.3 kb regions from BAC DNA. (PDF, 59 KB)
- Table S3 - Locations of retrotransposons in the B73 chromosome 9L NUMT region. (PDF, 61 KB)
