# Supplementary material for: Cytogenetic and Sequence Analyses of Mitochondrial DNA Insertions in Nuclear Chromosomes of Maize
Source: G3 (Bethesda). 2015 Sep 1;5(11):2229–39. doi: 10.1534/g3.115.020677 (PMC4632043; doi:10.1534/g3.115.020677)
Supplement: Supporting Information [file supp_g3.115.020677_020677SI.pdf]

G3: Genes | Genomes | Genetics

Cytogenetic and Sequence Analyses of Mitochondrial DNA Insertions in Nuclear Chromosomes of Maize

Ashley N. Lough[*,1], Kaitlyn M. Faries[*,2], Dal-Hoe Koo[†,3], Abid Hussain[*,4], Leah M. Roark[*], Tiffany L. Langewisch[*,5], Teresa Backes[*,6], Karl A. G. Kremling[*,7], Jiming Jiang[†], James A. Birchler[*], and Kathleen J. Newton[*,8]

[*]Division of Biological Sciences, University of Missouri, Columbia, MO 65211, [†]Department of Horticulture, University of Wisconsin - Madison, Madison, WI 53706

Authors' present addresses: [1]Division of Science and Mathematics, Central Methodist University, Fayette, MO 65248, [2]Department of Chemistry, Washington University, St. Louis, MO 63130, [3]Department of Plant Pathology, Kansas State University, Manhattan, KS 66506, [4]Department of Biology, Medgar Evers College, Brooklyn, NY 11225, [5]Plant Genetics Research Unit, USDA-Agricultural Research Service, University of Missouri; Columbia, MO 65211, [6]School of Medicine, University of Missouri, Columbia, MO 65211, [7]Department of Plant Breeding and Genetics, Cornell University, Ithaca, NY 14853

[8]Corresponding author: Division of Biological Sciences, University of Missouri, 105 Tucker Hall, Columbia, MO 65211. E-mail: NewtonK@missouri.edu

DOI: 10.1534/g3.115.020677

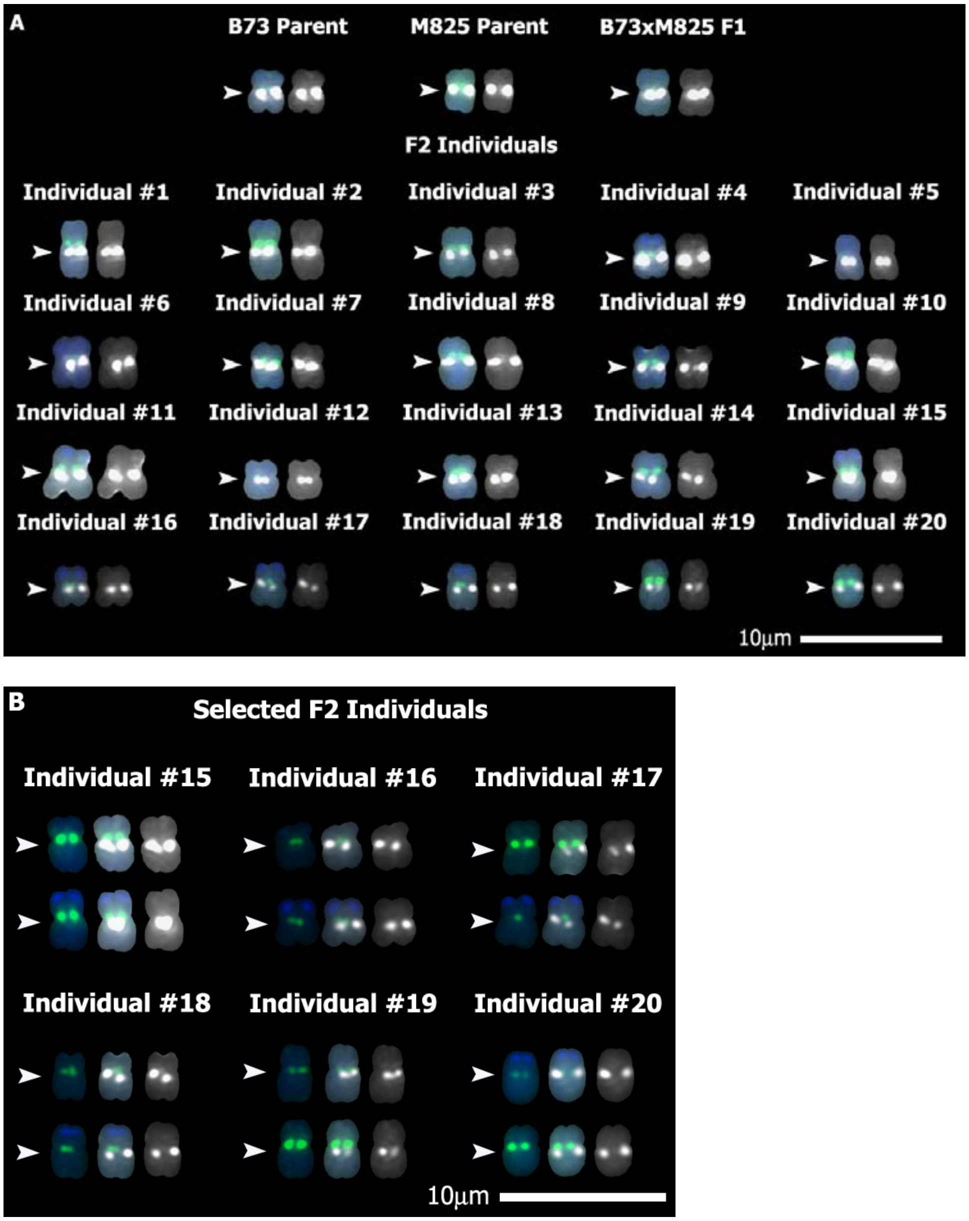

Figure S1 Test for recombination between the 9L NUMT in B73 and M825. F2 individuals from a self-pollination of a B73/M825 F1 hybrid were used in this study. If the NUMTs are located at different sites on chromosome 9L, then recombination could occur and chromosomes containing no NUMT or a doubly large NUMT would be found. At this level of resolution, there is no evidence of the recombination event. In the images shown here, cosmids 7 and 8 were used as probes because both were observed at the chromosome 9L NUMT of B73 and M825 with a clearly visible signal (Figure 2B). Initial concentrations are indicated for each probe with the volume used. Texas red-labeled probes of cosmids 7 and 8 (1 µl

each at 100 ng/μl) were used on slides of the first 20 F2 individuals examined. To identify chromosome 9 specifically, three karyotyping probes were used per slide: 0.35 μl Alexa Fluor 488-labeled Cent C (50 ng/μl), 0.5 μl Cascade Blue-labeled knob (200 ng/μl), and 0.5 μl Alexa Fluor 488-labeled 4-12-1 (200 ng/μl). The cosmid and karyotyping probes were combined with 1.65 μl 2X SSC /1X TE per slide for a total of 5 μl probe mix added to each slide. After the first 20 F2 individuals were examined, 1 μl each of the 2.4- and 3.3-kb probes (200 ng/μl) were used on an additional 26 individuals, instead of cosmid 7 and 8 probes. A minimum of 10 chromosomes was observed from each individual root tip examined; a total of 8 chromosomes were observed for the B73, M825, and B73 x M825 hybrid. (A) Representative chromosomes for B73, M825, the B73 x M825 hybrid, and the first 20 F2 B73/M825 selfed chromosomes examined using cosmid 7 and 8 probes are shown. The chromosome to the left shows both the karyotyping probe (color) and mtDNA probe (white) layers while the chromosome on the right shows only the mtDNA probe layer. A white arrowhead indicates the position of the NUMT. Scale = 10 μm. (B) Both chromosomes 9 from a single cell are shown for individuals 15-20 to illustrate the similar probe signal strength present on both chromosomes. Three views of each homolog originating from a single cell are shown for all six individuals. The first view of the homologs (left) shows only the karyotyping probe layer (color), the second view of the homologs (middle) shows both the karyotyping probe (color) and mtDNA probe (white) layers, and the third view of the homologs (right) shows only the mtDNA probe layer (white). The white arrowheads indicate the presence of a 9L NUMT. Scale = 10 μm.

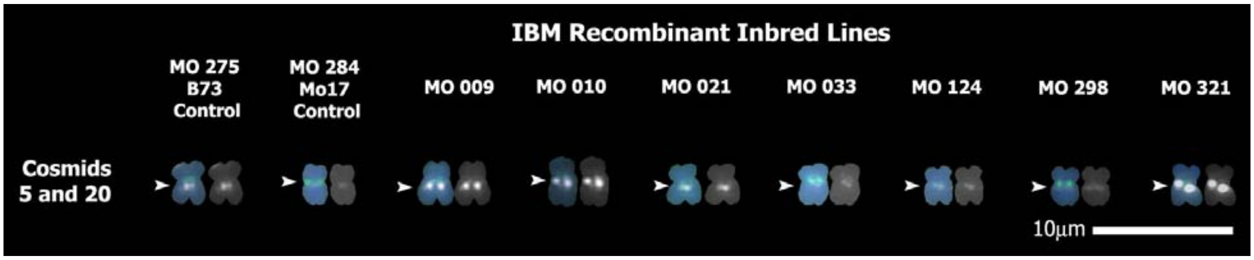

Figure S2 Test for recombination between the 9L NUMT in B73 and Mo17 using recombinant inbred lines derived from a B73 x Mo17 F1 hybrid. The Intermated B73 x Mo17 (IBM) recombinant inbred lines (RILs) are commonly used as a mapping tool in maize (Lee *et al*. 2002; Candela and Hake 2008). The IBM lines were created by first making hybrids of B73 and Mo17, self-pollinating the F1 plants for one generation, cross-pollinating their progeny for four generations, and finally self-pollinating the plants for multiple generations (Candela and Hake 2008). The resulting IBM RILs are homozygous at all loci and show alternating blocks of recombined parental DNA throughout the genome. Previous pachytene FISH experiments indicated that the B73 9L NUMT was located between the centromere and the BAC-specific and *glossy15* (*gl15*) probes (Figure S4). The closest IBM marker to *gl15* is umc1691 (244.10 cM). Data from K. Dawe's lab (personal communication) placed the chromosome 9 centromere below the IBM marker umc81 (226.30 cM). The range from 226.30 – 244.10 cM was extended to 219.40 – 251.80 cM (marker umc2338 to marker umc1700) to ensure that the strong signal 9L NUMT was included in the examined area. IBM RILs containing recombination between the markers umc2338 and umc1700 were selected. Initial concentrations are indicated for each probe with the volume used. Texas red-labeled cosmid 5 and 20 probes (1 μl each at 100 ng/μl) were used on every slide. These two cosmid probes were chosen because they are the only cosmid probes that were observed at the Mo17 9L NUMT (Figure 2B). To identify chromosome 9, three karyotyping probes were used per slide: 0.35 μl Alexa Fluor 488-labeled Cent C (50 ng/μl), 1 μl Cascade Blue-labeled knob (200 ng/μl), and 1 μl Alexa Fluor 488-labeled 4-12-1 (200 ng/μl). The cosmid and karyotyping probes were combined with 0.65 μl 2X SSC /1X TE per slide for a total of 5 μl probe mix added to each slide. A minimum of 17 chromosomes was observed from at least 2 individual root tips for each RIL examined. If the NUMTs are located at different sites on chromosome 9L, then recombination could occur and chromosomes containing no NUMT would be found. At this level of resolution, there is no evidence of the recombination event. Scale = 10 μm.

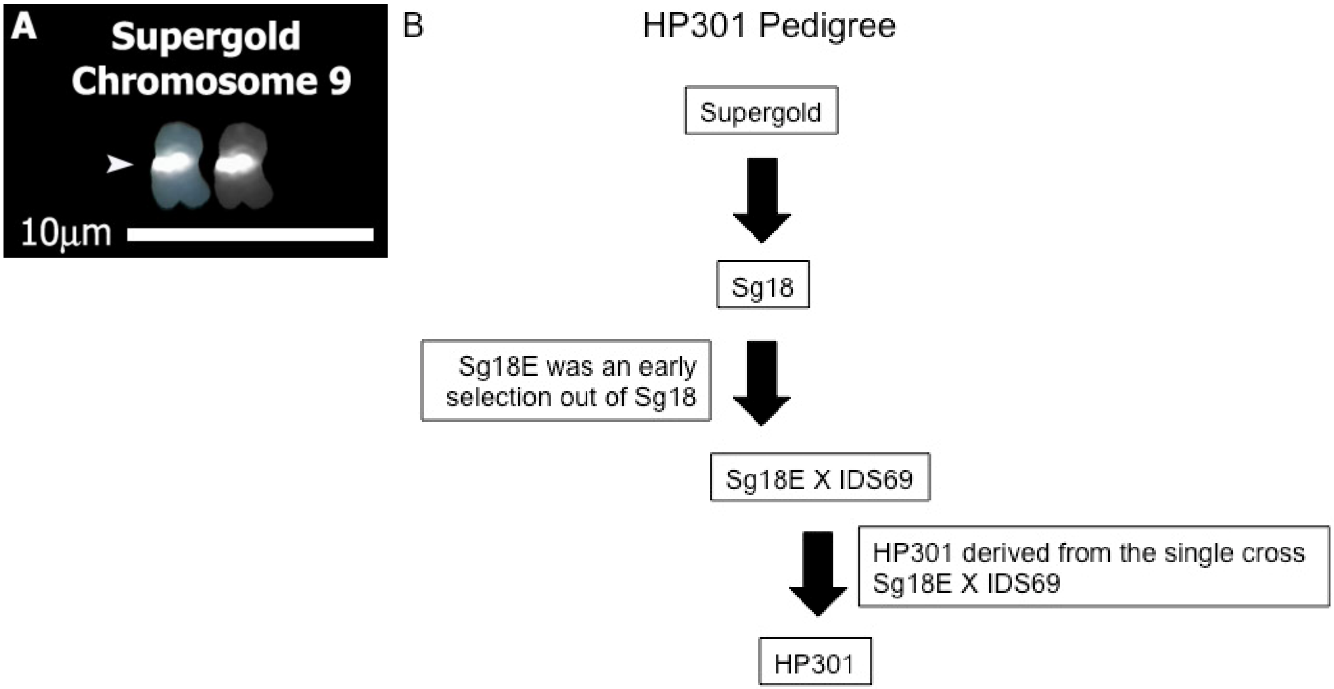

Figure S3 The 9L NUMT HP301 is present in the progenitor line Supergold. Supergold is a popcorn landrace that is a progenitor of HP301. Popcorns are a distinctive maize type in part because most popcorn lines contain a gametophytic incompatibility gene *Ga1*, which causes them to reject other types of pollen (Ziegler 2001; Lausser *et al*. 2010). (A) The Texas red-labeled 19-cosmid mix probe was used to examine Supergold and detected a relatively large NUMT on 9L near the centromere. This finding suggests that the HP301 9L NUMT was a descendent from the Supergold progenitor. Only chromosome 9 is shown. Sites of mtDNA hybridization are shown in white. Chromosomes were identified using a mix of eight karyotyping probes (shown in color). Chromosome on left: karyotyping probes and mtDNA probes. Chromosome on right: mtDNA probes only. White arrowhead indicates mtDNA insertions. Scale = 10 µm. (B) Pedigree of the popcorn line HP301. Listed here are the members of the HP301 pedigree, the popcorn line examined. Sg18E has not been maintained (M. Robbins and R.B. Ashman, personal communication). IDS69 is a South American line. The Supergold line examined in part (A) is one of the Supergold lines maintained by the Germplasm Resources Information Network (GRIN).

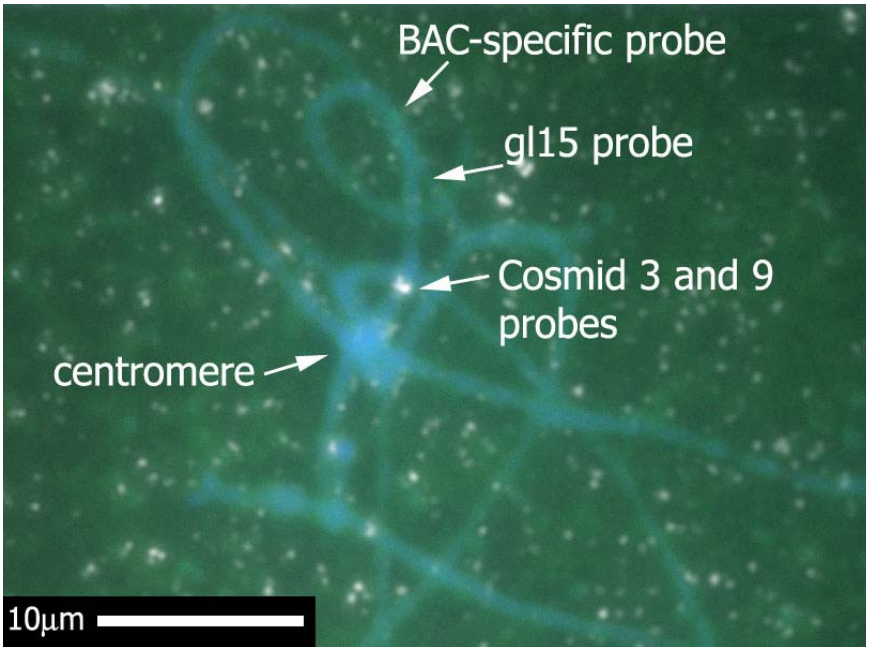

Figure S4 The B73 9L NUMT is located between the centromere and 9L-specific FISH probes on pachytene chromosomes. Chromosome 9 has been well-characterized cytogenetically with a series of probes on metaphase and pachytene chromosomes (Danilova and Birchler 2008). In the previous study, two proximal probes on the chromosome 9 long arm were BAC-specific probe (BAC-L1) and *glossy15* (*gl15*). B73 chromosomes were hybridized with Alexa Fluor 488-labeled BAC-specific and gl15 probes and Texas red-labeled cosmid 3 and 9 probes. Using these probes, the B73 chromosome 9L NUMT was identified between the 9L-specific probes and the centromere.

```
NUMT_1          CATGCTTGTGATTGGCATGTTCCGAGAAGAGCACGGCTAAGGGCAAAGCAGACAAGGCAC 60
NUMT_2          CATGCTTGTGATTGGCATGTTCCGAGAAGAGCACGGCTAAGGGCAAAGCAGACAAGGCAC 60
NA              CATGCTTGTGATTGGCATGTTCCGAGAAGAGCACGGCTAAGGGCAAAGCAGACAAGGCAC 60
Zmp             CATGCTTGTGATTGGCATGTTCCGAGAAGAGCACGGCTAAGGGCAAAGCAGACAAGGCAC 60
                ************************************************************

NUMT_1          GGGTCCTATCCGCGGATTCGCAATAGTTCTAGTTCCCCGAAAGGACATCGTTCGCTTGCG 120
NUMT_2          GGGTCCTATCCGCGGATTCGCAATAGTTCTAGTTCCCCGAAAGGACATCGTTCGCTTGCG 120
NA              GGGTCCTATCCGCGGATTCGCAATAGTTCTAGTTCCCCGAAAGGACATCGTTCGCTTGCG 120
Zmp             GGGTCCTATCCGCGGATTCGCAATAGTTCTAGTTCCCCGAAAGGACATCGTTCGCTTGCG 120
                ************************************************************

NUMT_1          GCTGAAGTGGAGATACGGCAGTAGTTCCTATCCGGTTTTCAACGGCTAAGGGTCGGTCCA 180
NUMT_2          GCTGAAGTGGAGATACGGCAGTAGTTCCTATCCGGTTTTCAACGGCTAAGGGTCGGTCCA 180
NA              GCTGAAGTGGAGATACGGCAGTAGTTCCTATCCGGTTTTCAACGGCTAAGGGTCGGTCCA 180
Zmp             GCTGAAGTGGAGATACGGCAGTAGTTCCTATCCGGTTTTCAACGGCTAAGGGTCGGTCCA 180
                ************************************************************

NUMT_1          TAGAACCCTCGGTTTCGGCTTTCCTAGTCGGCTTTTCCACGAGTTGGATCGTGATGATCG 240
NUMT_2          TAGAACCCTCGGTTTCGGCTTTCCTAGTCGGCTTTTCCACGAGTTGGATCGTGATGATCG 240
NA              TAGAACCCTCGGTTTCGGCTTTCCTAGTCGGCTTTTCCACGAGTTGGATCGTGATGATCG 240
Zmp             TAGAACCCTCGGTTTCGGCTTTCCTAGTCGGCTTTTCCACGAGTTGGATCGTGATGATCG 240
                ************************************************************

NUMT_1          GAACTGATTCCTTTCCTTCCTATCCAAATACCGGAAACGGCCTTCCTATTTGGAAAATCC 300
NUMT_2          GAACTGATTCCTTTCCTTCCTATCCAAATACCGGAAACGGCCTTCCTATTTGGAAAATCC 300
NA              GAACTGATTCCTTTCCTTCCTATCCAAATACCGGAAACGGCCTTCCTATTTGGAAAATCC 300
Zmp             GAACTGATTCCTTTCCTTCCTATCCAAATACCGGAAACGGCCTTCCTATTTGGAAAATCC 300
                ************************************************************

NUMT_1          TATCCTGGTTCTGTTTCTGTGTAAGCAACTATTGCCTTACTTGAGGATGAGTTTTCCCCT 360
NUMT_2          TATCCTGGTTCTGTTTCTGTGTAAGCAACTATTGCCTTACTTGAGGATGAGTTTTCCCCT 360
NA              TATCCTGGTTCTGTTTCTGTGTAAGCAACTATTGCCTTACTTGAGGATGAGTTTTCCCCT 360
Zmp             TATCCTGGTTCTGTTTCTGTGTAAGCAACTATTGCCTTACTTGAGGATGAGTTTTCCCCT 360
                ************************************************************

NUMT_1          TGCTTTGCAACAACCAAATTCCCTTTTCTTCCAAGTTTCCAAATTCGTACTTCTGCAAAG 420
NUMT_2          TGCTTTGCAACAACCAAATTCCCTTTTCTTCCAAGTTTCCAAATTCGTACTTCTGCAAAG 420
NA              TGCTTTGCAACAACCAAATTCCCTTTTCTTCCAAGTTTCCAAATTCGTACTTCTGCAAAG 420
Zmp             TGCTTTGCAACAACCAAATTCCCTTTTCTTCCAAGTTTCCAAATTCGTACTTCTGCAAAG 420
                ************************************************************

NUMT_1          CTTTAGGGGTAGGGGTTTAGAGAAAGGGTCTTTCTTTCTACCTATGACGAATAACTTCTT 480
NUMT_2          CTTTAGGGGTAGGGGTTTAGAGAAAGGGTCTTTCTTTCTACCTATGACGAATAACTTCTT 480
NA              CTTTAGGGGTAGGGGTTTAGAGAAAGGGTCTTTCTTTCTACCTATGACGAATAACTTCTT 480
Zmp             CTTTAGGGGTAGGGGTTTAGAGAAAGGGTCTTTCTTTCTACCTATGACGAATAACTTCTT 480
                ************************************************************

NUMT_1          ATTATCATGGCGACAGGCGTTGTAAACCTACTTTTCCTACCTCCAGATGAGGAGGCCTTA 540
NUMT_2          ATTATCATGGCGACAGGCGTTGTAAACCTACTTTTCCTACCTCCAGATGAGGAGGCCTTA 540
NA              ATTATCATGGCGACAGGCGTTGTAAACCTACTTTTCCTACCTCCAGATGAGGAGGCCTTA 540
Zmp             ATTATCATGGCGACAGGCGTTGTAAACCTACTTTTCCTACCTCCAGATGAGGAGGCCTTA 540
                ************************************************************

NUMT_1          GGACAAACATTCCGAAGTCACTGGAACGGCAGTGATGTCATCCTGTGTCAAAGTAGAGCA 600
NUMT_2          GGACAAACATTCCGAAGTCACTGGAACGGCAGTGATGTCATCCTGTGTCAAAGTAGAGCA 600
NA              GGACAAACATTCCGAAGTCACTGGAACGGCAGTGATGTCATCCTGTGTCAAAGTAGAGCA 600
Zmp             GGACAAACATTCCGAAGTCACTGGAACGGCAGTGATGTCATCCTGTGTCAAAGTAGAGCA 600
                ************************************************************

NUMT_1          GTAGCGATAGTGAATTACTATTCTCATTAACAATAGGGCCTTTCTTCCTCCAATTCGTTA 660
NUMT_2          GTAGCGATAGTGAATTACTATTCTCATTAACAATAGGGCCTTTCTTCCTCCAATTCGTTA 660
NA              GTAGCGATAGTGAATTACTATTCTCATTAACAATAGGGCCTTTCTTCCTCCAATTCGTTA 660
Zmp             GTAGCGATAGTGAATTACTATTCTCATTAACAATAGGGCCTTTCTTCCTCCAATTCGTTA 660
                ************************************************************

NUMT_1          CGTTTTCTGATTGCACTACTTCCTTCTAATCCAACAATTATAAACCTAATCCAGAATATA 720
NUMT_2          CGTTTTCTGATTGCACTACTTCCTTCTAATCCAACAATTATAAACCTAATCCAGAATATA 720
NA              CGTTTTCTGATTGCACTACTTCCTTCTAATCCAACAATTATAAACCTAATCCAGAATATA 720
Zmp             CGTTTTCTGATTGCACTACTTCCTTCTAATCCAACAATTATAAACCTAATCCAGAATATA 720
                ************************************************************
```

```
NUMT_1          AGAATCCATTAATGAATACGATCTTCCCGTTTATCTCCATCTGCAGGGCTAGGATTGATT 780
NUMT_2          AGAATCCATTAATGAATACGATCTTCCCGTTTATCTCCATCTGCAGGGCTAGGATTGATT 780
NA              AGAATCCATTAATGAATACGATCTTCCCGTTTATCTCCATCTGCAGGGCTAGGATTGATT 780
Zmp             AGAATCCATTAATGAATACGATCTTCCCGTTTATCTCCATCTGCAGGGCTAGGATTGATT 780
                ************************************************************

NUMT_1          TCTTCTTCTCGATAGTGATGAGGCTAGTGCTCCTTCTCTTCTTCCCAATTCCTGGAAGCC 840
NUMT_2          TCTTCTTCTCGATAGTGATGAGGCTAGTGCTCCTTCTCTTCTTCCCAATTCCTGGAAGCC 840
NA              TCTTCTTCTCGATAGTGATGAGGCTAGTGCTCCTTCTCTTCTTCCCAATTCCTGGAAGCC 840
Zmp             TCTTCTTCTCGATAGTGATGAGGCTAGTGCTCCTTCTCTTCTTCCCAATTCCTGGAAGCC 840
                ************************************************************

NUMT_1          TTCTACTCCTCGAAACCCTGTCTTTTGTTTGATTTAGTTACAGATGGACTATCAAAGATA 900
NUMT_2          TTCTACTCCTCGAAACCCTGTCTTTTGTTTGATTTAGTTACAGATGGACTATCAAAGATA 900
NA              TTCTACTCCTCGAAACCCTGTCTTTTGTTTGATTTAGTTACAGATGGACTATCAAAGATA 900
Zmp             TTCTACTCCTCGAAACCCTGTCTTTTGTTTGATTTAGTTACAGATGGACTATCAAAGATA 900
                ************************************************************

NUMT_1          TTGGCTAGAGGTCAAGATGTTGGGTGCCATGGATCGAGTGATAAAATAAAATCAGAATTG 960
NUMT_2          TTGGCTAGAGGTCAAGATGTTGGGTGCCATGGATCGAGTGATAAAATAAAATCAGAATTG 960
NA              TTGGCTAGAGGTCAAGATG TGGGTGCCATGGATCGAGTGATAAAATAAAATCAGAATTG 960
Zmp             TTGGCTAGAGGTCAAGATG TGGGTGCCATGGATCGAGTGATAAAATAAAATCAGAATTG 960
                ******************* ****************************************

NUMT_1          TTTTCAAAGTGAAATGTTGCTCCTCAGAAAACGCGTATAGTAATCTCATTGGCCTTCGTC 1020
NUMT_2          TTTTCAAAGTGAAATGTTGCTCCTCAGAAAACGCGTATAGTAATCTCATTGGCCTTCGTC 1020
NA              TTTTCAAAGTGAAATGTTGCTCCTCAGAAAACGCGTATAGTAATCTCATTGGCCTTCGTC 1020
Zmp             TTTTCAAAGTGAAATGTTGCTCCTCAGAAAACGCGTATAGTAATCTCATTGGCCTTCGTC 1020
                ************************************************************

NUMT_1          GATGGGACAAATGCTCCAGATGAGTCCTTTTGCTCAAAAGAGAGAAGGGACAGGAATCTA 1080
NUMT_2          GATGGGACAAATGCTCCAGATGAGTCCTTTTGCTCAAAAGAGAGAAGGGACAGGAATCTA 1080
NA              GATGGGACAAATGCTCCAGATGAGTCCTTTTGCTCAAAAGAGAGAAGGGACAGGAATCTA 1080
Zmp             GATGGGACAAATGCTCCAGATGAGTCCTTTTGCTCAAAAGAGAGAAGGGACAGGAATCTA 1080
                ************************************************************

NUMT_1          TTCTATAAGTTGTAAAAAGATGTGCGTATACTGGTCGACGTCATGTGATCGCTACTAAAG 1140
NUMT_2          TTCTATAAGTTGTAAAAAGATGTGCGTATACTGGTCGACGTCATGTGATCGCTACTAAAG 1140
NA              TTCTATAAGTTGTAAAAAGATGTGCGTATACTGGTCGACGTCATGTGATCGCTACTAAAG 1140
Zmp             TTCTATAAGTTGTAAAAAGATGTGCGTATACTGGTCGACGTCATGTGATCGCTACTAAAG 1140
                ************************************************************

NUMT_1          ATAGAATTTCCTTTCCTTGGAAAAACCAAGGCCAGTTGAGAGAAGTCTTTCTGCTTAGAG 1200
NUMT_2          ATAGAATTTCCTTTCCTTGGAAAAACCAAGGCCAGTTGAGAGAAGTCTTTCTGCTTAGAG 1200
NA              ATAGAATTTCCTTTCCTTGGAAAAACCAAGGCCAGTTGAGAGAAGTCTTTCTGCTTAGAG 1200
Zmp             ATAGAATTTCCTTTCCTTGGAAAAACCAAGGCCAGTTGAGAGAAGTCTTTCTGCTTAGAG 1200
                ************************************************************

NUMT_1          CAAGAAGCGGAACCCAAATCAAGCTTTCTTTATTTTCATTTATGGATAACCAATTCATTG 1260
NUMT_2          CAAGAAGCGGAACCCAAATCAAGCTTTCTTTATTTTCATTTATGGATAACCAATTCATTG 1260
NA              CAAGAAGCGGAACCCAAATCAAGCTTTCTTTATTTTCATTTATGGATAACCAATTCATTG 1260
Zmp             CAAGAAGCGGAACCCAAATCAAGCTTTCTTTATTTTCATTTATGGATAACCAATTCATTG 1260
                ************************************************************

NUMT_1          ATTATGTAGGCATCGGAATCATTGTTTCCGTTGCCATTTTTTTTGCTTATAAAGCGGGGC 1320
NUMT_2          ATTATGTAGGCATCGGAATCATTGTTTCCGTTGCCATTTTTTTTGCTTATAAAGCGGGGC 1320
NA              ATTATGTAGGCATCGGAATCATTGTTTCCGTTGCCATTTTTTT GCTTATAAAGCGGGGC 1320
Zmp             ATTATGTAGGCATCGGAATCATTGTTTCCGTTGCCATTTTTTT GCTTATAAAGCGGGGC 1320
                ******************************************* ****************

NUMT_1          AGCTGGCTGAACGAATCCATTCCCACACATTGGAAATGGAAATACTCAAACAAAAGCTGG 1380
NUMT_2          AGCTGGCTGAACGAATCCATTCCCACACATTGGAAATGGAAATACTCAAACAAAAGCTGG 1380
NA              AGCTGGCTGAACGAATCCATTCCCACACATTGGAAATGGAAATACTCAAACAAAAGCTGG 1380
Zmp             AGCTGGCTGAACGAATCCATTCCCACACATTGGAAATGGAAATACTCAAACAAAAGCTGG 1380
                ************************************************************

NUMT_1          AATATAAGCTAAAAATGCTTTTGGAGCAAACTTCTGGTAATGCGCAATTGCCGGAGGGAT 1440
NUMT_2          AATATAAGCTAAAAATGCTTTTGGAGCAAACTTCTGGTAATGCGCAATTGCCGGAGGGAT 1440
NA              AATATAAGCTAAAAATGCTTTTGGAGCAAACTTCTGGTAATGCGCAATTGCCGGAGGGAT 1440
Zmp             AATATAAGCTAAAAATGCTTTTGGAGCAAACTTCTGGTAATGCGCAATTGCCGGAGGGAT 1440
                ************************************************************
```

```
NUMT_1          TCTCACTCCGGGATATCATTACAAATATGGTTTTCTCGGGGGACTCTATAGAAGAACAGC 1500
NUMT_2          TCTCACTCCGGGATATCATTACAAATATGGTTTTCTCGGGGGACTCTATAGAAGAACAGC 1500
NA              TCTCACTCCGGGATATCATTACAAATATGGTTTTCTCGGGGGACTCTATAGAAGAACAGC 1500
Zmp             TCTCACTCCGGGATATCATTACAAATATGGTTTTCTCGGGGGACTCTATAGAAGAACAGC 1500
                ************************************************************

NUMT_1          TTCTAGCATTAAATCGGATCTATCTTGATCTGGTCGTTCATGGACACAGTAGTGACTACT 1560
NUMT_2          TTCTAGCATTAAATCGGATCTATCTTGATCTGGTCGTTCATGGACACAGTAGTGACTACT 1560
NA              TTCTAGCATTAAATCGGATCTATCTTGATCTGGTCGTTCATGGACACAGTAGTGACTACT 1560
Zmp             TTCTAGCATTAAATCGGATCTATCTTGATCTGGTCGTTCATGGACACAGTAGTGACTACT 1560
                ************************************************************

NUMT_1          TTTTAATGATTCTCAATTAATTATTTTAATTAGCAGTTGCTGTAGGCAACTAGCATTTTG 1620
NUMT_2          TTTTAATGATTCTCAATTAATTATTTTAATTAGCAGTTGCTGTAGGCAACTAGCATTTTG 1620
NA              TTTTAATGATTCTCAATTAATTATTTTAATTAGCAGTTGCTGTAGGCAACTAGCATTTTG 1620
Zmp             TTTTAATGATTCTCAATTAATTATTTTAATTAGCAGTTGCTGTAGGCAACTAGCATTTTG 1620
                ************************************************************

NUMT_1          TTTTGTCATGGAATCAAGTCTATTTGTTCTTTTTTGCGTTTCGTTGGAAAAACCCACGCC 1680
NUMT_2          TTTTGTCATGGAATCAAGTCTATTTGTTCTTTTTTGCGTTTCGTTGGAAAAACCCACGCC 1680
NA              TTTTGTCATGGAATCAAGTCTATTTGTTCTTTTTTGCGTTTCGTTGGAAAAACCCACGCC 1680
Zmp             TTTTGTCATGGAATCAAGTCTATTTGTTCTTTTTT CGTTTCGTTGGAAAAACCCACGCC 1680
                *********************************** ************************

NUMT_1          AACCAAAATCCAAGTCTCCCTTTCTCTTTTGGGAGCAGATTGTATTGTATTTTATAAAGT 1740
NUMT_2          AACCAAAATCCAAGTCTCCCTTTCTCTTTTGGGAGCAGATTGTATTGTATTTTATAAAGT 1740
NA              AACCAAAATCCAAGTCTCCCTTTCTCTTTTGGGAGCAGATTGTATTGTATTTTATAAAGT 1740
Zmp             AACCAAAATCCAAGTCTCCCTTTCTCTTTTGGGAGCAGATTGTATTGTATTTTATAAAGT 1740
                ************************************************************

NUMT_1          TGAGAGTCACAATGAGATTTAGTGGAATGGATATGAAGGGTATAAATATGGTATTTGCTG 1800
NUMT_2          TGAGAGTCACAATGAGATTTAGTGGAATGGATATGAAGGGTATAAATATGGTATTTGCTG 1800
NA              TGAGAGTCACAATGAGATTTAGTGGAATGGATATGAAGGGTATAAATATGGTATTTGCTG 1800
Zmp             TGAGAGTCACAATGAGATTTAGTGGAATGGATATGAAGGGTATAAATATGGTATTTGCTG 1800
                ************************************************************

NUMT_1          CTATTCCGAAAGCTATGAAACAAGTCTGGGGAAACAGAAATTGGCACAATATCGCGAAGT 1860
NUMT_2          CTATTCCGAAAGCTATGAAACAAGTCTGGGGAAACAGAAATTGGCACAATATCGCGAAGT 1860
NA              CTATTCCGAAAGCTATGAAACAAGTCTGGGGAAACAGAAATTGGCACAATATCGCGAAGT 1860
Zmp             CTATTCCGAAAGCTATGAAACAAGTCTGGGGAAACAGAAATTGGCACAATATCGCGAAGT 1860
                ************************************************************

NUMT_1          GGCTGCCTTCGCTCAATTGTGCCTAACTTGGGCCATAAAGATTTTCGCTAATAACGGGAG 1920
NUMT_2          GGCTGCCTTCGCTCAATTGTGCCTAACTTGGGCCATAAAGATTTTCGCTAATAACGGGAG 1920
NA              GGCTGCCTTCGCTCAATTGTGCCTAACTTGGGCCATAAAGATTTTCGCTAATAACGGGAG 1920
Zmp             GGCTGCCTTCGCTCAATTGTGCCTAACTTGGGCCATAAAGATTTTCGCTAATAACGGGAG 1920
                ************************************************************

NUMT_1          TGGGAGTGCTCTCCTTCTTTCCTTCTGTTTTTTTTCTTTCTTCTTCTTTGCAACGACAAG 1980
NUMT_2          TGGGAGTGCTCTCCTTCTTTCCTTCTGTTTTTTTTCTTTCTTCTTCTTTGCAACGACAAG 1980
NA              TGGGAGTGCTCTCCTTCTTTCCTTCTGTTTTTTTTCTTTCTTCTTCTTTGCAACGACAAG 1980
Zmp             TGGGAGTGCTCTCCTTCTTTCCTTCTGTTTTTTTTCTTTCTTCTTCTTTGCAACGACAAG 1980
                ************************************************************

NUMT_1          GGCGGAGGCTGCAGGCCCATCGGATGGCCCATCGGATTGGATGAAAGGGCACCCGGATGA 2040
NUMT_2          GGCGGAGGCTGCAGGCCCATCGGATGGCCCATCGGATTGGATGAAAGGGCACCCGGATGA 2040
NA              GGCGGAGGCTGCAGGCCCATCGGATGGCCCATCGGATTGGATGAAAGGGCACCCGGATGA 2040
Zmp             GGCGGAGGCTGCAGGCCCATCGGATGGCCCATCGGATTGGATGAAAGGGCACCCGGATGA 2040
                ************************************************************

NUMT_1          AACCTTGCTTCGTAAAACGGAGAAGGAGATTCTACGGGTGCAAGAGGAGGTTGATACCCT 2100
NUMT_2          AACCTTGCTTCGTAAAACGGAGAAGGAGATTCTACGGGTGCAAGAGGAGGTTGATACCCT 2100
NA              AACCTTGCTTCGTAAAACGGAGAAGGAGATTCTACGGGTGCAAGAGGAGGTTGATACCCT 2100
Zmp             AACCTTGCTTCGTAAAACGGAGAAGGAGATTCTACGGGTGCAAGAGGAGGTTGATACCCT 2100
                ************************************************************

NUMT_1          CGCAACCAAAGCAGTAGAAAAAGGTCACTTTTATGGGCTAGGTCTGCCCGGTACCCCGGC 2160
NUMT_2          CGCAACCAAAGCAGTAGAAAAAGGTCACTTTTATGGGCTAGGTCTGCCCGGTACCCCGGC 2160
NA              CGCAACCAAAGCAGTAGAAAAAGGTCACTTTTATGGGCTAGGTCTGCCCGGTACCCCGGC 2160
Zmp             CGCAACCAAAGCAGTAGAAAAAGGTCACTTTTATGGGCTAGGTCTGCCCGGTACCCCGGC 2160
                ************************************************************
```

```
NUMT_1          GGAGCAGAAGGATACTATAAAATCTATTATCGACCTTGATCTAGATAGTATAGATCTGGA 2220
NUMT_2          GGAGCAGAAGGATACTATAAAATCTATTATCGACCTTGATCTAGATAGTATAGATCTGGA 2220
NA              GGAGCAGAAGGATACTATAAAATCTATTATCGACCTTGATCTAGATAGTATAGATCTGGA 2220
Zmp             GGAGCAGAAGGATACTATAAAATCTATTATCGACCTTGATCTAGATAGTATAGATCTGGA 2220
                ************************************************************

NUMT_1          TAAGCGGTCGAAAAGACTAAAAAGTTGGCTCAACAGCGGGGTAGATAACCCGGATAGCTC 2280
NUMT_2          TAAGCGGTCGAAAAGACTAAAAAGTTGGCTCAACAGCGGGGTAGATAACCCGGATAGCTC 2280
NA              TAAGCGGTCGAAAAGACTAAAAAGTTGGCTCAACAGCGGGGTAGATAACCCGGATAGCTC 2280
Zmp             TAAGCGGTCGAAAAGACTAAAAAGTTGGCTCAACAGCGGGGTAGATAACCCGGATAGCTC 2280
                ************************************************************

NUMT_1          TTTTTGGTTGATGCTCGTACACGAGATCACAAAATGGTATCCATAGGTTTTATTTGCATG 2340
NUMT_2          TTTTTGGTTGATGCTCGTACACGAGATCACAAAATGGTATCCATAGGTTTTATTTGCATG 2340
NA              TTTTTGGTTGATGCTCGTACACGAGATCACAAAATGGTATCCATAGGTTTTATTTGCATG 2340
Zmp             TTTTTGGTTGATGCTCGTACACGAGATCACAAAATGGTATCCATAGGTTTTATTTGCATG 2340
                ************************************************************

NUMT_1          TTAGAAAGGAAAGGAAAGGAGATAGAGGTGCAAGGCTTACAGAAGTGCCCAAAGTTTCA 2399
NUMT_2          TTAGAAAGGAAAGGAAAGGAGATAGAGGTGCAAGGCTTACAGAAGTGCCCAAAGTTTCA 2399
NA              TTAGAAAGGAAAGGAAAGGAGATAGAGGTGCAAGGCTTACAGAAGTGCCCAAAGTTTCA 2399
Zmp             TTAGAAAGGAAAGGAAAGGAGATAGAGGTGCAAGGCTTACAGAAGTGCCCAAAGTTTCA 2399
                ***********************************************************
```

Figure S5 Multiple sequence alignment of the two B73 2.4-kb NUMT regions with the corresponding NA and Zmp mitochondrial genome regions. The 2.4-kb NUMT region is present within both the NA (NCBI Accession DQ490952.1) and Zmp (NCBI Accession DQ645539.1) mitochondrial genomes, but has fewer nucleotide differences when compared to the NA genome. The sequences were aligned with ClustalW2 (McWilliam *et al.* 2013). The reverse complement of the second 2.4-kb region in the NUMT was used in this alignment.

```
NUMT_1          TATTACAATAGACGTTGAAACAACCCTGGAGCTTATCTGTAATTTGCTCCTTGAGCGTTT 60
NUMT_3          TATTACAATAGACGTTGAAACAACCCTGGAGCTTATCTGTAATTTGCTCCTTGAGCGTTT 60
NA              TATTACAATAGACGTTGAAACAACCCTGGAGCTTATCTGTAATTTGCTCCTTGAGCGTTT 60
Zmp             TATTACAATAGACGTTGAAACAACCCTGGAGCTTATCTGTAATTTGCTCCTTGAGCGTTT 60
CMS-S           TATTACAATAGACGTTGAAACAACCCTGGAGCTTATCTGTAATTTGCTCCTTGAGCGTTT 60
NUMT_2          TATTACAATAGACGTTGAAACAACCCTGGAGCTTATCTGTAATTTGCTCCTTGAGCGTTT 60
CMS-T           TATTACAATAGACGTTGAAACAACCCTGGAGCTTATCTGTAATTTGCTCCTTGAGCGTTT 60
                ************************************************************

NUMT_1          CTAACGTCAATAAA-----GTCCTCCAACTTATGATGCCAGTTTTCCGAAGCCGCGGCTT 115
NUMT_3          CTAACGTCAATAAA-----GTCCTCCAACTTATGATGCCAGTTTTCCGAAGCCGCGGCTT 115
NA              CTAACGTCAATAAA-----GTCCTCCAACTTATGATGCCAGTTTTCCGAAGCCGCGGCTT 115
Zmp             CTAACGTCAATAAA-----GTCCTCCAACTTATGATGCCAGTTTTCCGAAGCCGCGGCTT 115
CMS-S           CTAACGTCAATAAA-----GTCCTCCAACTTATGATGCCAGTTTTCCGAAGCCGCGGCTT 115
NUMT_2          CTAACGTCAATAAA-----GTCCTCCAACTTATGATGCCAGTTTTCCGAAGCCGCGGCTT 115
CMS-T           CTAACGTCAATAAA     GTCCTCCAACTTATGATGCCAGTTTTCCGAAGCCGCGGCTT 120
                **************     *****************************************

NUMT_1          TTACCCGCTTTATAAGCGATGAGTAGGGCGATGCATAAAAAGTCATATTTCTTGGTGTAG 175
NUMT_3          TTACCCGCTTTATAAGCGATGAGTAGGGCGATGCATAAAAAGTCATATTTCTTGGTGTAG 175
NA              TTACCCGCTTTATAAGCGATGAGTAGGGCGATGCATAAAAAGTCATATTTCTTGGTGTAG 175
Zmp             TTACCCGCTTTATAAGCGATGAGTAGGGCGATGCATAAAAAGTCATATTTCTTGGTGTAG 175
CMS-S           TTACCCGCTTTATAAGCGATGAGTAGGGCGATGCATAAAAAGTCATATTTCTTGGTGTAG 175
NUMT_2          TTACCCGCTTTATAAGCGATGAGTAGGGCGATGCATAAAAAGTCATATTTCTTGGTGTAG 175
CMS-T           TTACCCGCTTTATAAGCGATGAGTAGGGCGATGCATAAAAAGTCATATTTCTTGGTGTAG 180
                ************************************************************

NUMT_1          GGATGGATCTCATAGGAAAAGAGATACCGAGGCCCACCAACCGTATACTTGATTTATGGT 235
NUMT_3          GGATGGATCTCATAGGAAAAGAGATACCGAGGCCCACCAACCGTATACTTGATTTATGGT 235
NA              GGATGGATCTCATAGGAAAAGAGATACCGAGGCCCACCAACCGTATACTTGATTTATGGT 235
Zmp             GGATGGATCTCATAGGAAAAGAGATACCGAGGCCCACCAACCGTATACTTGATTTATGGT 235
CMS-S           GGATGGATCTCATAGGAAAAGAGATACCGAGGCCCACCAACCGTATACTTGATTTATGGT 235
NUMT_2          GGATGGATCTCATAGGAAAAGAGATACCGAGGCCCACCAACC TATACTTGATTTATGGT 235
CMS-T           GGA    TCTCATAGGAAAAGAGATACCGAGGCCCACCAACCGTATACTTGATTTATGGT 236
                ***    *********************************** *****************

NUMT_1          TTGGTGGGGAAAGAAGAGTGGGTATGGGGCTTCTTTCATGGTGCCATTCTTTACTTTACG 295
NUMT_3          TTGGTGGGGAAAGAAGAGTGGGTATGGGGCTTCTTTCATGGTGCCATTCTTTACTTTACG 295
NA              TTGGTGGGGAAAGAAGAGTGGGTATGGGGCTTCTTTCATGGTGCCATTCTTTACTTTACG 295
Zmp             TTGGTGGGGAAAGAAGAGTGGGTATGGGGCTTCTTTCATGGTGCCATTCTTTACTTTACG 295
CMS-S           TTGGTGGGGAAAGAAGAGTGGGTATGGGGCTTCTTTCATGGTGCCATTCTTTACTTTACG 295
NUMT_2          TTGGTGGGGAAAGAAGAGTGGGTATGGGGCTTCTTTCATGGTGCCATTCTTTACTTTACG 295
CMS-T           TTGGTGGGGAAAGAAGAGTGGGTATGGGGCTTCTTTCATGGTGCCATTCTTTACTTTACG 296
                ************************************************************

NUMT_1          TAATAAAAATCAGAGAGGGACTGAACACTTGTTTTGATCTACGAAGAGTTGAAAAACAAT 355
NUMT_3          TAATAAAAATCAGAGAGGGACTGAACACTTGTTTTGATCTACGAAGAGTTGAAAAACAAT 355
NA              TAATAAAAATCAGAGAGGGACTGAACACTTGTTTTGATCTACGAAGAGTTGAAAAACAAT 355
Zmp             TAATAAAAATCAGAGAGGGACTGAACACTTGTTTTGATCTACGAAGAGTTGAAAAACAAT 355
CMS-S           TAATAAAAATCAGAGAGGGACTGAACACTTGTTTTGATCTACGAAGAGTTGAAAAACAAT 355
NUMT_2          TAATAAAAATCAGAGAGGGACTGAACACTTGTTTTGATCTACGAAGAGTTGAAAAACAAT 355
CMS-T           TAATAAAAATCAGAGAGGGACTGAACACTTGTTTTGATCTACGAAGAGTTGAAAAACAAT 356
                ************************************************************

NUMT_1          TGAATTGCCTTTATTTGATCTTAAAGAATCGGCATTGGCTTCAGTTCAGATCTTATGGGA 415
NUMT_3          TGAATTGCCTTTATTTGATCTTAAAGAATCGGCATTGGCTTCAGTTCAGATCTTATGGGA 415
NA              TGAATTGCCTTTATTTGATCTTAAAGAATCGGCATTGGCTTCAGTTCAGATCTTATGGGA 415
Zmp             TGAATTGCCTTTATTTGATCTTAAAGAATCGGCATTGGCTTCAGTTCAGATCTTATGGGA 415
CMS-S           TGAATTGCCTTTATTTGATCTTAAAGAATCGGCATTGGCTTCAGTTCAGATCTTATGGGA 415
NUMT_2          TGAATTGCCTTTATTTGATCTTAAAGAATCGGCATTGGCTTCAGTTCAGATCTTATGGGA 415
CMS-T           TGAATTGCCTTTATTTGATCTTAAAGAATCGGCATTGGCTTCAGTTCAGATCTTATGGGA 416
                ************************************************************

NUMT_1          AAAGGCGCGTAGCGAAGAAGTGTATGCTCAATAAACTGAAGAAGCATATATAAGAAAGAA 475
NUMT_3          AAAGGCGCGTAGCGAAGAAGTGTATGCTCAATAAACTGAAGAAGCATATATAAGAAAGAA 475
NA              AAAGGCGCGTAGCGAAGAAGTGTATGCTCAATAAACTGAAGAAGCATATATAAGAAAGAA 475
Zmp             AAAGGCGCGTAGCGAAGAAGTGTATGCTCAATAAACTGAAGAAGCATATATAAGAAAGAA 475
CMS-S           AAAGGCGCGTAGCGAAGAAGTGTATGCTCAATAAACTGAAGAAGCATATATAAGAAAGAA 475
NUMT_2          AAAGGCGCGTAGCGAAGAAGTGTATGCTCAATAAACTGAAGAAGCATATATAAGAAAGAA 475
CMS-T           AAAGGCGCGTAGCGAAGAAGTGTATGCTCAATAAACTGAAGAAGCATATATAAGAAAGAA 476
                ************************************************************
```

```
NUMT_1          GCCATCATCACTATGTTTACTCCTACCCAAGGAAGGAGGTCAGTCAAAGCAATGAAATGA 535
NUMT_3          GCCATCATCACTATGTTTACTCCTACCCAAGGAAGGAGGTCAGTCAAAGCAATGAAATGA 535
NA              GCCATCATCACTATGTTTACTCCTACCCAAGGAAGGAGGTCAGTCAAAGCAATGAAATGA 535
Zmp             GCCATCATCACTATGTTTACTCCTACCCAAGGAAGGAGGTCAGTCAAAGCAATGAAATGA 535
CMS-S           GCCATCATCACTATGTTTACTCCTACCCAAGGAAGGAGGTCAGTCAAAGCAATGAAATGA 535
NUMT_2          GCCATCATCACTATGTTTACTCCTACCCAAGGAAGGAGGTCAGTCAAAGCAATGAAATGA 535
CMS-T           GCCATCATCACTATGTTTACTCCTACCCAAGGAAGGAGGTCAGTCAAAGCAATGAAATGA 536
                ************************************************************

NUMT_1          AGAGGCCGTGACCTAATACCTAT TGTGTTCTCTTGGATAGTGGTCCAGTTTGAATAGTT 595
NUMT_3          AGAGGCCGTGACCTAATACCTAT TGTGTTCTCTTGGATAGTGGTCCAGTTTGAATAGTT 595
NA              AGAGGCCGTGACCTAATACCTATCTGTGTTCTCTTGGATAGTGGTCCAGTTTGAATAGTT 595
Zmp             AGAGGCCGTGACCTAATACCTATCTGTGTTCTCTTGGATAGTGGTCCAGTTTGAATAGTT 595
CMS-S           AGAGGCCGTGACCTAATACCTATCTGTGTTCTCTTGGATAGTGGTCCAGTTTGAATAGTT 595
NUMT_2          AGAGGC GTGACCTAATACCTATCTGTGTTCTCTTGGATAGTGGTCCAGTTTGAATAGTT 595
CMS-T           AGAGGCCGTGACCTAATACCTATCTGTGTTCTCTTGGATAGTGGTCCAGTTTGAATAGTT 596
                ****** **************** ************************************

NUMT_1          GTATAGTTATTTGTAAACCCGGGGGCCCTGAAATTAGCCAAAACAACCCGGTGGGGTAAA 655
NUMT_3          GTATAGTTATTTGTAAACCCGGGGGCCCTGAAATTAGCCAAAACAACCCGGTGGGGTAAA 655
NA              GTATAGTTATTTGTAAACCCGGGGGCCCTGAAATTAGCCAAAACAACCCGGTGGGGTAAA 655
Zmp             GTATAGTTATTTGTAAACCCGGGGGCCCTGAAATTAGCCAAAACAACCCGGTGGGGTAAA 655
CMS-S           GTATAGTTATTTGTAAACCCGGGGGCCCTGAAATTAGCCAAAACAACCCGGTGGGGTAAA 655
NUMT_2          GTATAGTTATTTGTAAACCCGGGGGCCCTGAAATTAGCCAAAACAACCCGGTGGGGTAAA 655
CMS-T           GTATAGTTATTTGTAAACCCGGGGGCCCTGAAATTAGCCAAAACAACCCGGTGGGGTAAA 656
                ************************************************************

NUMT_1          GTCGTCAAGTGGACTATGGTTCACAATAATAGTGACTGACACGAGATGCGATGCCAAGTT 715
NUMT_3          GTCGTCAAGTGGACTATGGTTCACAATAATAGTGACTGACACGAGATGCGATGCCAAGTT 715
NA              GTCGTCAAGTGGACTATGGTTCACAATAATAGTGACTGACACGAGATGCGATGCCAAGTT 715
Zmp             GTCGTCAAGTGGACTATGGTTCACAATAATAGTGACTGACACGAGATGCGATGCCAAGTT 715
CMS-S           GTCGTCAAGTGGACTATGGTTCACAATAATAGTGACTGACACGAGATGCGATGCCAAGTT 715
NUMT_2          GTCGTCAAGTGGACTATGGTTCACAATAATAGTGACTGACACGAGATGCGATGCCAAGTT 715
CMS-T           GTCGTCAAGTGGACTATGGTTCACAATAATAGTGACTGACACGAGATGCGATGCCAAGTT 716
                ************************************************************

NUMT_1          AGAAGGTCAAAAGTGAGAAAGTTGGAGGGGAGATGCCATGATCCTAGGTGTAGATTGGCT 775
NUMT_3          AGAAGGTCAAAAGTGAGAAAGTTGGAGGGGAGATGCCATGATCCTAGGTGTAGATTGGCT 775
NA              AGAAGGTCAAAAGTGAGAAAGTTGGAGGGGAGATGCCATGATCCTAGGTGTAGATTGGCT 775
Zmp             AGAAGGTCAAAAGTGAGAAAGTTGGAGGGGAGATGCCATGATCCTAGGTGTAGATTGGCT 775
CMS-S           AGAAGGTCAAAAGTGAGAAAGTTGGAGGGGAGATGCCATGATCCTAGGTGTAGATTGGCT 775
NUMT_2          AGAAGGTCAAAAGTGAGAAAGTTGGAGGGGAGATGCCATGATCCTAGGTGTAGATTGGCT 775
CMS-T           AGAAGGTCAAAAGTGAGAAAGTTGGAGGGGAGATGCCATGATCCTAGGTGTAGATTGGCT 776
                ************************************************************

NUMT_1          TATCAGCCTATGGCCATTCCTTTAATACTTTTGAACGATGACTTAATGCTTCAAAGCCCT 835
NUMT_3          TATCAGCCTATGGCCATTCCTTTAATACTTTTGAACGATGACTTAATGCTTCAAAGCCCT 835
NA              TATCAGCCTATGGCCATTCCTTTAATACTTTTGAACGATGACTTAATGCTTCAAAGCCCT 835
Zmp             TATCAGCCTATGGCCATTCCTTTAATACTTTTGAACGATGACTTAATGCTTCAAAGCCCT 835
CMS-S           TATCAGCCTATGGCCATTCCTTTAATACTTTTGAACGATGACTTAATGCTTCAAAGCCCT 835
NUMT_2          TATCAGCCTATGGCCATTCCTTTAATACTTTTGAACGATGACTTAATGCTTCAAAGCCCT 835
CMS-T           TATCAGCCTATGGCCATTCCTTTAATACTTTTGAACGATGACTTAATGCTTCAAAGCCCT 836
                ************************************************************

NUMT_1          CCCCATATAGCACAGCCTTCGTAAGGCTTATCACACTCGGGCCTGTAACTGGAATCTGAG 895
NUMT_3          CCCCATATAGCACAGCCTTCGTAAGGCTTATCACACTCGGGCCTGTAACTGGAATCTGAG 895
NA              CCCCATATAGCACAGCCTTCGTAAGGCTTATCACACTCGGGCCTGTAACTGGAATCTGAG 895
Zmp             CCCCATATAGCACAGCCTTCGTAAGGCTTATCACACTCGGGCCTGTAACTGGAATCTGAG 895
CMS-S           CCCCATATAGCACAGCCTTCGTAAGGCTTATCACACTCGGGCCTGTAACTGGAATCTGAG 895
NUMT_2          CCCCATATAGCACAGCCTTCGTAAGGCTTATCACACTCGGGCCTGTAACTGGAATCTGAG 895
CMS-T           CCCCATATAGCACAGCCTTCGTAAGGCTTATCACACTCGGGCCTGTAACTGGAATCTGAG 896
                ************************************************************

NUMT_1          CATTTCTTGTTTGGGGTTCGCAAACCGACTTAAGTCTTTATTTTCTAATAGGCTTGGCCT 955
NUMT_3          CATTTCTTGTTTGGGGTTCGCAAACCGACTTAAGTCTTTATTTTCTAATAGGCTTGGCCT 955
NA              CATTTCTTGTTTGGGGTTCGCAAACCGACTTAAGTCTTTATTTTCTAATAGGCTTGGCCT 955
Zmp             CATTTCTTGTTTGGGGTTCGCAAACCGACTTAAGTCTTTATTTTCTAATAGGCTTGGCCT 955
CMS-S           CATTTCTTGTTTGGGGTTCGCAAACCGACTTAAGTCTTTATTTTCTAATAGGCTTGGCCT 955
NUMT_2          CATTTCTTGTTTGGGGTTCGCAAACCGACTTAAGTCTTTATTTTCTAATAGGCTTGGCCT 955
CMS-T           CATTTCTTGTTTGGGGTTCGCAAACCGACTTAAGTCTTTATTTTCTAATAGGCTTGGCCT 956
                ************************************************************
```

```
NUMT_1          TCGGGGGAACAAAGCCATTCCATGGAACCTGAGACTTTGATTCCAGCCTACTTCTACTTG 1015
NUMT_3          TCGGGGGAACAAAGCCATTCCATGGAACCTGAGACTTTGATTCCAGCCTACTTCTACTTG 1015
NA              TCGGGGGAACAAAGCCATTCCATGGAACCTGAGACTTTGATTCCAGCCTACTTCTACTTG 1015
Zmp             TCGGGGGAACAAAGCCATTCCATGGAACCTGAGACTTTGATTCCAGCCTACTTCTACTTG 1015
CMS-S           TCGGGGGAACAAAGCCATTCCATGGAACCTGAGACTTTGATTCCAGCCTACTTCTACTTG 1015
NUMT_2          TCGGGGGAACAAAGCCATTCCATGGAACCTGAGACTTTGATTCCAGCCTACTTCTACTTG 1015
CMS-T           TCGGGGGAACAAAGCCATTCCATGGAACCTGAGACTTTGATTCCAGCCTACTTCTACTTG 1016
                ************************************************************

NUMT_1          AAAAGCTTCTTCGTCGACAAATTCTTAGACCCCGTTGTCCAGTTTTGAACGATGGGAAGT 1075
NUMT_3          AAAAGCTTCTTCGTCGACAAATTCTTAGACCCCGTTGTCCAGTTTTGAACGATGGGAAGT 1075
NA              AAAAGCTTCTTCGTCGACAAATTCTTAGACCCCGTTGTCCAGTTTTGAACGATGGGAAGT 1075
Zmp             AAAAGCTTCTTCGTCGACAAATTCTTAGACCCCGTTGTCCAGTTTTGAACGATGGGAAGT 1075
CMS-S           AAAAGCTTCTTCGTCGACAAATTCTTAGACCCCGTTGTCCAGTTTTGAACGATGGGAAGT 1075
NUMT_2          AAAAGCTTCTTCGTCGACAAATTCTTAGACCCCGTTGTCCAGTTTTGAACGATGGGAAGT 1075
CMS-T           AAAAGCTTCTTCGTCGACAAATTCTTAGACCCCGTTGTCCAGTTTTGAACGATGGGAAGT 1076
                ************************************************************

NUMT_1          AGAAATATCTTTCCCACATTAGCGATTTGAGCAACCTCCGTTGAAAGCTTTCTTTGCCCC 1135
NUMT_3          AGAAATATCTTTCCCACATTAGCGATTTGAGCAACCTCCGTTGAAAGCTTTCTTTGCCCC 1135
NA              AGAAATATCTTTCCCACATTAGCGATTTGAGCAACCTCCGTTGAAAGCTTTCTTTGCCCC 1135
Zmp             AGAAATATCTTTCCCACATTAGCGATTTGAGCAACCTCCGTTGAAAGCTTTCTTTGCCCC 1135
CMS-S           AGAAATATCTTTCCCACATTAGCGATTTGAGCAACCTCCGTTGAAAGCTTTCTTTGCCCC 1135
NUMT_2          AGAAATATCTTTCCCACATTAGCGATTTGAGCAACCTCCGTTGAAAGCTTTCTTTGCCCC 1135
CMS-T           AGAAATATCTTTCCCACATTAGCGATTTGAGCAACCTCCGTTGAAAGCTTTCTTTGCCCC 1136
                ************************************************************

NUMT_1          GCCAAAGGAAAGGGCATTTGTCATCCTTGGTCTTCGTTCAGGCAAGTCTAATCACACAAG 1195
NUMT_3          GCCAAAGGAAAGGGCATTTGTCATCCTTGGTCTTCGTTCAGGCAAGTCTAATCACACAAG 1195
NA              GCCAAAGGAAAGGGCATTTGTCATCCTTGGTCTTCGTTCAGGCAAGTCTAATCACACAAG 1195
Zmp             GCCAAAGGAAAGGGCATTTGTCATCCTTGGTCTTCGTTCAGGCAAGTCTAATCACACAAG 1195
CMS-S           GCCAAAGGAAAGGGCATTTGTCATCCTTGGTCTTCGTTCAGGCAAGTCTAATCACACAAG 1195
NUMT_2          GCCAAAGGAAAGGGCATTTGTCATCCTTGGTCTTCGTTCAGGCAAGTCTAATCACACAAG 1195
CMS-T           GCCAAAGGAAAGGGCATTTGTCATCCTTGGTCTTCGTTCAGGCAAGTCTAATCACACAAG 1196
                ************************************************************

NUMT_1          TGAGATAGAAACACTGATTCGGGAGTATCAACTAATTTCATTTAAATGGCTGGGAAAGAA 1255
NUMT_3          TGAGATAGAAACACTGATTCGGGAGTATCAACTAATTTCATTTAAATGGCTGGGAAAGAA 1255
NA              TGAGATAGAAACACTGATTCGGGAGTATCAACTAATTTCATTTAAATGGCTGGGAAAGAA 1255
Zmp             TGAGATAGAAACACTGATTCGGGAGTATCAACTAATTTCATTTAAATGGCTGGGAAAGAA 1255
CMS-S           TGAGATAGAAACACTGATTCGGGAGTATCAACTAATTTCATTTAAATGGCTGGGAAAGAA 1255
NUMT_2          TGAGATAGAAACACTGATTCGGGAGTATCAACTAATTTCATTTAAATGGCTGGGAAAGAA 1255
CMS-T           TGAGATAGAAACACTGATTCGGGAGTATCAACTAATTTCATTTAAATGGCTGGGAAAGAA 1256
                ************************************************************

NUMT_1          ATCAACTAATTGGTTGGTAGCCCGCTTGGTACTAAGAGGTTCCTTGCCCTTCTTCCATTT 1315
NUMT_3          ATCAACTAATTGGTTGGTAGCCCGCTTGGTACTAAGAGGTTCCTTGCCCTTCTTCCATTT 1315
NA              ATCAACTAATTGGTTGGTAGCCCGCTTGGTACTAAGAGGTTCCTTGCCCTTCTTCCATTT 1315
Zmp             ATCAACTAATTGGTTGGTAGCCCGCTTGGTACTAAGAGGTTCCTTGCCCTTCTTCCATTT 1315
CMS-S           ATCAACTAATTGGTTGGTAGCCCGCTTGGTACTAAGAGGTTCCTTGCCCTTCTTCCATTT 1315
NUMT_2          ATCAACTAATTGGTTGGTAGCCCGCTTGGTACTAAGAGGTTCCTTGCCCTTCTTCCATTT 1315
CMS-T           ATCAACTAATTGGTTGGTAGCCCGCTTGGTACTAAGAGGTTCCTTGCCCTTCTTCCATTT 1316
                ************************************************************

NUMT_1          GGTAAAGTGATATTTTTAGGATTTGCTGGCAGGGAAGTATGTTTGTAGGTCAAAGTTGGA 1375
NUMT_3          GGTAAAGTGATATTTTTAGGATTTGCTGGCAGGGAAGTATGTTTGTAGGTCAAAGTTGGA 1375
NA              GGTAAAGTGATATTTTTAGGATTTGCTGGCAGGGAAGTATGTTTGTAGGTCAAAGTTGGA 1375
Zmp             GGTAAAGTGATATTTTTAGGATTTGCTGGCAGGGAAGTATGTTTGTAGGTCAAAGTTGGA 1375
CMS-S           GGTAAAGTGATATTTTTAGGATTTGCTGGCAGGGAAGTATGTTTGTAGGTCAAAGTTGGA 1375
NUMT_2          GGTAAAGTGATATTTTTAGGATTTGCTGGCAGGGAAGTATGTTTGTAGGTCAAAGTTGGA 1375
CMS-T           GGTAAAGTGATATTTTTAGGATTTGCTGGCAGGGAAGTATGTTTGTAGGTCAAAGTTGGA 1376
                ************************************************************

NUMT_1          ATTAGAATCGGGGAACTCGGTAAAGTAGCTTTCATAGCTTTCACATATTTGTGAGCCCTA 1435
NUMT_3          ATTAGAATCGGGGAACTCGGTAAAGTAGCTTTCATAGCTTTCACATATTTGTGAGCCCTA 1435
NA              ATTAGAATCGGGGAACTCGGTAAAGTAGCTTTCATAGCTTTCACATATTTGTGAGCCCTA 1435
Zmp             ATTAGAATCGGGGAACTCGGTAAAGTAGCTTTCATAGCTTTCACATATTTGTGAGCCCTA 1435
CMS-S           ATTAGAATCGGGGAACTCGGTAAAGTAGCTTTCATAGCTTTCACATATTTGTGAGCCCTA 1435
NUMT_2          ATTAGAATCGGGGAACTCGGTAAAGTAGCTTTCATAGCTTTCACATATTTGTGAGCCCTA 1435
CMS-T           ATTAGAATCGGGGAACTCGGTAAAGTAGCTTTCATAGCTTTCACATATTTGTGAGCCCTA 1436
                ************************************************************
```

```
NUMT_1          TGGGTAGATAGAAGAAGGTACCCGACATCATCCACCTTACGTTGAGACAGAAGAGAAAGA 1495
NUMT_3          TGGGTAGATAGAAGAAGGTACCCGACATCATCCACCTTACGTTGAGACAGAAGAGAAAGA 1495
NA              TGGGTAGATAGAAGAAGGTACCCGACATCATCCACCTTACGTTGAGACAGAAGAGAAAGA 1495
Zmp             TGGGTAGATAGAAGAAGGTACCCGACATCATCCACCTTACGTTGAGACAGAAGAGAAAGA 1495
CMS-S           TGGGTAGATAGAAGAAGGTACCCGACATCATCCACCTTACGTTGAGACAGAAGAGAAAGA 1495
NUMT_2          TGGGTAGATAGAAGAAGGTACCCGACATCATCCACCTTACGTTGAGACAGAAGAGAAAGA 1495
CMS-T           TGGGTAGATAGAAGAAGGTACCCGACATCATCCACCTTACGTTGAGACAGAAGAGAAAGA 1496
                ************************************************************

NUMT_1          AGTTTATTCCCTCGCTAGCAAGCTTCTTTTCTTCTACTACCTGATGATTGAATTGGACTT 1555
NUMT_3          AGTTTATTCCCTCGCTAGCAAGCTTCTTTTCTTCTACTACCTGATGATTGAATTGGACTT 1555
NA              AGTTTATTCCCTCGCTAGCAAGCTTCTTTTCTTCTACTACCTGATGATTGAATTGGACTT 1555
Zmp             AGTTTATTCCCTCGCTAGCAAGCTTCTTTTCTTCTACTACCTGATGATTGAATTGGACTT 1555
CMS-S           AGTTTATTCCCTCGCTAGCAAGCTTCTTTTCTTCTACTACCTGATGATTGAATTGGACTT 1555
NUMT_2          AGTTTATTCCCTCGCTAGCAAGCTTCTTTTCTTCTACTACCTGATGATTGAATTGGACTT 1555
CMS-T           AGTTTATTCCCTCGCTAGCAAGCTTCTTTTCTTCTACTACCTGATGATTGAATTGGACTT 1556
                ************************************************************

NUMT_1          CCTTCCCCACAGAACCCAAGATGGTTGACTACTAGGCTCACAACTCTACGCAAAGGTAGG 1615
NUMT_3          CCTTCCCCACAGAACCCAAGATGGTTGACTACTAGGCTCACAACTCTACGCAAAGGTAGG 1615
NA              CCTTCCCCACAGAACCCAAGATGGTTGACTACTAGGCTCACAACTCTACGCAAAGGTAGG 1615
Zmp             CCTTCCCCACAGAACCCAAGATGGTTGACTACTAGGCTCACAACTCTACGCAAAGGTAGG 1615
CMS-S           CCTTCCCCACAGAACCCAAGATGGTTGACTACTAGGCTCACAACTCTACGCAAAGGTAGG 1615
NUMT_2          CCTTCCCCACAGAACCCAAGATGGTTGACTACTAGGCTCACAACTCTACGCAAAGGTAGG 1615
CMS-T           CCTTCCCCACAGAACCCAAGATGGTTGACTACTAGGCTCACAACTCTACGCAAAGGTAGG 1616
                ************************************************************

NUMT_1          CTCGATGAAGAAAGCGCAGGGTTACTTTCTTGCCTGAGGAAGTAGTTAGAAGTACTTTCT 1675
NUMT_3          CTCGATGAAGAAAGCGCAGGGTTACTTTCTTGCCTGAGGAAGTAGTTAGAAGTACTTTCT 1675
NA              CTCGATGAAGAAAGCGCAGGGTTACTTTCTTGCCTGAGGAAGTAGTTAGAAGTACTTTCT 1675
Zmp             CTCGATGAAGAAAGCGCAGGGTTACTTTCTTGCCTGAGGAAGTAGTTAGAAGTACTTTCT 1675
CMS-S           CTCGATGAAGAAAGCGCAGGGTTACTTTCTTGCCTGAGGAAGTAGTTAGAAGTACTTTCT 1675
NUMT_2          CTCGATGAAGAAAGCGCAGGGTTACTTTCTTGCCTGAGGAAGTAGTTAGAAGTACTTTCT 1675
CMS-T           CTCGATGAAGAAAGCGCAGGGTTACTTTCTTGCCTGAGGAAGTAGTTAGAAGTACTTTCT 1676
                ************************************************************

NUMT_1          TATTAATCTGCTTGATGTACATAAATCGATGGTTAAGGCGCGCAGCGGTAAGGTTCCCAC 1735
NUMT_3          TATTAATCTGCTTGATGTACATAAATCGATGGTTAAGGCGCGCAGCGGTAAGGTTCCCAC 1735
NA              TATTAATCTGCTTGATGTACATAAATCGATGGTTAAGGCGCGCAGCGGTAAGGTTCCCAC 1735
Zmp             TATTAATCTGCTTGATGTACATAAATCGATGGTTAAGGCGCGCAGCGGTAAGGTTCCCAC 1735
CMS-S           TATTAATCTGCTTGATGTACATAAATCGATGGTTAAGGCGCGCAGCGGTAAGGTTCCCAC 1735
NUMT_2          TATTAATCTGCTTGATGTACATAAATCGATGGTTAAGGCGCGCAGCGGTAAGGTTCCCAC 1735
CMS-T           TATTAATCTGCTTGATGTACATAAATCGATGGTTAAGGCGCGCAGCGGTAAGGTTCCCAC 1736
                ************************************************************

NUMT_1          CGAATCAAGAATTCGGCAATTGAGGAGCTCGATTAGTCATACTCACCACCACCAGCTCTA 1795
NUMT_3          CGAATCAAGAATTCGGCAATTGAGGAGCTCGATTAGTCATACTCACCACCACCAGCTCTA 1795
NA              CGAATCAAGAATTCGGCAATTGAGGAGCTCGATTAGTCATACTCACCACCACCAGCTCTA 1795
Zmp             CGAATCAAGAATTCGGCAATTGAGGAGCTCGATTAGTCATACTCACCACCACCAGCTCTA 1795
CMS-S           CGAATCAAGAATTCGGCAATTGAGGAGCTCGATTAGTCATACTCACCACCACCAGCTCTA 1795
NUMT_2          CGAATCAAGAATTCGGCAATTGAGGAGCTCGATTAGTCATACTCACCACCACCAGCTCTA 1795
CMS-T           CGAATCAAGAATTCGGCAATTGAGGAGCTCGATTAGTCATACTCACCACCACCAGCTCTA 1796
                ************************************************************

NUMT_1          GGCCCATCTTTTATATCTATACCGGAAAAGCGCTTCGCTTGATAACGGCATTCATAAATG 1855
NUMT_3          GGCCCATCTTTTATATCTATACCGGAAAAGCGCTTCGCTTGATAACGGCATTCATAAATG 1855
NA              GGCCCATCTTTTATATCTATACCGGAAAAGCGCTTCGCTTGATAACGGCATTCATAAATG 1855
Zmp             GGCCCATCTTTTATATCTATACCGGAAAAGCGCTTCGCTTGATAACGGCATTCATAAATG 1855
CMS-S           GGCCCATCTTTTATATCTATACCGGAAAAGCGCTTCGCTTGATAACGGCATTCATAAATG 1855
NUMT_2          GGCCCATCTTTTATATCTATACCGGAAAAGCGCTTCGCTTGATAACGGCATTCATAAATG 1855
CMS-T           GGCCCATCTTTTATATCTATACCGGAAAAGCGCTTCGCTTGATAACGGCATTCATAAATG 1856
                ************************************************************

NUMT_1          AAAGGGAACTTCTACTCAGGCAGGCCCATTGGTTTAGTCAAGCCATCCCGTTGAAAAAA  1914
NUMT_3          AAAGGGAACTTCTACTCAGGCAGGCCCATTGGTTTAGTCAAGCCATCCCGTTGAAAAAA  1914
NA              AAAGGGAACTTCTACTCAGGCAGGCCCATTGGTTTAGTCAAGCCATCCCGTTGAAAAAAA 1915
Zmp             AAAGGGAACTTCTACTCAGGCAGGCCCATTGGTTTAGTCAAGCCATCCCGTTGAAAAAAA 1915
CMS-S           AAAGGGAACTTCTACTCAGGCAGGCCCATTGGTTTAGTCAAGCCATCCCGTTGAAAAAAA 1915
NUMT_2          AAAGGGAACTTCTACTCAGGCAGGCCCATTGGTTTAGTCAAGCCATCCCGTTGAAAAAAA 1915
CMS-T           AAAGGGAACTTCTACTCAGGCAGGCCCATTGGTTTAGTCAAGCCATCCCGTTGAAAAAAA 1916
                ***********************************************************
```

```
NUMT_1          TCCTCTGTTTTCTATCAGAGAAGTCTAACTATTCCACTTGGCTTAAGAGAGATAGATACC 1974
NUMT_3          TCCTCTGTTTTCTATCAGAGAAGTCTAACTATTCCACTTGGCTTAAGAGAGATAGATACC 1974
NA              TCCTCTGTTTTCTATCAGAGAAGTCTAACTATTCCACTTGGCTTAAGAGAGATAGATACC 1975
Zmp             TCCTCTGTTTTCTATCAGAGAAGTCTAACTATTCCACTTGGCTTAAGAGAGATAGATACC 1975
CMS-S           TCCTCTGTTTTCTATCAGAGAAGTCTAACTATTCCACTTGGCTTAAGAGAGATAGATACC 1975
NUMT_2          TCCTCTGTTTTCTATCAGAGAAGTCTAACTATTCCACTTGGCTTAAGAGAGATAGATACC 1975
CMS-T           TCCTCTGTTTTCTATCAGAGAAGTCTAACTATTCCACTTGGCTTAAGAGAGATAGATACC 1976
                ************************************************************

NUMT_1          GATACGCCCTCTTTTGCGCCAACAAGTCCCCGTGACGATAGTAGTCTTTATTCAGAAGGG 2034
NUMT_3          GATACGCCCTCTTTTGCGCCAACAAGTCCCCGTGACGATAGTAGTCTTTATTCAGAAGGG 2034
NA              GATACGCCCTCTTTTGCGCCAACAAGTCCCCGTGACGATAGTAGTCTTTATTCAGAAGGG 2035
Zmp             GATACGCCCTCTTTTGCGCCAACAAGTCCCCGTGACGATAGTAGTCTTTATTCAGAAGGG 2035
CMS-S           GATACGCCCTCTTTTGCGCCAACAAGTCCCCGTGACGATAGTAGTCTTTATTCAGAAGGG 2035
NUMT_2          GATACGCCCTCTTTTGCGCCAACAAGTCCCCGTGACGATAGTAGTCTTTATTCAGAAGGG 2035
CMS-T           GATACGCCCTCTTTTGCGCCAACAAGTCCCCGTGACGATAGTAGTCTTTATTCAGAAGGG 2036
                ************************************************************

NUMT_1          CAGGGTGAGATAGGTAGCCACACCTATGTATGATCAAGCTAAAGTATACATCATGGCTGT 2094
NUMT_3          CAGGGTGAGATAGGTAGCCACACCTATGTATGATCAAGCTAAAGTATACATCATGGCTGT 2094
NA              CAGGGTGAGATAGGTAGCCACACCTATGTATGATCAAGCTAAAGTATACATCATGGCTGT 2095
Zmp             CAGGGTGAGATAGGTAGCCACACCTATGTATGATCAAGCTAAAGTATACATCATGGCTGT 2095
CMS-S           CAGGGTGAGATAGGTAGCCACACCTATGTATGATCAAGCTAAAGTATACATCATGGCTGT 2095
NUMT_2          CAGGGTGAGATAGGTAGCCACACCTATGTATGATCAAGCTAAAGTATACATCATGGCTGT 2095
CMS-T           CAGGGTGAGATAGGTAGCCACACCTATGTATGATCAAGCTAAAGTATACATCATGGCTGT 2096
                ************************************************************

NUMT_1          ATCTGCCAGAGCGCCCTTCTTATGCCAATTAGCAGGTTTGTTACAAATCATGGGAACAAA 2154
NUMT_3          ATCTGCCAGAGCGCCCTTCTTATGCCAATTAGCAGGTTTGTTACAAATCATGGGAACAAA 2154
NA              ATCTGCCAGAGCGCCCTTCTTATGCCAATTAGCAGGTTTGTTACAAATCATGGGAACAAA 2155
Zmp             ATCTGCCAGAGCGCCCTTCTTATGCCAATTAGCAGGTTTGTTACAAATCATGGGAACAAA 2155
CMS-S           ATCTGCCAGAGCGCCCTTCTTATGCCAATTAGCAGGTTTGTTACAAATCATGGGAACAAA 2155
NUMT_2          ATCTGCCAGAGCGCCCTTCTTATGCCAATTAGCAGGTTTGTTACAAATCATGGGAACAAA 2155
CMS-T           ATCTGCCAGAGCGCCCTTCTTATGCCAATTAGCAGGTTTGTTACAAATCATGGGAACAAA 2156
                ************************************************************

NUMT_1          TATAAAATACAAGACTCTGATCTACTCAAAGGTCAGATGGGTTGGTTGAATTTCTTCATA 2214
NUMT_3          TATAAAATACAAGACTCTGATCTACTCAAAGGTCAGATGGGTTGGTTGAATTTCTTCATA 2214
NA              TATAAAATACAAGACTCTGATCTACTCAAAGGTCAGATGGGTTGGTTGAATTTCTTCATA 2215
Zmp             TATAAAATACAAGACTCTGATCTACTCAAAGGTCAGATGGGTTGGTTGAATTTCTTCATA 2215
CMS-S           TATAAAATACAAGACTCTGATCTACTCAAAGGTCAGATGGGTTGGTTGAATTTCTTCATA 2215
NUMT_2          TATAAAATACAAGACTCTGATCTACTCAAAGGTCAGATGGGTTGGTTGAATTTCTTCATA 2215
CMS-T           TATAAAATACAAGACTCTGATCTACTCAAAGGTCAGATGGGTTGGTTGAATTTCTTCATA 2216
                ************************************************************

NUMT_1          AAGAGTAGGCTTCTATGCCGCTATCTATGCCACAAGGCTATCCGAAGCGAGCCATAAGAG 2274
NUMT_3          AAGAGTAGGCTTCTATGCCGCTATCTATGCCACAAGGCTATCCGAAGCGAGCCATAAGAG 2274
NA              AAGAGTAGGCTTCTATGCCGCTATCTATGCCACAAGGCTATCCGAAGCGAGCCATAAGAG 2275
Zmp             AAGAGTAGGCTTCTATGCCGCTATCTATGCCACAAGGCTATCCGAAGCGAGCCATAAGAG 2275
CMS-S           AAGAGTAGGCTTCTATGCCGCTATCTATGCCACAAGGCTATCCGAAGCGAGCCATAAGAG 2275
NUMT_2          AAGAGTAGGCTTCTATGCCGCTATCTATGCCACAAGGCTATCCGAAGCGAGCCATAAGAG 2275
CMS-T           AAGAGTAGGCTTCTATGCCGCTATCTATGCCACAAGGCTATCCGAAGCGAGCCATAAGAG 2276
                ************************************************************

NUMT_1          AGCCTTGTCCTAGTATTAGGAGCGATGGAGCTTTTCCAGTGAAAGGAATAC TAGCGAGT 2334
NUMT_3          AGCCTTGTCCTAGTATTAGGAGCGATGGAGCTTTTCCAGTGAAAGGAATAC TAGCGAGT 2334
NA              AGCCTTGTCCTAGTATTAGGAGCGATGGAGCTTTTCCAGTGAAAGGAATACGTAGCGAGT 2335
Zmp             AGCCTTGTCCTAGTATTAGGAGCGATGGAGCTTTTCCAGTGAAAGGAATACGTAGCGAGT 2335
CMS-S           AGCCTTGTCCTAGTATTAGGAGCGATGGAGCTTTTCCAGTGAAAGGAATACGTAGCGAGT 2335
NUMT_2          AGCCTTGTCCTAGTATTAGGAGCGATGGAGCTTTTCCAGTGAAAGGAATACGTAGCGAGT 2335
CMS-T           AGCCTTGTCCTAGTATTAGGAGCGATGGAGCTTTTCCAGTGAAAGGAATACGTAGCGAGT 2336
                *************************************************** ********

NUMT_1          CACGGGAATAATAGAAAAGCACTCTTCGGGGGCTCACTCTCGCCTCTATTACATAACCTT 2394
NUMT_3          CACGGGAATAATAGAAAAGCACTCTTCGGGGGCTCACTCTCGCCTCTATTACATAACCTT 2394
NA              CACGGGAATAATAGAAAAGCACTCTTCGGGGGCTCACTCTCGCCTCTATTACATAACCTT 2395
Zmp             CACGGGAATAATAGAAAAGCACTCTTCGGGGGCTCACTCTCGCCTCTATTACATAACCTT 2395
CMS-S           CACGGGAATAATAGAAAAGCACTCTTCGGGGGCTCACTCTCGCCTCTATTACATAACCTT 2395
NUMT_2          CACGGGAATAATAGAAAAGCACTCTTCGGGGGCTCACTCTCGCCTCTATTACATAACCTT 2395
CMS-T           CACGGGAATAATAGAAAAGCACTCTTCGGGGGCTCACTCTCGCCTCTATTACATAACCTT 2396
                ************************************************************
```

```
NUMT_1          TCCCCGGTATACTCCCCCTTCGAGATGGTCACTCAACCTATTGAAGAGCCTGGCATGGAA 2454
NUMT_3          TCCCCGGTATACTCCCCCTTCGAGATGGTCACTCAACCTATTGAAGAGCCTGGCATGGAA 2454
NA              TCCCCGGTATACTCCCCCTTCGAGATGGTCACTCAACCTATTGAAGAGCCTGGCATGGAA 2455
Zmp             TCCCCGGTATACTCCCCCTTCGAGATGGTCACTCAACCTATTGAAGAGCCTGGCATGGAA 2455
CMS-S           TCCCCGGTATACTCCCCCTTCGAGATGGTCACTCAACCTATTGAAGAGCCTGGCATGGAA 2455
NUMT_2          TCCCCGGTATACTCCCCCTTCGAGATGGTCACTCAACCTATTGAAGAGCCTGGCATGGAA 2455
CMS-T           TCCCCGGTATACTCCCCCTTCGAGATGGTCACTCAACCTATTGAAGAGCCTGGCATGGAA 2456
                ************************************************************

NUMT_1          GACCATCTTTAAGAGAGAAAGTTCCTTGCCAAGAGCAATCCAAGGCCCTCTCAACCCAAG 2514
NUMT_3          GACCATCTTTAAGAGAGAAAGTTCCTTGCCAAGAGCAATCCAAGGCCCTCTCAACCCAAG 2514
NA              GACCATCTTTAAGAGAGAAAGTTCCTTGCCAAGAGCAATCCAAGGCCCTCTCAACCCAAG 2515
Zmp             GACCATCTTTAAGAGAGAAAGTTCCTTGCCAAGAGCAATCCAAGGCCCTCTCAACCCAAG 2515
CMS-S           GACCATCTTTAAGAGAGAAAGTTCCTTGCCAAGAGCAATCCAAGGCCCTCTCAACCCAAG 2515
NUMT_2          GACCATCTTTAAGAGAGAAAGTTCCTTGCCAAGAGCAATCCAAGGCCCTCTCAACCCAAG 2515
CMS-T           GACCATCTTTAAGAGAGAAAGTTCCTTGCCAAGAGCAATCCAAGGCCCTCTCAACCCAAG 2516
                ************************************************************

NUMT_1          ACAAGAGGTCTTGAGTCCATATGGTTACCACCTAAAGCGGATACCGTAAAATCGAGGTAG 2574
NUMT_3          ACAAGAGGTCTTGAGTCCATATGGTTACCACCTAAAGCGGATACCGTAAAATCGAGGTAG 2574
NA              ACAAGAGGTCTTGAGTCCATATGGTTACCACCTAAAGCGGATACCGTAAAATCGAGGTAG 2575
Zmp             ACAAGAGGTCTTGAGTCCATATGGTTACCACCTAAAGCGGATACCGTAAAATCGAGGTAG 2575
CMS-S           ACAAGAGGTCTTGAGTCCATATGGTTACCACCTAAAGCGGATACCGTAAAATCGAGGTAG 2575
NUMT_2          ACAAGAGGTCTTGAGTCCATATGGTTACCACCTAAAGCGGATACCGTAAAATCGAGGTAG 2575
CMS-T           ACAAGAGGTCTTGAGTCCATATGGTTACCACCTAAAGCGGATACCGTAAAATCGAGGTAG 2576
                ************************************************************

NUMT_1          AAATCTCTAGACCTTCCCCCCAGGCGTATAAACGGTTATTGAAAAAACGGAACGATGTTG 2634
NUMT_3          AAATCTCTAGACCTTCCCCCCAGGCGTATAAACGGTTATTGAAAAAACGGAACGATGTTG 2634
NA              AAATCTCTAGACCTTCCCCCCAGGCGTATAAACGGTTATTGAAAAAACGGAACGATGTTG 2635
Zmp             AAATCTCTAGACCTTCCCCCCAGGCGTATAAACGGTTATTGAAAAAACGGAACGATGTTG 2635
CMS-S           AAATCTCTAGACCTTCCCCCCAGGCGTATAAACGGTTATTGAAAAAACGGAACGATGTTG 2635
NUMT_2          AAATCTCTAGACCTTCCCCCCAGGCGTATAAACGGTTATTGAAAAAACGGAACGATGTTG 2635
CMS-T           AAATCTCTAGACCTTCCCCCCAGGCGTATAAACGGTTATTGAAAAAACGGAACGATGTTG 2636
                ************************************************************

NUMT_1          CTTTGCATGGGAAGCTGCCACCTGTCTGTAATAGGAACCGAGTCGCTAAAGGAGTTTCAT 2694
NUMT_3          CTTTGCATGGGAAGCTGCCACCTGTCTGTAATAGGAACCGAGTCGCTAAAGGAGTTTCAT 2694
NA              CTTTGCATGGGAAGCTGCCACCTGTCTGTAATAGGAACCGAGTCGCTAAAGGAGTTTCAT 2695
Zmp             CTTTGCATGGGAAGCTGCCACCTGTCTGTAATAGGAACCGAGTCGCTAAAGGAGTTTCAT 2695
CMS-S           CTTTGCATGGGAAGCTGCCACCTGTCTGTAATAGGAACCGAGTCGCTAAAGGAGTTTCAT 2695
NUMT_2          CTTTGCATGGGAAGCTGCCACCTGTCTGTAATAGGAACCGAGTCGCTAAAGGAGTTTCAT 2695
CMS-T           CTTTGCATGGGAAGCTGCCACCTGTCTGTAATAGGAACCGAGTCGCTAAAGGAGTTTCAT 2696
                ************************************************************

NUMT_1          CAGCATCGAGTTATTGAGCCCACGGAACGGGGAGTGTTAACGAGTCACTAACCCGTAGTG 2754
NUMT_3          CAGCATCGAGTTATTGAGCCCACGGAACGGGGAGTGTTAACGAGTCACTAACCCGTAGTG 2754
NA              CAGCATCGAGTTATTGAGCCCACGGAACGGGGAGTGTTAACGAGTCACTAACCCGTAGTG 2755
Zmp             CAGCATCGAGTTATTGAGCCCACGGAACGGGGAGTGTTAACGAGTCACTAACCCGTAGTG 2755
CMS-S           CAGCATCGAGTTATTGAGCCCACGGAACGGGGAGTGTTAACGAGTCACTAACCCGTAGTG 2755
NUMT_2          CAGCATCGAGTTATTGAGCCCACGGAACGGGGAGTGTTAACGAGTCACTAACCCGTAGTG 2755
CMS-T           CAGCATCGAGTTATTGAGCCCACGGAACGGGGAGTGTTAACGAGTCACTAACCCGTAGTG 2756
                ************************************************************

NUMT_1          CTTCCTAAACCTCGCGATTCGACCGTTGGAGCTGAGTCAATCTTGCCAAATTGGCAAATG 2814
NUMT_3          CTTCCTAAACCTCGCGATTCGACCGTTGGAGCTGAGTCAATCTTGCCAAATTGGCAAATG 2814
NA              CTTCCTAAACCTCGCGATTCGACCGTTGGAGCTGAGTCAATCTTGCCAAATTGGCAAATG 2815
Zmp             CTTCCTAAACCTCGCGATTCGACCGTTGGAGCTGAGTCAATCTTGCCAAATTGGCAAATG 2815
CMS-S           CTTCCTAAACCTCGCGATTCGACCGTTGGAGCTGAGTCAATCTTGCCAAATTGGCAAATG 2815
NUMT_2          CTTCCTAAACCTCGCGATTCGACCGTTGGAGCTGAGTCAATCTTGCCAAATTGGCAAATG 2815
CMS-T           CTTCCTAAACCTCGCGATTCGACCGTTGGAGCTGAGTCAATCTTGCCAAATTGGCAAATG 2816
                ************************************************************

NUMT_1          TCTGGGTCTGTCCTATAGAATAGCTCATGGAACTTGCTTTCCAGATCGTACCAACTGTTA 2874
NUMT_3          TCTGGGTCTGTCCTATAGAATAGCTCATGGAACTTGCTTTCCAGATCGTACCAACTGTTA 2874
NA              TCTGGGTCTGTCCTATAGAATAGCTCATGGAACTTGCTTTCCAGATCGTACCAACTGTTA 2875
Zmp             TCTGGGTCTGTCCTATAGAATAGCTCATGGAACTTGCTTTCCAGATCGTACCAACTGTTA 2875
CMS-S           TCTGGGTCTGTCCTATAGAATAGCTCATGGAACTTGCTTTCCAGATCGTACCAACTGTTA 2875
NUMT_2          TCTGGGTCTGTCCTATAGAATAGCTCATGGAACTTGCTTTCCAGATCGTACCAACTGTTA 2875
CMS-T           TCTGGGTCTGTCCTATAGAATAGCTCATGGAACTTGCTTTCCAGATCGTACCAACTGTTA 2876
                ************************************************************
```

```
NUMT_1          ATAGAGTTTGGAGAGATGCTCATATACCAGGGGAAGGCCGATTTAGTAAGAGAGTTGGCA 2934
NUMT_3          ATAGAGTTTGGAGAGATGCTCATATACCAGGGGAAGGCCGATTTAGTAAGAGAGTTGGCA 2934
NA              ATAGAGTTTGGAGAGATGCTCATATACCAGGGGAAGGCCGATTTAGTAAGAGAGTTGGCA 2935
Zmp             ATAGAGTTTGGAGAGATGCTCATATACCAGGGGAAGGCCGATTTAGTAAGAGAGTTGGCA 2935
CMS-S           ATAGAGTTTGGAGAGATGCTCATATACCAGGGGAAGGCCGATTTAGTAAGAGAGTTGGCA 2935
NUMT_2          ATAGAGTTTGGAGAGATGCTCATATACCAGGGGAAGGCCGATTTAGTAAGAGAGTTGGCA 2935
CMS-T           ATAGAGTTTGGAGAGATGCTCATATACCAGGGGAAGGCCGATTTAGTAAGAGAGTTGGCA 2936
                ************************************************************

NUMT_1          AAGAGCCAAAGCTTGAGGCAGTCCTTGGACCTAGCTTCCCCGCACTGGACCGAGAACCGA 2994
NUMT_3          AAGAGCCAAAGCTTGAGGCAGTCCTTGGACCTAGCTTCCCCGCACTGGACCGAGAACCGA 2994
NA              AAGAGCCAAAGCTTGAGGCAGTCCTTGGACCTAGCTTCCCCGCACTGGACCGAGAACCGA 2995
Zmp             AAGAGCCAAAGCTTGAGGCAGTCCTTGGACCTAGCTTCCCCGCACTGGACCGAGAACCGA 2995
CMS-S           AAGAGCCAAAGCTTGAGGCAGTCCTTGGACCTAGCTTCCCCGCACTGGACCGAGAACCGA 2995
NUMT_2          AAGAGCCAAAGCTTGAGGCAGTCCTTGGACCTAGCTTCCCCGCACTGGACCGAGAACCGA 2995
CMS-T           AAGAGCCAAAGCTTGAGGCAGTCCTTGGACCTAGCTTCCCCGCACTGGACCGAGAACCGA 2996
                ************************************************************

NUMT_1          GCTATATGCTCAATAGTCGATCGACTTATTCTCCTCACCGGAAAAGAGAACAAACTCTGG 3054
NUMT_3          GCTATATGCTCAATAGTCGATCGACTTATTCTCCTCACCGGAAAAGAGAACAAACTCTGG 3054
NA              GCTATATGCTCAATAGTCGATCGACTTATTCTCCTCACCGGAAAAGAGAACAAACTCTGG 3055
Zmp             GCTATATGCTCAATAGTCGATCGACTTATTCTCCTCACCGGAAAAGAGAACAAACTCTGG 3055
CMS-S           GCTATATGCTCAATAGTCGATCGACTTATTCTCCTCACCGGAAAAGAGAACAAACTCTGG 3055
NUMT_2          GCTATATGCTCAATAGTCGATCGACTTATTCTCCTCACCGGAAAAGAGAACAAACTCTGG 3055
CMS-T           GCTATATGCTCAATAGTCGATCGACTTATTCTCCTCACCGGAAAAGAGAACAAACTCTGG 3056
                ************************************************************

NUMT_1          CACCTTGGAAACTGAGGAACTCGGTCGATCCAATCAGGGTATGCTTTCCTAGCCGTCGGC 3114
NUMT_3          CACCTTGGAAACTGAGGAACTCGGTCGATCCAATCAGGGTATGCTTTCCTAGCCGTCGGC 3114
NA              CACCTTGGAAACTGAGGAACTCGGTCGATCCAATCAGGGTATGCTTTCCTAGCCGTCGGC 3115
Zmp             CACCTTGGAAACTGAGGAACTCGGTCGATCCAATCAGGGTATGCTTTCCTAGCCGTCGGC 3115
CMS-S           CACCTTGGAAACTGAGGAACTCGGTCGATCCAATCAGGGTATGCTTTCCTAGCCGTCGGC 3115
NUMT_2          CACCTTGGAAACTGAGGAACTCGGTCGATCCAATCAGGGTATGCTTTCCTAGCCGTCGGC 3115
CMS-T           CACCTTGGAAACTGAGGAACTCGGTCGATCCAATCAGGGTATGCTTTCCTAGCCGTCGGC 3116
                ************************************************************

NUMT_1          CTCAGATGGACGAGACAAAGAACGCAACTCAGGGGGCGAAGGTGGAGGTCCGGGCAAGGC 3174
NUMT_3          CTCAGATGGACGAGACAAAGAACGCAACTCAGGGGGCGAAGGTGGAGGTCCGGGCAAGGC 3174
NA              CTCAGATGGACGAGACAAAGAACGCAACTCAGGGGGCGAAGGTGGAGGTCCGGGCAAGGC 3175
Zmp             CTCAGATGGACGAGACAAAGAACGCAACTCAGGGGGCGAAGGTGGAGGTCCGGGCAAGGC 3175
CMS-S           CTCAGATGGACGAGACAAAGAACGCAACTCAGGGGGCGAAGGTGGAGGTCCGGGCAAGGC 3175
NUMT_2          CTCAGATGGACGAGACAAAGAACGCAACTCAGGGGGCGAAGGTGGAGGTCCGGGCAAGGC 3175
CMS-T           CTCAGATGGACGAGACAAAGAACGCAACTCAGGGGGCGAAGGTGGAGGTCCGGGCAAGGC 3176
                ************************************************************

NUMT_1          TAGAGCCAAGCAGAGGGATGGAGTAGGGGACGGTTGGTGAAAGTAGGAACGAGCGGAATA 3234
NUMT_3          TAGAGCCAAGCAGAGGGATGGAGTAGGGGACGGTTGGTGAAAGTAGGAACGAGCGGAATA 3234
NA              TAGAGCCAAGCAGAGGGATGGAGTAGGGGACGGTTGGTGAAAGTAGGAACGAGCGGAATA 3235
Zmp             TAGAGCCAAGCAGAGGGATGGAGTAGGGGACGGTTGGTGAAAGTAGGAACGAGCGGAATA 3235
CMS-S           TAGAGCCAAGCAGAGGGATGGAGTAGGGGACGGTTGGTGAAAGTAGGAACGAGCGGAATA 3235
NUMT_2          TAGAGCCAAGCAGAGGGATGGAGTAGGGGACGGTTGGTGAAAGTAGGAACGAGCGGAATA 3235
CMS-T           TAGAGCCAAGCAGAGGGATGGAGTAGGGGACGGTTGGTGAAAGTAGGAACGAGCGGAATA 3236
                ************************************************************

NUMT_1          AACGAACGGAATACTCACTCTGTTCTACTCCCCCAGTAGAGCTATATGTAACGAGTCAGA 3294
NUMT_3          AACGAACGGAATACTCACTCTGTTCTACTCCCCCAGTAGAGCTATATGTAACGAGTCAGA 3294
NA              AACGAACGGAATACTCACTCTGTTCTACTCCCCCAGTAGAGCTATATGTAACGAGTCAGA 3295
Zmp             AACGAACGGAATACTCACTCTGTTCTACTCCCCCAGTAGAGCTATATGTAACGAGTCAGA 3295
CMS-S           AACGAACGGAATACTCACTCTGTTCTACTCCCCCAGTAGAGCTATATGTAACGAGTCAGA 3295
NUMT_2          AACGAACGGAATACTCACTCTGTTCTACTCCCCCAGTAGAGCTATATGTAACGAGTCAGA 3295
CMS-T           AACGAACGGAATACTCACTCTGTTCTACTCCCCCAGTAGAGCTATATGTAACGAGTCAGA 3296
                ************************************************************

NUMT_1          CAGACTTTTTACTTGTAGCAAGCGATAAAACGGCTGCACAGTTCTACTGTGCCGAGA 3351
NUMT_3          CAGACTTTTTACTTGTAGCAAGCGATAAAACGGCTGCACAGTTCTACTGTGCCGAGA 3351
NA              CAGACTTTTTACTTGTAGCAAGCGATAAAACGGCTGCACAGTTCTACTGTGCCGAGA 3352
Zmp             CAGACTTTTTACTTGTAGCAAGCGATAAAACGGCTGCACAGTTCTACTGTGCCGAGA 3352
CMS-S           CAGACTTTTTACTTGTAGCAAGCGATAAAACGGCTGCACAGTTCTACTGTGCCGAGA 3352
NUMT_2          CAGACTTTTTACTTGTAGCAAGCGATAAAACGGCTGCACAGTTCTACTGTGCCGAGA 3352
CMS-T           CAGACTTTTTACTTGTAGCAAGCGATAAAACGGCTGCACAGTTCTACTGTGCCGAGA 3353
                *********************************************************
```

Figure S6 Multiple sequence alignment of the three B73 3.3-kb NUMT regions with the corresponding NA, Zmp, CMS-S, and CMS-T mitochondrial genome regions. The 3.3-kb sequence is present within the NA (NCBI Accession DQ490952.1), Zmp (NCBI Accession DQ645539.1), and CMS-S (NCBI Accession DQ490951.2), and CMS-T (NCBI Accession DQ490953.1) mitochondrial genomes. The second copy of the 3.3-kb region within the B73 NUMT has fewer nucleotide differences compared to the NA, Zmp, and CMS-S mitochondrial genomes than the first and third 3.3-kb regions in the NUMT. The sequences were aligned with ClustalW2 (McWilliam *et al.* 2013). The reverse complement of the third 3.3-kb region in the NUMT and the NA genome were used in this alignment.

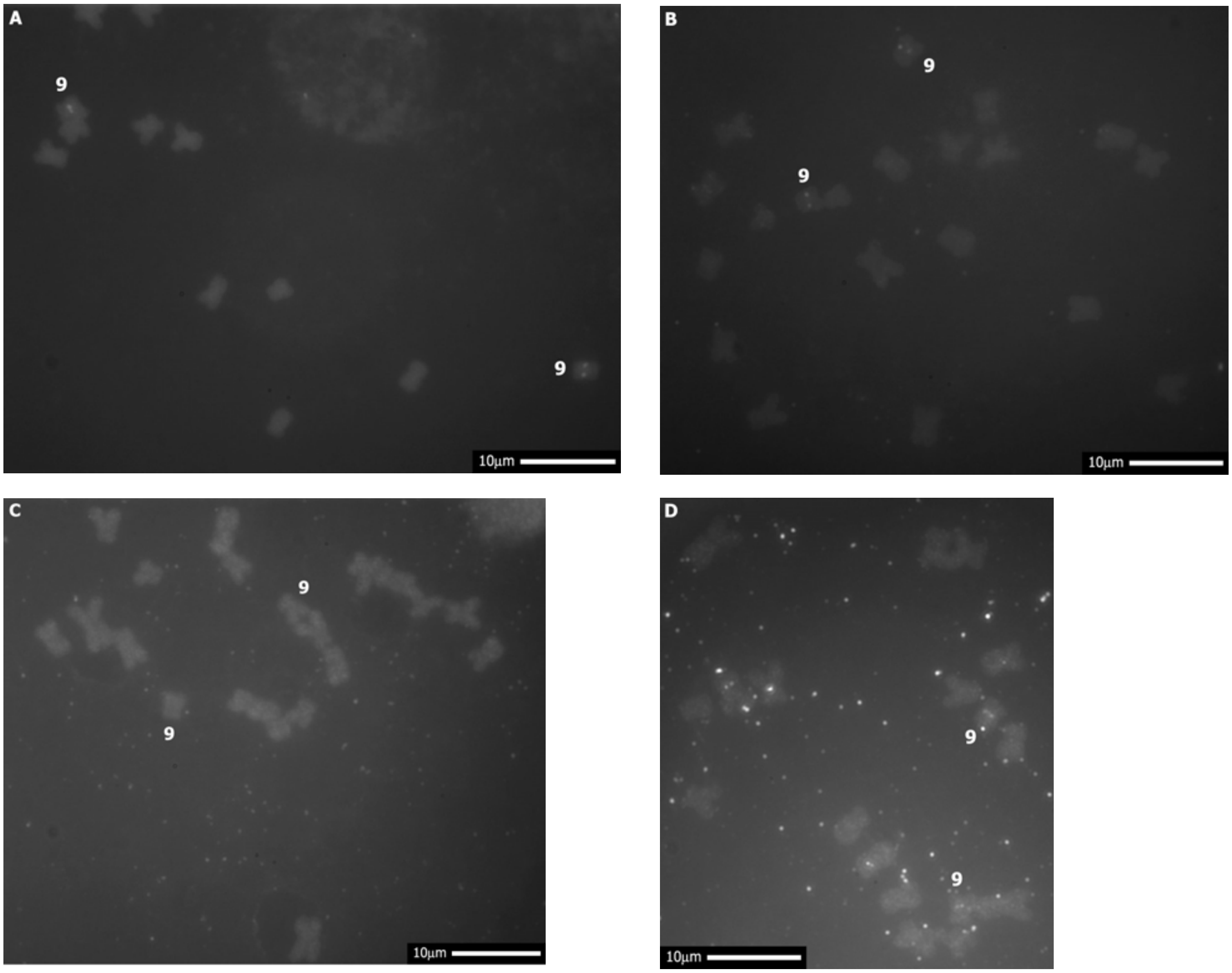

Figure S7 The 2.4- and 3.3-kb probes hybridized to the 9L NUMT in the B73 line. Only small portions of 2.4- and 3.3-kb DNA segments (≤ 82 bp) are found within the NB mitochondrial genome (Table 1); therefore, there is minimal background hybridization to the cytoplasmic mtDNA in the B73 line. Shown here are metaphase chromosome spreads hybridized to the indicated probes. (A) The Texas red-labeled 3.3-kb probe (white) hybridized to B73 chromosomes, including the 9L NUMT. Minimal background hybridization is detectable in B73. (B) The Texas red-labeled 2.4-kb probe (white) hybridized to B73 chromosomes, including the 9L NUMT. (C) In contrast, the Texas red-labeled 3.3-kb probe (white) did not hybridize to 9L in Ky21. The Ky21 line contains the NA mitochondrial genome, which includes the 3.3-kb segment. Therefore, the 3.3-kb probe hybridizes to mtDNA from ruptured mitochondria, creating hybridization signals in the cytoplasm. (D) Similar hybridization to cytoplasmic mtDNA can be observed with B73 when using a probe that is present in the NB mitochondrial genome. The Texas red-labeled

cosmid 2 probe (white) hybridized to both B73 chromosomal NUMTs and cytoplasmic mtDNA. Cosmid 2 contains ~31.7 kb of NB mtDNA. The karyotyping probes used to identify each chromosome are not shown. Scale = 10 μm.

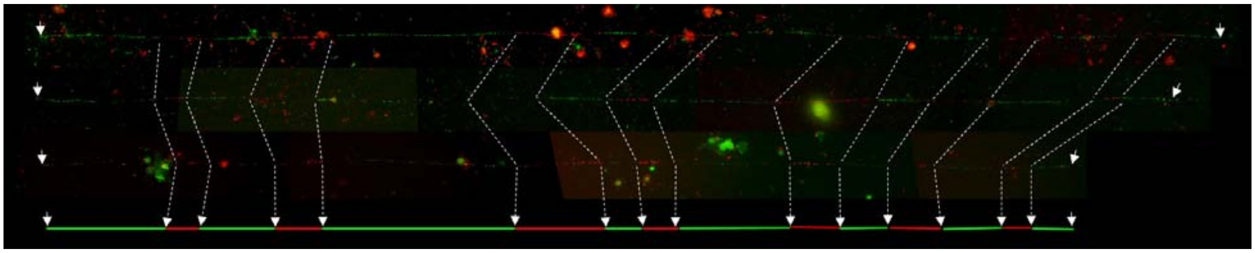

Figure S8 Measurement of the B73 9L NUMT using fiber-FISH. Five intact DNA fibers were measured; the three fibers compared in this figure were hybridized with two mtDNA labels: cosmids 1-7 (red), and cosmids 8-10, 16-18, and 20 (green). The solid red and green line at the bottom of the figure show the composite NUMT. The upper three pairs of arrows on either side of the figure indicate the start and end of the NUMT on the three separate fibers. The red and green colored sections in each fiber are different lengths due to different levels of stretching in the fibers. The alternating green and red labeling pattern among each fiber shows an identical pattern, indicating that each fiber is intact. Dashed lines and arrows that connect the DNA fibers to the solid line at the bottom designate the red and green labels hybridized to the fibers. Sizing was determined similarly to Figure 7. The size of the B73 9L NUMT is estimated to be ~1.8 Mb (1811.6 ± 229.3 kb, n = 5).

FILE S1

Supporting Materials and Methods

Diverse Maize Lines Examined: For this study, lines were chosen based on their designation in an association panel of 301 diverse maize inbred lines. This panel of lines was genotyped, and the underlying population substructure was established using 89 simple sequence repeat (SSR) loci (Flint-Garcia *et al*. 2005). Three main subgroups were identified: stiff stalk, non-stiff stalk, and tropical/subtropical. Because of their isolated breeding histories, sweet corn and popcorn lines formed their own distinct subgroups, and lines with mixed ancestry were classified as "mixed." From this large panel of inbred lines, 26 inbreds were chosen as founder lines to represent the diversity of maize in a population called the Nested Association Mapping (NAM) population (Yu *et al*. 2008). Each of the 16 lines examined in Figure 1 are NAM founder lines, with the exception of B37, Mo17, and M825.

M825 Pedigree Examined: Sweet corn distinguishes itself based on the presence of one or multiple recessive mutations that affect endosperm starch synthesis (Tracy 2001). The most popular mutations are *sugary1* (*su1*) and *shrunken2* (*sh2*), which both cause an increase of sugar in the endosperm and a decrease in starch content (Neuffer *et al*. 1997; Hannah 2005).

The earliest member of the M825 pedigree (Figure 4) included in this study is the sweet corn line P39. P39 carries the *su1* mutation (McMullen *et al*. 2009) but not *sh2* (Revilla *et al*. 2006; McMullen *et al*. 2009). According to the Maize Genetics and Genomics Database (MaizeGDB; Schaeffer *et al*. 2011), IP39 was produced from a P39 population by selecting for narrow kernels (Schaeffer *et al*. 2011). Ia5125 (also known as I5125 or 5125) was produced by crossing Tendermost onto IP39, then backcrossing by IP39 (Schaeffer *et al*. 2011). Ia5125 also carries *su1* (Ordás *et al*. 2006). Tendermost is a commercial sweet corn line (Wehner 2010) that is not currently available through the Germplasm Resources Information Network (GRIN; USDA 2011). The R825 line was produced from a cross of Ia5125 (*su1*/*su1 Sh2*/*Sh2*) by a *Su1*/*Su1 sh2*/*sh2* stock (stock from Mains 1949), and then repeatedly backcrossing the progeny to Ia5125 (S. Gabay-Laughnan, personal communication; Gerdes *et al*. 1993). R825 was later crossed by the line "Elite" (*sh2*/*sh2 Su1*/*Su1*) and then recurrently self-pollinated to form the line M825

sh2/*sh2 Su1*/*Su1* (S. Gabay-Laughnan, personal communication). Elite is an unavailable sweet corn line most likely made by crossing Golden Cross Bantam (*su1*/*su1*) by the *Su1*/*Su1 sh2*/*sh2* stock (Mains 1949) discussed above (S. Gabay-Laughnan, personal communication). M825 was then crossed onto Wf9 *Sh2*/*Sh2 A1*/*A1* and backcrossed to M825 for 10 generations, recurrently selecting for *Sh2* and the tightly linked *A1* locus (*anthocyaninless1*; S. Gabay-Laughnan, personal communication). Wf9 is a yellow dent line, not a sweet corn line (Gerdes *et al*. 1993; Schaeffer *et al*. 2011). The resulting sweet corn-derived M825 line has been self-pollinated since the backcrossing was completed (S. Gabay-Laughnan, personal communication). The final version of M825 was examined in this study.

Amplification of 2.4 and 3.3 kb Regions: The 2.4 kb region primers were designed based on the BAC AC183911 sequence (Table S2) using the program FastPCR (Kalendar *et al*. 2009). Two sets of 3.3 kb region primers were designed (Table S2) and used to produce PCR products for nick translation probe preparation procedures. The 3.3 kb region primers designed based on the BAC AC187467 were identified using the program FastPCR (Kalendar *et al*. 2009). The 3.3 kb region primers designed based on the BAC AC183911 were identified using the program Primer3 (Rozen and Skaletsky 2000). BAC DNA was isolated for amplification using the QIAGEN Plasmid plus midi kit (catalog number 12943, Valencia, CA).

Forward and reverse primers (Table S2) were used at a final concentration of 0.1 µM each in a PCR reaction with Promega GoTaq Green Master Mix (catalog number M712, Madison WI). Isolated BAC DNA for amplification of the regions was used at a final concentration of ~1 ng in a 100 µl PCR reaction. The PCR reaction mix was made of: 2 µl forward primer (10 µM), 2 µl reverse primer (10 µM), 2 µl isolated BAC DNA, 50 µl Promega GoTaq Green Master Mix, and 44 µl sterile water. The PCR parameters for the 2.4 kb and 3.3 kb regions were: 1) initial denaturation at 95° for 5 minutes; 2) denaturation at 95° for 30 seconds; 3) annealing at 55° for 30 seconds; 4) extension at 72° for 4 minutes; and 5) final extension at 72° for 8 minutes. Steps 2 - 4 were repeated for 40 cycles. The PCR products were purified using the Promega Wizard SV Gel and PCR Cleanup System Kit (catalog number A9282, Madison, WI).

FISH Methodology: The protocols used for preparing slides of root tip chromosome spreads, the labeling of both karyotyping and cosmid probes, and the capturing and processing of FISH images were previously described by Lough *et al*. (2008). Alterations to those methods are detailed below.

FISH Probes: The karyotyping probes used to identify chromosomes included eight regions of repetitive DNA common to most maize lines (Kato *et al*. 2004). These probes were labeled through nick translation with the fluorescence-labeled nucleotides: Cascade Blue-7-dUTP, Alexa Fluor 488-5-dUTP, or Cyanine 5-dUTP (Cy5). Mitochondrial DNA-containing cosmid probes were produced using segments of the NB maize mitochondrial genome and were labeled by nick translation with Texas red-5-dCTP (Lough *et al*. 2008). The NB mitochondrial genome was previously sequenced from the stiff stalk line B37 (Clifton *et al*. 2004) and is also present in B73. These mtDNA probes are either 20 individually labeled segments (Table S1) of the mitochondrial genome or a combination in a 19-cosmid mix probe (Figure 2A). Cosmid 13 is not included in this mix of 19 segments because it contains plastid DNA (Lough *et al*. 2008). The 2.4- and 3.3-kb probes were also labeled with Texas Red-5-dCTP.

Root Tip Mitotic Metaphase Chromosome Spread Slide Preparation: To obtain mitotic metaphase chromosomes for experiments, root tips were digested with cellulase and pectolyase, then rinsed with 1X TE and 100% ethanol (Lough *et al*. 2008). After removing the ethanol, 100% acetic acid was added to the tube, and the root tip was broken. This suspension was deposited on slides, and the slides were UV crosslinked (120-5 mJ/cm2). Formaldehyde was not used in the preparation or hybridization of slides.

Hybridization of FISH Slides: For experiments using the 19-cosmid probe, the mixed probes for each slide included 2.5 µl of the 19-cosmid mix (final concentration 200 ng/µl), 0.8 µl of 2X SSC/1X TE, and 1.7 µl of the mix of 8 karyotyping probes (Lough *et al*. 2008). The concentrations of the eight karyotyping probes varied (according to Table 1 in Lough *et al*. 2008). For experiments using the individual cosmid probes, the mixed probes for each slide included 1 µl of the labeled single cosmid probe (final concentration 20 ng/µl) and 4 µl of 8 karyotyping probe mix (with 2X SSC/1X TE); or 1 µl of the labeled single cosmid probe (final concentration 20 ng/µl), 0.35 µl Cent C probe (final concentration 3.5 ng/µl), 0.5

µl knob probe (final concentration 20 ng/µl), 0.5 µl 4-12-1 probe (final concentration 20 ng/µl), and 2.65 µl 2X SSC/1X TE. The latter mix was used to recognize chromosome 9 specifically. For experiments using the 2.4- and 3.3-kb probes, the mixed probes for each slide included 1 µl 2.4- or 3.3-kb probe (final concentration 40 ng/µl); 0.35 µl Cent C probe (final concentration 3.5 ng/µl); 0.5 µl knob probe (final concentration 20 ng/µl); 0.5 µl 4-12-1 probe (final concentration 20 ng/µl); and 2.65 µl 2X SSC/1X TE. After the hybridization and washing of the slides, the slides were mounted using Vectashield (catalog number NC9524612 through Fisher Scientific, Vector Laboratories, Burlingame, CA) that contained 4',6-diamidino- 2-phenylindole (DAPI).

Pachytene FISH Methodology: Pachytene slides were made from B73 anthers. Tassels were collected and placed in a 3:1 solution of ethanol and acetic acid for 24 - 48 hours while stored at 4°. The tassels were rinsed in 70% ethanol and stored in -20°. Individual anthers were examined for pachytene chromosomes using acetocarmine staining. A single anther was placed on a slide in a drop of acetocarmine and then smashed using a dissecting needle. A coverslip was put over the smashed anther, and the slide was gently heated over a flame. When pachytene chromosomes were identified, the remaining anthers in that area of the tassel were prepared similar to the root tip slide preparations. After an enzyme digestion, the anthers were rinsed once using TE and three times using 100% ethanol. The anthers were broken in a 3:1 solution of acetic acid and methanol. The resulting solution was dropped onto slides and slides were UV crosslinked.

The BAC-specific (BAC-L1) and *glossy15* (*gl15*) FISH probes were made as described in Danilova and Birchler (2008). Both the BAC-specific and *gl15* probe were prepared using the fluorescence-labeled nucleotide Alexa Fluor 488-5-dUTP. Slides were hybridized using the same procedure as root tip slides. Each of the following probes were used per slide: 0.64 µl Alexa Fluor 488-labeled BAC-specific probe (200 ng/µl), 0.37 µl Alexa Fluor 488-labeled *gl15* probe (200 ng/µl), 1.0 µl Texas Red-labeled cosmid 3 probe (100 ng/µl), 1.0 µl Texas Red-labeled cosmid 9 probe (100 ng/µl), and 1.99 µl 2X SSC/1X TE.

Fiber-FISH Methodology: The fiber-FISH procedures used for preparing slides and labels, and hybridizing slides in this study were defined previously by Koo *et al*. (2011). To detect mtDNA insertions on B73 DNA fibers, different combinations of two-color fiber-FISH procedure were used, as described in Jackson *et al*. (1998). The first combination included only labels of mtDNA: digoxigenin labeled probe A (cosmids 1-7) detected as red and biotin labeled probe B (cosmids 8-10, 16-18, and 20) detected as green. The second combination included labels of mtDNA and 5-methylcytosine. The mtDNA was detected using a biotin-16-dUTP (catalog number 11093070910, Roche, Indianapolis, IN) labeled probe for 19 of the 20 available overlapping sections of the 570-kb NB maize mitochondrial genome cloned into cosmid vectors (Lough *et al*. 2008). Cosmid 13 was omitted because it contains a large amount of plastid DNA. After hybridization, the slides were washed, and the mtDNA label was detected with goat anti-biotin antibody (catalog number SP-3000, Vector Laboratories, Burlingame, CA) and then rhodamine-conjugated donkey anti-goat antibody (catalog number 705-296-147, Jackson ImmunoResearch, West Grove, PA) was used to stain (Koo *et al*. 2011). For the detection of 5-methylcytosine, slides were incubated with mouse antiserum that was raised against 5-methylcytosine (catalog number MABE146, Millipore, Massachusetts); these antibodies were subsequently exposed to rabbit anti-mouse Alexa Fluor 488 antibody (catalog number A27023, Invitrogen, Grand Island, NY). When measuring the 9L NUMT DNA fibers, red and green colored sections derived from each fiber showed different lengths, reflecting the different stretching degrees in individual fibers. However, the alternative green and red labeling pattern among individual DNA fibers showed an identical pattern, indicating intact fibers. Five intact fibers were measured: one fiber with mtDNA and methylation labels and four fibers with only mtDNA labels (Figure S8). The cytological measurements of the fiber-FISH signals were converted into kilobases using a 3 kb per µm conversion rate (Cheng *et al.* 2002).

Sequence Analysis of the B73 Chromosome 9L NUMT: The maize NB mitochondrial genome was compared to the maize nuclear reference genome assembly version 2 using the ZeAlign program available through MaizeGDB (Sen *et al*. 2010). The NB mitochondrial genome was used for these comparisons because B73 contains this mitochondrial genome and because the FISH experiments used probes for this genome. The resulting files from this comparison were uploaded as a custom track on the

maize genome browser at MaizeGDB to visualize the mtDNA insertions relative to the chromosome 9 centromere, BACs, and the gene models (Sen *et al*. 2010). The gene models displayed on the MaizeGDB genome browser were identified by the Maize Sequencing Consortium release 5b.60 (Sen *et al*. 2010). Retrotransposons were detected using the program RepeatMasker (Smit *et al*. 1996-2010). The annotated gene models were given a functional identification based on the presence of a known mitochondrial gene in that location (NCBI Accession AY506529.1; Clifton *et al*. 2004) and InterProScan results, which identify protein domains (Zdobnov and Apweiler 2001).

Table S1 Segments of the NB mitochondrial genome within cosmids.

Cosmid	NB Mitochondrial Start Position (bp)	NB Mitochondrial End Position (bp)
1	560671	28913
2	11115	42912
3	40337	75268
4	65967	103378
5	92547	132562
6	128405	164326
7	158622	195116
8	182577	220863
9	205820	244602
10	239824	273611
11	254638	295072
12	283267	319446
13	312072	351606
14	341466	378752
15	377800	416027
16	411027	447039
17	442367	473713
18	458736	487130
19	487000	520351
20	522351	5100

The base-pair positions of the NB mtDNA represented within each cosmid were estimated from the restriction endonuclease maps (Fauron *et al.* 1987; Fauron and Havlik 1988) compared to the NB mitochondrial genome sequence (NCBI Accession AY506529.1; Clifton *et al*. 2004) using SeqBuilder™ (DNASTAR, Madison, WI).

Table S2 Primers used to amplify the 2.4 and 3.3 kb regions from BAC DNA.

Region	BAC	Product Size (bp)	Primer Name	Tm (°C)	Primer Sequence
2.4 kb	AC183911	2639	4F1_331-354	59.2	GCTGAAGTAGCCTAAGCGCTTCAA
			6R1_2948-2969	56.7	CTGCCAACTTGTATGCATCACC
3.3 kb	AC187467	3704	6F1_243-266	60.4	ACATCAGCTCAACCTTCTTGCCCA
			2R1_3925-3946	59.3	TGGCTACGAACCACGATGTTGG
3.3 kb	AC183911	3968	AL183911-1F	59.8	ATAGACGGAGGAATGGATACGA
			AL183911-1R	59.7	ACCGTAGAACAAATTCGAGAGG

Table S3 Locations of retrotransposons in the B73 chromosome 9L NUMT region.

Retrotransposon Sequence[a]	Type	Length (bp)	Nuclear Start Position (bp)	Nuclear End Position (bp)
Gypsy29-ZM_I-int[b]	LTR/Gypsy	254	72725993	72726247
Gypsy-175_ZM-LTR	LTR/Gypsy	721	72736759	72737480
Gypsy-175_ZM-I	LTR/Gypsy	823	72737481	72738304
Gypsy-174_ZM-I	LTR/Gypsy	4963	72738297	72743260
Gypsy-175_ZM-LTR	LTR/Gypsy	584	72743261	72743845
Gypsy-127_ZM-I	LTR/Gypsy	288	72746025	72746313
PREM1A_ZM_LTR	LTR/Copia	224	72746314	72746538
Gypsy-127_ZM-I	LTR/Gypsy	53	72746539	72746592
Gypsy-127_ZM-I	LTR/Gypsy	1850	72747177	72749027
Gypsy-127_ZM-LTR	LTR/Gypsy	252	72749028	72749280
Gypsy-188_ZM-LTR	LTR/Gypsy	669	72768138	72768807
Gypsy-175_ZM-I	LTR/Gypsy	6090	72768747	72774837
Gypsy-188_ZM-LTR	LTR/Gypsy	669	72774838	72775507
Gypsy-109_ZM-I[b]	LTR/Gypsy	447	72803659	72804106
LINE1-12_ZM[b]	LINE/L1	98	72811368	72811466
Gypsy-175_ZM-I	LTR/Gypsy	4812	72820412	72825224
Gypsy-188_ZM-I	LTR/Gypsy	4354	72822853	72827207
ZEON2_ZM_LTR	LTR/Gypsy	663	72827208	72827871
LINE1-50_ZM[b]	LINE/L1	101	72838354	72838455
HOPSCOTCH_ZM_I-int[b]	LTR/Copia	1162	72850584	72851746
Copia33-ZM_I-int[b]	LTR/Copia	57	72875960	72876017
Gypsy-100_ZM-I[b]	LTR/Gypsy	108	72889185	72889293
Gypsy-198_ZM-I[b]	LTR/Gypsy	128	72900074	72900202
Gypsy-127_ZM-LTR	LTR/Gypsy	252	72951840	72952092
Gypsy-127_ZM-I	LTR/Gypsy	1850	72952093	72953943
Gypsy-127_ZM-I	LTR/Gypsy	53	72954528	72954581
PREM1A_ZM_LTR	LTR/Copia	224	72954582	72954806
Gypsy-127_ZM-I	LTR/Gypsy	2884	72954807	72957691
Gypsy-174_ZM-I	LTR/Gypsy	933	72957792	72958725
Gypsy-175_ZM-LTR	LTR/Gypsy	721	72958726	72959447
Gypsy-174_ZM-I	LTR/Gypsy	3869	72959448	72963317
Gypsy-174_ZM-I	LTR/Gypsy	1880	72963434	72965314
Gypsy-175_ZM-I	LTR/Gypsy	824	72965307	72966131
Gypsy-175_ZM-I	LTR/Gypsy	253	72966131	72966384
Gypsy-188_ZM-LTR	LTR/Gypsy	669	72967168	72967837
Gypsy-175_ZM-I	LTR/Gypsy	6090	72967777	72973867
Gypsy-188_ZM-LTR	LTR/Gypsy	669	72973868	72974537
ZDE_ZD	LTR/Gypsy	483	72975345	72975828
ZDE_ZD	LTR/Gypsy	56	72975822	72975878
ZDE_ZD	LTR/Gypsy	105	72975931	72976036
Gypsy-127_ZM-LTR	LTR/Gypsy	435	72976070	72976505
PREM1_ZM	LTR/Copia	514	72976506	72977020
Gypsy-127_ZM-LTR	LTR/Gypsy	51	72977021	72977072
Gypsy-127_ZM-I	LTR/Gypsy	487	72977205	72977692
Gypsy-175_ZM-LTR	LTR/Gypsy	584	72979872	72980456
Gypsy29-ZM_I-int[b]	LTR/Gypsy	254	72990963	72991217

[a]The retrotransposons were identified using the program RepeatMasker (Smit *et al*. 1996-2010).
[b]These retrotransposon fragments overlap with sections of NB mtDNA (Table 1).

Retrotransposons present among the mitochondrial sequences within the 9L NUMT (Table 1) include those whose LTRs have 100% identity.

References

Candela H., and S. Hake, 2008 The art and design of genetic screens: maize. Nat. Rev. Genet. 9: 192-203.

Cheng, Z. K., C. R. Buell, R. A. Wing, and J. M. Jiang, 2002 Resolution of fluorescence in situ hybridization mapping on rice mitotic prometaphase chromosomes, meiotic pachytene chromosomes and extended DNA fibers. Chromosome Res. 10: 379-387.

Clifton S. W., P. Minx, C. M.-R. Fauron, M. Gibson, J. O. Allen, *et al.*, 2004 Sequence and comparative analysis of the maize NB mitochondrial genome. Plant Physiol. 136: 3486-3503.

Danilova T. V. and J. A. Birchler, 2008 Integrated cytogenetic map of mitotic metaphase chromosome 9 of maize: resolution, sensitivity, and banding paint development. Chromosoma 117: 345-356.

Fauron C. M.-R., A. G. Abbott, R. I. S. Brettell, R. F. Gesteland, 1987 Maize mitochondrial DNA rearrangements between the normal type, the Texas male sterile cytoplasm, and a fertile revertant cms-T regenerated plant. Curr. Genet. 11: 339-346.

Fauron C. M.-R. and M. Havlik, 1988 The BamHI, XhoI, SmaI restriction enzyme maps of the normal maize mitochondrial genome genotype B37. Nucleic Acids Res. 16: 10395-10396.

Flint-Garcia S. A., A. C. Thuillet, J. Yu, G. Pressoi, S. M. Romero, *et al.*, 2005 Maize association population: a high-resolution platform for quantitative trait locus dissection. Plant J. 44: 1054-1064.

Gerdes J. T., C. F. Behr, J. G. Coors, and W. F. Tracy, 1993 *Compilation of North American Maize Breeding Germplasm*. Crop Science Society of America, Madison.

Hannah L. C., 2005 Starch synthesis in the maize endosperm. Maydica 50: 497-506.

Jackson S. A., M. L. Wang, H. M. Goodman, and J. Jiang, 1998 Application of fiber-FISH in physical mapping of *Arabidopsis thaliana*. Genome 41: 566-572.

Kalendar R., D. Lee, and A. H. Schulman, 2009 FastPCR software for PCR primer and probe design and repeat search. Genes, Genomes and Genomics 3: 1-14.

Kato A., J. C. Lamb, and J. A. Birchler, 2004 Chromosome painting using repetitive DNA sequences as probes for somatic chromosome identification in maize. Proc. Natl. Acad. Sci. U. S. A. 101: 13554-13559.

Koo D.-H., F. Han, J. A. Birchler, and J. Jiang, 2011 Distinct DNA methylation patterns associated with active and inactive centromeres of the maize B chromosome. Genome Res. 21: 908-914.

Lausser A., I. Kliwer, K.-o. Srilunchang, and T. Dresselhaus, 2010 Sporophytic control of pollen tube growth and guidance in maize. J. Exp. Botany 61: 673-682.

Lee M., N. Sharopova, W. D. Beavis, D. Grant, M. Katt, *et al.*, 2002 Expanding the genetic map of maize with the intermated B73 x Mo17 (IBM) population. Plant Mol. Biol. 48: 453-461.

Lough A. N., L. M. Roark, A. Kato, T. S. Ream, J. C. Lamb, *et al.*, 2008 Mitochondrial DNA transfer to the nucleus generates extensive insertion site variation in maize. Genetics 178: 47-55.

Mains E. B., 1949 Heritable characters in maize: Linkage of a factor for shrunken endosperm with the *a1* factor for aleurone color. J. Hered. 40: 21-24.

McWilliam H., W. Li, M. Uludag, S. Squizzato, Y. M. Park, *et al.*, 2013 Analysis tool web services from the EMBL-EBI. Nucleic Acids Res. 41: W597-600.

McMullen M. D., S. Kresovich, H. S. Villeda, P. Bradbury, H. Li, *et al.*, 2009 Genetic properties of the maize nested association mapping population. Science 325: 737-740.

Neuffer M. G., E. Coe, and S. R. Wessler, 1997 *The Mutants of Maize*. Cold Spring Harbor Laboratory Press, Cold Spring Harbor.

Ordás B., G. Padilla, R. A. Malvar, A. Ordás, V. M. RodrÍguez, *et al.*, 2006 Cold tolerance improvement of *sugary enhancer1* hybrids of sweet corn. Maydica 51: 567-574.

Revilla P., R. A. Malvar, V. M. RodrÍguez , A. Butrón, B. Ordás, *et al.*, 2006 Variation of *sugary1* and *shrunken2* gene frequency in different maize genetic backgrounds. Plant Breeding 125: 478-481.

Rozen S., and H. J. Skaletsky, 2000 Primer3 on the WWW for general users and for biologist programmers, pp. 365-386 in *Bioinformatics Methods and Protocols: Methods in Molecular Biology*, edited by S. Krawetz and S. Misener. NJ Humana Press, Totowa.

Schaeffer M. L., L. C. Harper, J. M. Gardiner, C. M. Andorf, D. A. Campbell, *et al.*, 2011 MaizeGDB: curation and outreach go hand-in-hand. Database 2011: bar022.

Sen T. Z., L. C. Harper, M. L. Schaeffer, C. M. Andorf, T. Seigfried, *et al.*, 2010 Choosing a genome browser for a Model Organism Database: surveying the Maize community. Database 2010: baq007.

Smit A. F. A., R. Hubley, and P. Green, 1996-2010 RepeatMasker Open-3.0. (http://www.repeatmasker.org)

Tracy W. F., 2001 Sweet Corn, pp. 155-198 in *Specialty Corns*, edited by A. R. Hallauer. CRC Press LLC, Boca Raton.

USDA, ARS, National Genetic Resources Program. *Germplasm Resources Information Network - (GRIN)*. [Online Database] National Germplasm Resources Laboratory, Beltsville, Maryland. (http://www.ars-grin.gov/cgi-bin/npgs/html/search.pl?NSL+32778)

Wehner T. C., 2010 “Vegetable Cultivar Descriptions for North America: Sweetcorn (M-Z), Lists 1-26 Combined”. In: Tracy WF, eds. *Cucurbit Breeding at NC State*. North Carolina State University, Raleigh. Retrieved from (http://cuke.hort.ncsu.edu/cucurbit/wehner/vegcult/sweetcornmz.html)

Yu J., J. B. Holland, M. D. McMullen, and E. S. Buckler, 2008 Genetic design and statistical power of nested association mapping in maize. Genetics 178: 539-551.

Ziegler K. E., 2001 Popcorn, pp. 199-234 in *Specialty Corns*, edited by A. R. Hallauer. CRC Press LLC, Boca Raton.

Zdobnov E. M., and R. Apweiler, 2001 InterProScan - an integration platform for the signature-recognition methods in InterPro. Bioinformatics 17: 847-848.
