# Supplementary material for: Cytogenetic and Sequence Analyses of Mitochondrial DNA Insertions in Nuclear Chromosomes of Maize
Source: G3 (Bethesda). 2015 Sep 1;5(11):2229–39. doi: 10.1534/g3.115.020677 (PMC4632043; doi:10.1534/g3.115.020677)
Supplement: Supporting Information [file supp_g3.115.020677_FigureS1.pdf]

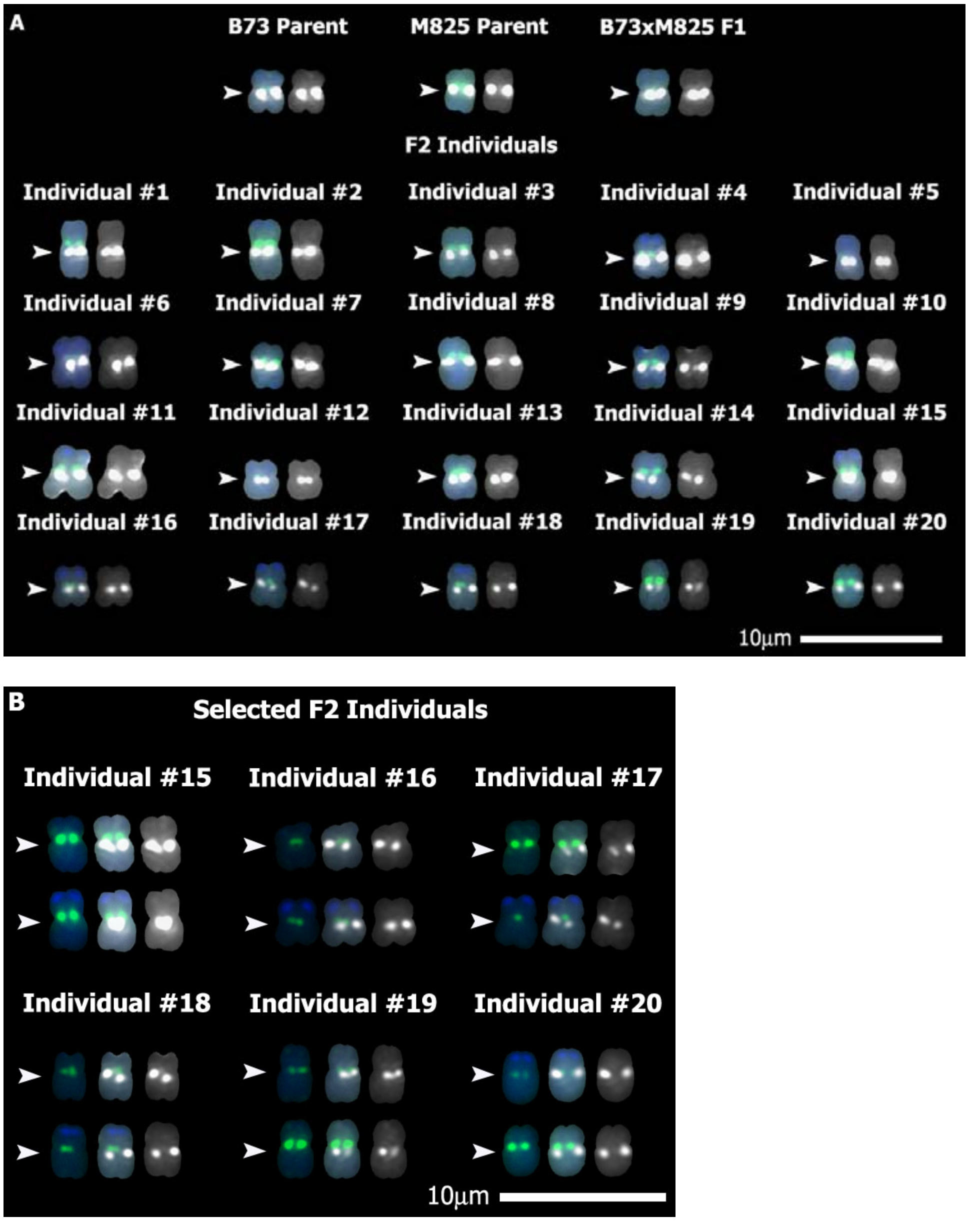

Figure S1 Test for recombination between the 9L NUMT in B73 and M825. F2 individuals from a self-pollination of a B73/M825 F1 hybrid were used in this study. If the NUMTs are located at different sites on chromosome 9L, then recombination could occur and chromosomes containing no NUMT or a doubly large NUMT would be found. At this level of resolution, there is no evidence of the recombination event. In the images shown here, cosmids 7 and 8 were used as probes because both were observed at the chromosome 9L NUMT of B73 and M825 with a clearly visible signal (Figure 2B). Initial concentrations are indicated for each probe with the volume used. Texas red-labeled probes of cosmids 7 and 8 (1 μl

each at 100 ng/μl) were used on slides of the first 20 F2 individuals examined. To identify chromosome 9 specifically, three karyotyping probes were used per slide: 0.35 μl Alexa Fluor 488-labeled Cent C (50 ng/μl), 0.5 μl Cascade Blue-labeled knob (200 ng/μl), and 0.5 μl Alexa Fluor 488-labeled 4-12-1 (200 ng/μl). The cosmid and karyotyping probes were combined with 1.65 μl 2X SSC /1X TE per slide for a total of 5 μl probe mix added to each slide. After the first 20 F2 individuals were examined, 1 μl each of the 2.4- and 3.3-kb probes (200 ng/μl) were used on an additional 26 individuals, instead of cosmid 7 and 8 probes. A minimum of 10 chromosomes was observed from each individual root tip examined; a total of 8 chromosomes were observed for the B73, M825, and B73 x M825 hybrid. (A) Representative chromosomes for B73, M825, the B73 x M825 hybrid, and the first 20 F2 B73/M825 selfed chromosomes examined using cosmid 7 and 8 probes are shown. The chromosome to the left shows both the karyotyping probe (color) and mtDNA probe (white) layers while the chromosome on the right shows only the mtDNA probe layer. A white arrowhead indicates the position of the NUMT. Scale = 10 μm. (B) Both chromosomes 9 from a single cell are shown for individuals 15-20 to illustrate the similar probe signal strength present on both chromosomes. Three views of each homolog originating from a single cell are shown for all six individuals. The first view of the homologs (left) shows only the karyotyping probe layer (color), the second view of the homologs (middle) shows both the karyotyping probe (color) and mtDNA probe (white) layers, and the third view of the homologs (right) shows only the mtDNA probe layer (white). The white arrowheads indicate the presence of a 9L NUMT. Scale = 10 μm.
