# Supplementary material for: Cytogenetic and Sequence Analyses of Mitochondrial DNA Insertions in Nuclear Chromosomes of Maize
Source: G3 (Bethesda). 2015 Sep 1;5(11):2229–39. doi: 10.1534/g3.115.020677 (PMC4632043; doi:10.1534/g3.115.020677)
Supplement: Supporting Information [file supp_g3.115.020677_FigureS2.pdf]

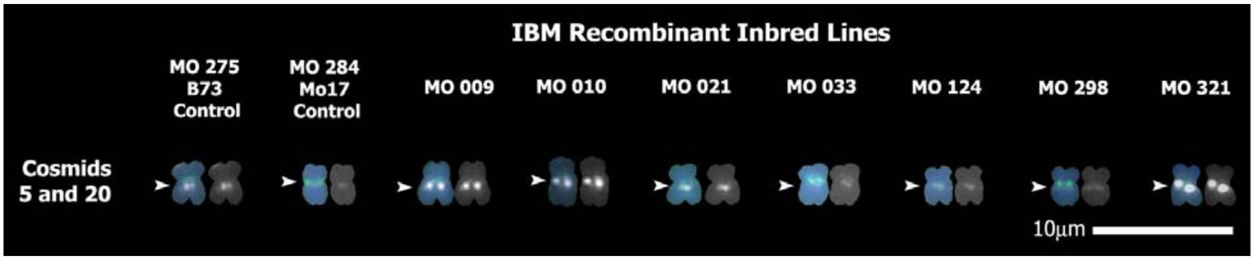

Figure S2 Test for recombination between the 9L NUMT in B73 and Mo17 using recombinant inbred lines derived from a B73 x Mo17 F1 hybrid. The Intermated B73 x Mo17 (IBM) recombinant inbred lines (RILs) are commonly used as a mapping tool in maize (Lee *et al*. 2002; Candela and Hake 2008). The IBM lines were created by first making hybrids of B73 and Mo17, self-pollinating the F1 plants for one generation, cross-pollinating their progeny for four generations, and finally self-pollinating the plants for multiple generations (Candela and Hake 2008). The resulting IBM RILs are homozygous at all loci and show alternating blocks of recombined parental DNA throughout the genome. Previous pachytene FISH experiments indicated that the B73 9L NUMT was located between the centromere and the BAC-specific and *glossy15* (*gl15*) probes (Figure S4). The closest IBM marker to *gl15* is umc1691 (244.10 cM). Data from K. Dawe's lab (personal communication) placed the chromosome 9 centromere below the IBM marker umc81 (226.30 cM). The range from 226.30 – 244.10 cM was extended to 219.40 – 251.80 cM (marker umc2338 to marker umc1700) to ensure that the strong signal 9L NUMT was included in the examined area. IBM RILs containing recombination between the markers umc2338 and umc1700 were selected. Initial concentrations are indicated for each probe with the volume used. Texas red-labeled cosmid 5 and 20 probes (1 µl each at 100 ng/µl) were used on every slide. These two cosmid probes were chosen because they are the only cosmid probes that were observed at the Mo17 9L NUMT (Figure 2B). To identify chromosome 9, three karyotyping probes were used per slide: 0.35 µl Alexa Fluor 488-labeled Cent C (50 ng/µl), 1 µl Cascade Blue-labeled knob (200 ng/µl), and 1 µl Alexa Fluor 488-labeled 4-12-1 (200 ng/µl). The cosmid and karyotyping probes were combined with 0.65 µl 2X SSC /1X TE per slide for a total of 5 µl probe mix added to each slide. A minimum of 17 chromosomes was observed from at least 2 individual root tips for each RIL examined. If the NUMTs are located at different sites on chromosome 9L, then recombination could occur and chromosomes containing no NUMT would be found. At this level of resolution, there is no evidence of the recombination event. Scale = 10 µm.
