# Supplementary material for: Cytogenetic and Sequence Analyses of Mitochondrial DNA Insertions in Nuclear Chromosomes of Maize
Source: G3 (Bethesda). 2015 Sep 1;5(11):2229–39. doi: 10.1534/g3.115.020677 (PMC4632043; doi:10.1534/g3.115.020677)
Supplement: Supporting Information [file supp_g3.115.020677_FigureS3.pdf]

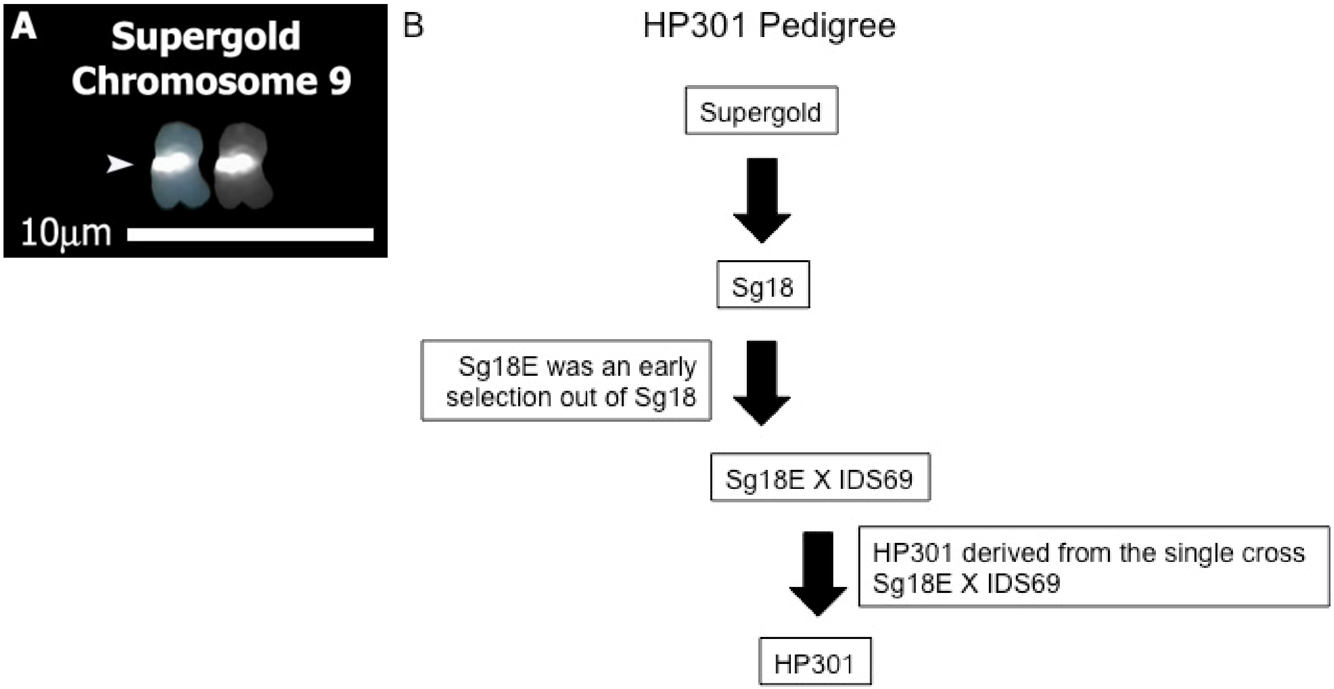

Figure S3 The 9L NUMT HP301 is present in the progenitor line Supergold. Supergold is a popcorn landrace that is a progenitor of HP301. Popcorns are a distinctive maize type in part because most popcorn lines contain a gametophytic incompatibility gene *Ga1*, which causes them to reject other types of pollen (Ziegler 2001; Lausser *et al*. 2010). (A) The Texas red-labeled 19-cosmid mix probe was used to examine Supergold and detected a relatively large NUMT on 9L near the centromere. This finding suggests that the HP301 9L NUMT was a descendent from the Supergold progenitor. Only chromosome 9 is shown. Sites of mtDNA hybridization are shown in white. Chromosomes were identified using a mix of eight karyotyping probes (shown in color). Chromosome on left: karyotyping probes and mtDNA probes. Chromosome on right: mtDNA probes only. White arrowhead indicates mtDNA insertions. Scale = 10 µm. (B) Pedigree of the popcorn line HP301. Listed here are the members of the HP301 pedigree, the popcorn line examined. Sg18E has not been maintained (M. Robbins and R.B. Ashman, personal communication). IDS69 is a South American line. The Supergold line examined in part (A) is one of the Supergold lines maintained by the Germplasm Resources Information Network (GRIN).
