# Supplementary material for: Cytogenetic and Sequence Analyses of Mitochondrial DNA Insertions in Nuclear Chromosomes of Maize
Source: G3 (Bethesda). 2015 Sep 1;5(11):2229–39. doi: 10.1534/g3.115.020677 (PMC4632043; doi:10.1534/g3.115.020677)
Supplement: Supporting Information [file supp_g3.115.020677_FigureS4.pdf]

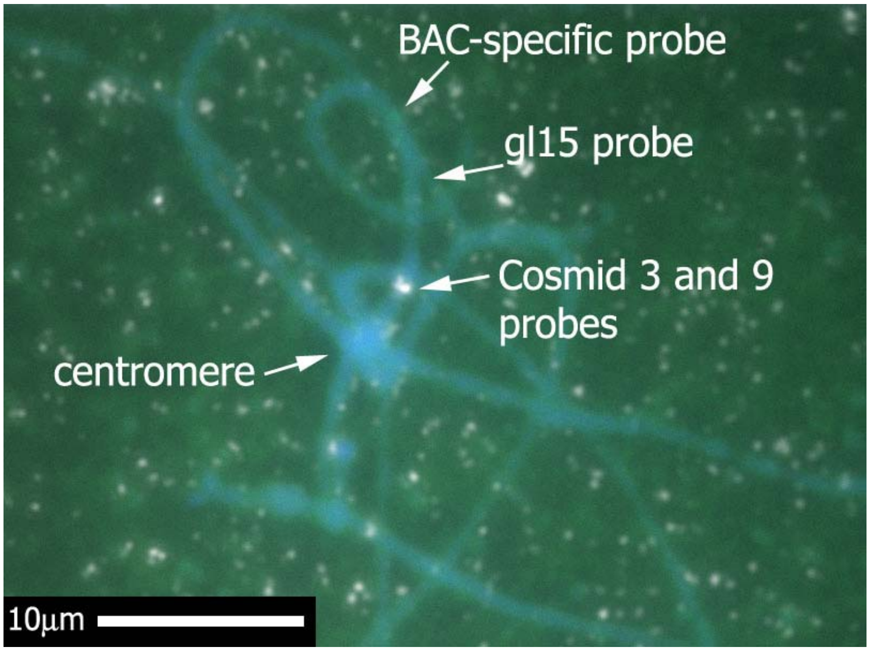

Figure S4 The B73 9L NUMT is located between the centromere and 9L-specific FISH probes on pachytene chromosomes. Chromosome 9 has been well-characterized cytogenetically with a series of probes on metaphase and pachytene chromosomes (Danilova and Birchler 2008). In the previous study, two proximal probes on the chromosome 9 long arm were BAC-specific probe (BAC-L1) and *glossy15* (*gl15*). B73 chromosomes were hybridized with Alexa Fluor 488-labeled BAC-specific and gl15 probes and Texas red-labeled cosmid 3 and 9 probes. Using these probes, the B73 chromosome 9L NUMT was identified between the 9L-specific probes and the centromere.
