# Supplementary material for: Cytogenetic and Sequence Analyses of Mitochondrial DNA Insertions in Nuclear Chromosomes of Maize
Source: G3 (Bethesda). 2015 Sep 1;5(11):2229–39. doi: 10.1534/g3.115.020677 (PMC4632043; doi:10.1534/g3.115.020677)
Supplement: Supporting Information [file supp_g3.115.020677_FigureS5.pdf]

```
NUMT_1          CATGCTTGTGATTGGCATGTTCCGAGAAGAGCACGGCTAAGGGCAAAGCAGACAAGGCAC 60
NUMT_2          CATGCTTGTGATTGGCATGTTCCGAGAAGAGCACGGCTAAGGGCAAAGCAGACAAGGCAC 60
NA              CATGCTTGTGATTGGCATGTTCCGAGAAGAGCACGGCTAAGGGCAAAGCAGACAAGGCAC 60
Zmp             CATGCTTGTGATTGGCATGTTCCGAGAAGAGCACGGCTAAGGGCAAAGCAGACAAGGCAC 60
                ************************************************************

NUMT_1          GGGTCCTATCCGCGGATTCGCAATAGTTCTAGTTCCCCGAAAGGACATCGTTCGCTTGCG 120
NUMT_2          GGGTCCTATCCGCGGATTCGCAATAGTTCTAGTTCCCCGAAAGGACATCGTTCGCTTGCG 120
NA              GGGTCCTATCCGCGGATTCGCAATAGTTCTAGTTCCCCGAAAGGACATCGTTCGCTTGCG 120
Zmp             GGGTCCTATCCGCGGATTCGCAATAGTTCTAGTTCCCCGAAAGGACATCGTTCGCTTGCG 120
                ************************************************************

NUMT_1          GCTGAAGTGGAGATACGGCAGTAGTTCCTATCCGGTTTTCAACGGCTAAGGGTCGGTCCA 180
NUMT_2          GCTGAAGTGGAGATACGGCAGTAGTTCCTATCCGGTTTTCAACGGCTAAGGGTCGGTCCA 180
NA              GCTGAAGTGGAGATACGGCAGTAGTTCCTATCCGGTTTTCAACGGCTAAGGGTCGGTCCA 180
Zmp             GCTGAAGTGGAGATACGGCAGTAGTTCCTATCCGGTTTTCAACGGCTAAGGGTCGGTCCA 180
                ************************************************************

NUMT_1          TAGAACCCTCGGTTTCGGCTTTCCTAGTCGGCTTTTCCACGAGTTGGATCGTGATGATCG 240
NUMT_2          TAGAACCCTCGGTTTCGGCTTTCCTAGTCGGCTTTTCCACGAGTTGGATCGTGATGATCG 240
NA              TAGAACCCTCGGTTTCGGCTTTCCTAGTCGGCTTTTCCACGAGTTGGATCGTGATGATCG 240
Zmp             TAGAACCCTCGGTTTCGGCTTTCCTAGTCGGCTTTTCCACGAGTTGGATCGTGATGATCG 240
                ************************************************************

NUMT_1          GAACTGATTCCTTTCCTTCCTATCCAAATACCGGAAACGGCCTTCCTATTTGGAAAATCC 300
NUMT_2          GAACTGATTCCTTTCCTTCCTATCCAAATACCGGAAACGGCCTTCCTATTTGGAAAATCC 300
NA              GAACTGATTCCTTTCCTTCCTATCCAAATACCGGAAACGGCCTTCCTATTTGGAAAATCC 300
Zmp             GAACTGATTCCTTTCCTTCCTATCCAAATACCGGAAACGGCCTTCCTATTTGGAAAATCC 300
                ************************************************************

NUMT_1          TATCCTGGTTCTGTTTCTGTGTAAGCAACTATTGCCTTACTTGAGGATGAGTTTTCCCCT 360
NUMT_2          TATCCTGGTTCTGTTTCTGTGTAAGCAACTATTGCCTTACTTGAGGATGAGTTTTCCCCT 360
NA              TATCCTGGTTCTGTTTCTGTGTAAGCAACTATTGCCTTACTTGAGGATGAGTTTTCCCCT 360
Zmp             TATCCTGGTTCTGTTTCTGTGTAAGCAACTATTGCCTTACTTGAGGATGAGTTTTCCCCT 360
                ************************************************************

NUMT_1          TGCTTTGCAACAACCAAATTCCCTTTTCTTCCAAGTTTCCAAATTCGTACTTCTGCAAAG 420
NUMT_2          TGCTTTGCAACAACCAAATTCCCTTTTCTTCCAAGTTTCCAAATTCGTACTTCTGCAAAG 420
NA              TGCTTTGCAACAACCAAATTCCCTTTTCTTCCAAGTTTCCAAATTCGTACTTCTGCAAAG 420
Zmp             TGCTTTGCAACAACCAAATTCCCTTTTCTTCCAAGTTTCCAAATTCGTACTTCTGCAAAG 420
                ************************************************************

NUMT_1          CTTTAGGGGTAGGGGTTTAGAGAAAGGGTCTTTCTTTCTACCTATGACGAATAACTTCTT 480
NUMT_2          CTTTAGGGGTAGGGGTTTAGAGAAAGGGTCTTTCTTTCTACCTATGACGAATAACTTCTT 480
NA              CTTTAGGGGTAGGGGTTTAGAGAAAGGGTCTTTCTTTCTACCTATGACGAATAACTTCTT 480
Zmp             CTTTAGGGGTAGGGGTTTAGAGAAAGGGTCTTTCTTTCTACCTATGACGAATAACTTCTT 480
                ************************************************************

NUMT_1          ATTATCATGGCGACAGGCGTTGTAAACCTACTTTTCCTACCTCCAGATGAGGAGGCCTTA 540
NUMT_2          ATTATCATGGCGACAGGCGTTGTAAACCTACTTTTCCTACCTCCAGATGAGGAGGCCTTA 540
NA              ATTATCATGGCGACAGGCGTTGTAAACCTACTTTTCCTACCTCCAGATGAGGAGGCCTTA 540
Zmp             ATTATCATGGCGACAGGCGTTGTAAACCTACTTTTCCTACCTCCAGATGAGGAGGCCTTA 540
                ************************************************************

NUMT_1          GGACAAACATTCCGAAGTCACTGGAACGGCAGTGATGTCATCCTGTGTCAAAGTAGAGCA 600
NUMT_2          GGACAAACATTCCGAAGTCACTGGAACGGCAGTGATGTCATCCTGTGTCAAAGTAGAGCA 600
NA              GGACAAACATTCCGAAGTCACTGGAACGGCAGTGATGTCATCCTGTGTCAAAGTAGAGCA 600
Zmp             GGACAAACATTCCGAAGTCACTGGAACGGCAGTGATGTCATCCTGTGTCAAAGTAGAGCA 600
                ************************************************************

NUMT_1          GTAGCGATAGTGAATTACTATTCTCATTAACAATAGGGCCTTTCTTCCTCCAATTCGTTA 660
NUMT_2          GTAGCGATAGTGAATTACTATTCTCATTAACAATAGGGCCTTTCTTCCTCCAATTCGTTA 660
NA              GTAGCGATAGTGAATTACTATTCTCATTAACAATAGGGCCTTTCTTCCTCCAATTCGTTA 660
Zmp             GTAGCGATAGTGAATTACTATTCTCATTAACAATAGGGCCTTTCTTCCTCCAATTCGTTA 660
                ************************************************************

NUMT_1          CGTTTTCTGATTGCACTACTTCCTTCTAATCCAACAATTATAAACCTAATCCAGAATATA 720
NUMT_2          CGTTTTCTGATTGCACTACTTCCTTCTAATCCAACAATTATAAACCTAATCCAGAATATA 720
NA              CGTTTTCTGATTGCACTACTTCCTTCTAATCCAACAATTATAAACCTAATCCAGAATATA 720
Zmp             CGTTTTCTGATTGCACTACTTCCTTCTAATCCAACAATTATAAACCTAATCCAGAATATA 720
                ************************************************************
```

```
NUMT_1          AGAATCCATTAATGAATACGATCTTCCCGTTTATCTCCATCTGCAGGGCTAGGATTGATT 780
NUMT_2          AGAATCCATTAATGAATACGATCTTCCCGTTTATCTCCATCTGCAGGGCTAGGATTGATT 780
NA              AGAATCCATTAATGAATACGATCTTCCCGTTTATCTCCATCTGCAGGGCTAGGATTGATT 780
Zmp             AGAATCCATTAATGAATACGATCTTCCCGTTTATCTCCATCTGCAGGGCTAGGATTGATT 780
                ************************************************************

NUMT_1          TCTTCTTCTCGATAGTGATGAGGCTAGTGCTCCTTCTCTTCTTCCCAATTCCTGGAAGCC 840
NUMT_2          TCTTCTTCTCGATAGTGATGAGGCTAGTGCTCCTTCTCTTCTTCCCAATTCCTGGAAGCC 840
NA              TCTTCTTCTCGATAGTGATGAGGCTAGTGCTCCTTCTCTTCTTCCCAATTCCTGGAAGCC 840
Zmp             TCTTCTTCTCGATAGTGATGAGGCTAGTGCTCCTTCTCTTCTTCCCAATTCCTGGAAGCC 840
                ************************************************************

NUMT_1          TTCTACTCCTCGAAACCCTGTCTTTTGTTTGATTTAGTTACAGATGGACTATCAAAGATA 900
NUMT_2          TTCTACTCCTCGAAACCCTGTCTTTTGTTTGATTTAGTTACAGATGGACTATCAAAGATA 900
NA              TTCTACTCCTCGAAACCCTGTCTTTTGTTTGATTTAGTTACAGATGGACTATCAAAGATA 900
Zmp             TTCTACTCCTCGAAACCCTGTCTTTTGTTTGATTTAGTTACAGATGGACTATCAAAGATA 900
                ************************************************************

NUMT_1          TTGGCTAGAGGTCAAGATGTTGGGTGCCATGGATCGAGTGATAAAATAAAATCAGAATTG 960
NUMT_2          TTGGCTAGAGGTCAAGATGTTGGGTGCCATGGATCGAGTGATAAAATAAAATCAGAATTG 960
NA              TTGGCTAGAGGTCAAGATG TGGGTGCCATGGATCGAGTGATAAAATAAAATCAGAATTG 960
Zmp             TTGGCTAGAGGTCAAGATG TGGGTGCCATGGATCGAGTGATAAAATAAAATCAGAATTG 960
                ******************* ****************************************

NUMT_1          TTTTCAAAGTGAAATGTTGCTCCTCAGAAAACGCGTATAGTAATCTCATTGGCCTTCGTC 1020
NUMT_2          TTTTCAAAGTGAAATGTTGCTCCTCAGAAAACGCGTATAGTAATCTCATTGGCCTTCGTC 1020
NA              TTTTCAAAGTGAAATGTTGCTCCTCAGAAAACGCGTATAGTAATCTCATTGGCCTTCGTC 1020
Zmp             TTTTCAAAGTGAAATGTTGCTCCTCAGAAAACGCGTATAGTAATCTCATTGGCCTTCGTC 1020
                ************************************************************

NUMT_1          GATGGGACAAATGCTCCAGATGAGTCCTTTTGCTCAAAAGAGAGAAGGGACAGGAATCTA 1080
NUMT_2          GATGGGACAAATGCTCCAGATGAGTCCTTTTGCTCAAAAGAGAGAAGGGACAGGAATCTA 1080
NA              GATGGGACAAATGCTCCAGATGAGTCCTTTTGCTCAAAAGAGAGAAGGGACAGGAATCTA 1080
Zmp             GATGGGACAAATGCTCCAGATGAGTCCTTTTGCTCAAAAGAGAGAAGGGACAGGAATCTA 1080
                ************************************************************

NUMT_1          TTCTATAAGTTGTAAAAAGATGTGCGTATACTGGTCGACGTCATGTGATCGCTACTAAAG 1140
NUMT_2          TTCTATAAGTTGTAAAAAGATGTGCGTATACTGGTCGACGTCATGTGATCGCTACTAAAG 1140
NA              TTCTATAAGTTGTAAAAAGATGTGCGTATACTGGTCGACGTCATGTGATCGCTACTAAAG 1140
Zmp             TTCTATAAGTTGTAAAAAGATGTGCGTATACTGGTCGACGTCATGTGATCGCTACTAAAG 1140
                ************************************************************

NUMT_1          ATAGAATTTCCTTTCCTTGGAAAAACCAAGGCCAGTTGAGAGAAGTCTTTCTGCTTAGAG 1200
NUMT_2          ATAGAATTTCCTTTCCTTGGAAAAACCAAGGCCAGTTGAGAGAAGTCTTTCTGCTTAGAG 1200
NA              ATAGAATTTCCTTTCCTTGGAAAAACCAAGGCCAGTTGAGAGAAGTCTTTCTGCTTAGAG 1200
Zmp             ATAGAATTTCCTTTCCTTGGAAAAACCAAGGCCAGTTGAGAGAAGTCTTTCTGCTTAGAG 1200
                ************************************************************

NUMT_1          CAAGAAGCGGAACCCAAATCAAGCTTTCTTTATTTTCATTTATGGATAACCAATTCATTG 1260
NUMT_2          CAAGAAGCGGAACCCAAATCAAGCTTTCTTTATTTTCATTTATGGATAACCAATTCATTG 1260
NA              CAAGAAGCGGAACCCAAATCAAGCTTTCTTTATTTTCATTTATGGATAACCAATTCATTG 1260
Zmp             CAAGAAGCGGAACCCAAATCAAGCTTTCTTTATTTTCATTTATGGATAACCAATTCATTG 1260
                ************************************************************

NUMT_1          ATTATGTAGGCATCGGAATCATTGTTTCCGTTGCCATTTTTTTTGCTTATAAAGCGGGGC 1320
NUMT_2          ATTATGTAGGCATCGGAATCATTGTTTCCGTTGCCATTTTTTTTGCTTATAAAGCGGGGC 1320
NA              ATTATGTAGGCATCGGAATCATTGTTTCCGTTGCCATTTTTTT GCTTATAAAGCGGGGC 1320
Zmp             ATTATGTAGGCATCGGAATCATTGTTTCCGTTGCCATTTTTTT GCTTATAAAGCGGGGC 1320
                ******************************************* ****************

NUMT_1          AGCTGGCTGAACGAATCCATTCCCACACATTGGAAATGGAAATACTCAAACAAAAGCTGG 1380
NUMT_2          AGCTGGCTGAACGAATCCATTCCCACACATTGGAAATGGAAATACTCAAACAAAAGCTGG 1380
NA              AGCTGGCTGAACGAATCCATTCCCACACATTGGAAATGGAAATACTCAAACAAAAGCTGG 1380
Zmp             AGCTGGCTGAACGAATCCATTCCCACACATTGGAAATGGAAATACTCAAACAAAAGCTGG 1380
                ************************************************************

NUMT_1          AATATAAGCTAAAAATGCTTTTGGAGCAAACTTCTGGTAATGCGCAATTGCCGGAGGGAT 1440
NUMT_2          AATATAAGCTAAAAATGCTTTTGGAGCAAACTTCTGGTAATGCGCAATTGCCGGAGGGAT 1440
NA              AATATAAGCTAAAAATGCTTTTGGAGCAAACTTCTGGTAATGCGCAATTGCCGGAGGGAT 1440
Zmp             AATATAAGCTAAAAATGCTTTTGGAGCAAACTTCTGGTAATGCGCAATTGCCGGAGGGAT 1440
                ************************************************************
```

```
NUMT_1          TCTCACTCCGGGATATCATTACAAATATGGTTTTCTCGGGGGACTCTATAGAAGAACAGC 1500
NUMT_2          TCTCACTCCGGGATATCATTACAAATATGGTTTTCTCGGGGGACTCTATAGAAGAACAGC 1500
NA              TCTCACTCCGGGATATCATTACAAATATGGTTTTCTCGGGGGACTCTATAGAAGAACAGC 1500
Zmp             TCTCACTCCGGGATATCATTACAAATATGGTTTTCTCGGGGGACTCTATAGAAGAACAGC 1500
                ************************************************************

NUMT_1          TTCTAGCATTAAATCGGATCTATCTTGATCTGGTCGTTCATGGACACAGTAGTGACTACT 1560
NUMT_2          TTCTAGCATTAAATCGGATCTATCTTGATCTGGTCGTTCATGGACACAGTAGTGACTACT 1560
NA              TTCTAGCATTAAATCGGATCTATCTTGATCTGGTCGTTCATGGACACAGTAGTGACTACT 1560
Zmp             TTCTAGCATTAAATCGGATCTATCTTGATCTGGTCGTTCATGGACACAGTAGTGACTACT 1560
                ************************************************************

NUMT_1          TTTTAATGATTCTCAATTAATTATTTTAATTAGCAGTTGCTGTAGGCAACTAGCATTTTG 1620
NUMT_2          TTTTAATGATTCTCAATTAATTATTTTAATTAGCAGTTGCTGTAGGCAACTAGCATTTTG 1620
NA              TTTTAATGATTCTCAATTAATTATTTTAATTAGCAGTTGCTGTAGGCAACTAGCATTTTG 1620
Zmp             TTTTAATGATTCTCAATTAATTATTTTAATTAGCAGTTGCTGTAGGCAACTAGCATTTTG 1620
                ************************************************************

NUMT_1          TTTTGTCATGGAATCAAGTCTATTTGTTCTTTTTTGCGTTTCGTTGGAAAAACCCACGCC 1680
NUMT_2          TTTTGTCATGGAATCAAGTCTATTTGTTCTTTTTTGCGTTTCGTTGGAAAAACCCACGCC 1680
NA              TTTTGTCATGGAATCAAGTCTATTTGTTCTTTTTTGCGTTTCGTTGGAAAAACCCACGCC 1680
Zmp             TTTTGTCATGGAATCAAGTCTATTTGTTCTTTTTT CGTTTCGTTGGAAAAACCCACGCC 1680
                *********************************** ************************

NUMT_1          AACCAAAATCCAAGTCTCCCTTTCTCTTTTGGGAGCAGATTGTATTGTATTTTATAAAGT 1740
NUMT_2          AACCAAAATCCAAGTCTCCCTTTCTCTTTTGGGAGCAGATTGTATTGTATTTTATAAAGT 1740
NA              AACCAAAATCCAAGTCTCCCTTTCTCTTTTGGGAGCAGATTGTATTGTATTTTATAAAGT 1740
Zmp             AACCAAAATCCAAGTCTCCCTTTCTCTTTTGGGAGCAGATTGTATTGTATTTTATAAAGT 1740
                ************************************************************

NUMT_1          TGAGAGTCACAATGAGATTTAGTGGAATGGATATGAAGGGTATAAATATGGTATTTGCTG 1800
NUMT_2          TGAGAGTCACAATGAGATTTAGTGGAATGGATATGAAGGGTATAAATATGGTATTTGCTG 1800
NA              TGAGAGTCACAATGAGATTTAGTGGAATGGATATGAAGGGTATAAATATGGTATTTGCTG 1800
Zmp             TGAGAGTCACAATGAGATTTAGTGGAATGGATATGAAGGGTATAAATATGGTATTTGCTG 1800
                ************************************************************

NUMT_1          CTATTCCGAAAGCTATGAAACAAGTCTGGGGAAACAGAAATTGGCACAATATCGCGAAGT 1860
NUMT_2          CTATTCCGAAAGCTATGAAACAAGTCTGGGGAAACAGAAATTGGCACAATATCGCGAAGT 1860
NA              CTATTCCGAAAGCTATGAAACAAGTCTGGGGAAACAGAAATTGGCACAATATCGCGAAGT 1860
Zmp             CTATTCCGAAAGCTATGAAACAAGTCTGGGGAAACAGAAATTGGCACAATATCGCGAAGT 1860
                ************************************************************

NUMT_1          GGCTGCCTTCGCTCAATTGTGCCTAACTTGGGCCATAAAGATTTTCGCTAATAACGGGAG 1920
NUMT_2          GGCTGCCTTCGCTCAATTGTGCCTAACTTGGGCCATAAAGATTTTCGCTAATAACGGGAG 1920
NA              GGCTGCCTTCGCTCAATTGTGCCTAACTTGGGCCATAAAGATTTTCGCTAATAACGGGAG 1920
Zmp             GGCTGCCTTCGCTCAATTGTGCCTAACTTGGGCCATAAAGATTTTCGCTAATAACGGGAG 1920
                ************************************************************

NUMT_1          TGGGAGTGCTCTCCTTCTTTCCTTCTGTTTTTTTTCTTTCTTCTTCTTTGCAACGACAAG 1980
NUMT_2          TGGGAGTGCTCTCCTTCTTTCCTTCTGTTTTTTTTCTTTCTTCTTCTTTGCAACGACAAG 1980
NA              TGGGAGTGCTCTCCTTCTTTCCTTCTGTTTTTTTTCTTTCTTCTTCTTTGCAACGACAAG 1980
Zmp             TGGGAGTGCTCTCCTTCTTTCCTTCTGTTTTTTTTCTTTCTTCTTCTTTGCAACGACAAG 1980
                ************************************************************

NUMT_1          GGCGGAGGCTGCAGGCCCATCGGATGGCCCATCGGATTGGATGAAAGGGCACCCGGATGA 2040
NUMT_2          GGCGGAGGCTGCAGGCCCATCGGATGGCCCATCGGATTGGATGAAAGGGCACCCGGATGA 2040
NA              GGCGGAGGCTGCAGGCCCATCGGATGGCCCATCGGATTGGATGAAAGGGCACCCGGATGA 2040
Zmp             GGCGGAGGCTGCAGGCCCATCGGATGGCCCATCGGATTGGATGAAAGGGCACCCGGATGA 2040
                ************************************************************

NUMT_1          AACCTTGCTTCGTAAAACGGAGAAGGAGATTCTACGGGTGCAAGAGGAGGTTGATACCCT 2100
NUMT_2          AACCTTGCTTCGTAAAACGGAGAAGGAGATTCTACGGGTGCAAGAGGAGGTTGATACCCT 2100
NA              AACCTTGCTTCGTAAAACGGAGAAGGAGATTCTACGGGTGCAAGAGGAGGTTGATACCCT 2100
Zmp             AACCTTGCTTCGTAAAACGGAGAAGGAGATTCTACGGGTGCAAGAGGAGGTTGATACCCT 2100
                ************************************************************

NUMT_1          CGCAACCAAAGCAGTAGAAAAAGGTCACTTTTATGGGCTAGGTCTGCCCGGTACCCCGGC 2160
NUMT_2          CGCAACCAAAGCAGTAGAAAAAGGTCACTTTTATGGGCTAGGTCTGCCCGGTACCCCGGC 2160
NA              CGCAACCAAAGCAGTAGAAAAAGGTCACTTTTATGGGCTAGGTCTGCCCGGTACCCCGGC 2160
Zmp             CGCAACCAAAGCAGTAGAAAAAGGTCACTTTTATGGGCTAGGTCTGCCCGGTACCCCGGC 2160
                ************************************************************
```

```
NUMT_1          GGAGCAGAAGGATACTATAAAATCTATTATCGACCTTGATCTAGATAGTATAGATCTGGA 2220
NUMT_2          GGAGCAGAAGGATACTATAAAATCTATTATCGACCTTGATCTAGATAGTATAGATCTGGA 2220
NA              GGAGCAGAAGGATACTATAAAATCTATTATCGACCTTGATCTAGATAGTATAGATCTGGA 2220
Zmp             GGAGCAGAAGGATACTATAAAATCTATTATCGACCTTGATCTAGATAGTATAGATCTGGA 2220
                ************************************************************

NUMT_1          TAAGCGGTCGAAAAGACTAAAAAGTTGGCTCAACAGCGGGGTAGATAACCCGGATAGCTC 2280
NUMT_2          TAAGCGGTCGAAAAGACTAAAAAGTTGGCTCAACAGCGGGGTAGATAACCCGGATAGCTC 2280
NA              TAAGCGGTCGAAAAGACTAAAAAGTTGGCTCAACAGCGGGGTAGATAACCCGGATAGCTC 2280
Zmp             TAAGCGGTCGAAAAGACTAAAAAGTTGGCTCAACAGCGGGGTAGATAACCCGGATAGCTC 2280
                ************************************************************

NUMT_1          TTTTTGGTTGATGCTCGTACACGAGATCACAAAATGGTATCCATAGGTTTTATTTGCATG 2340
NUMT_2          TTTTTGGTTGATGCTCGTACACGAGATCACAAAATGGTATCCATAGGTTTTATTTGCATG 2340
NA              TTTTTGGTTGATGCTCGTACACGAGATCACAAAATGGTATCCATAGGTTTTATTTGCATG 2340
Zmp             TTTTTGGTTGATGCTCGTACACGAGATCACAAAATGGTATCCATAGGTTTTATTTGCATG 2340
                ************************************************************

NUMT_1          TTAGAAAGGAAAGGAAAGGAGATAGAGGTGCAAGGCTTACAGAAGTGCCCAAAGTTTCA 2399
NUMT_2          TTAGAAAGGAAAGGAAAGGAGATAGAGGTGCAAGGCTTACAGAAGTGCCCAAAGTTTCA 2399
NA              TTAGAAAGGAAAGGAAAGGAGATAGAGGTGCAAGGCTTACAGAAGTGCCCAAAGTTTCA 2399
Zmp             TTAGAAAGGAAAGGAAAGGAGATAGAGGTGCAAGGCTTACAGAAGTGCCCAAAGTTTCA 2399
                ***********************************************************
```

Figure S5 Multiple sequence alignment of the two B73 2.4-kb NUMT regions with the corresponding NA and Zmp mitochondrial genome regions. The 2.4-kb NUMT region is present within both the NA (NCBI Accession DQ490952.1) and Zmp (NCBI Accession DQ645539.1) mitochondrial genomes, but has fewer nucleotide differences when compared to the NA genome. The sequences were aligned with ClustalW2 (McWilliam *et al.* 2013). The reverse complement of the second 2.4-kb region in the NUMT was used in this alignment.
