# Supplementary material for: Cytogenetic and Sequence Analyses of Mitochondrial DNA Insertions in Nuclear Chromosomes of Maize
Source: G3 (Bethesda). 2015 Sep 1;5(11):2229–39. doi: 10.1534/g3.115.020677 (PMC4632043; doi:10.1534/g3.115.020677)
Supplement: Supporting Information [file supp_g3.115.020677_FigureS6.pdf]

```
NUMT_1          TATTACAATAGACGTTGAAACAACCCTGGAGCTTATCTGTAATTTGCTCCTTGAGCGTTT 60
NUMT_3          TATTACAATAGACGTTGAAACAACCCTGGAGCTTATCTGTAATTTGCTCCTTGAGCGTTT 60
NA              TATTACAATAGACGTTGAAACAACCCTGGAGCTTATCTGTAATTTGCTCCTTGAGCGTTT 60
Zmp             TATTACAATAGACGTTGAAACAACCCTGGAGCTTATCTGTAATTTGCTCCTTGAGCGTTT 60
CMS-S           TATTACAATAGACGTTGAAACAACCCTGGAGCTTATCTGTAATTTGCTCCTTGAGCGTTT 60
NUMT_2          TATTACAATAGACGTTGAAACAACCCTGGAGCTTATCTGTAATTTGCTCCTTGAGCGTTT 60
CMS-T           TATTACAATAGACGTTGAAACAACCCTGGAGCTTATCTGTAATTTGCTCCTTGAGCGTTT 60
                ************************************************************

NUMT_1          CTAACGTCAATAAA-----GTCCTCCAACTTATGATGCCAGTTTTCCGAAGCCGCGGCTT 115
NUMT_3          CTAACGTCAATAAA-----GTCCTCCAACTTATGATGCCAGTTTTCCGAAGCCGCGGCTT 115
NA              CTAACGTCAATAAA-----GTCCTCCAACTTATGATGCCAGTTTTCCGAAGCCGCGGCTT 115
Zmp             CTAACGTCAATAAA-----GTCCTCCAACTTATGATGCCAGTTTTCCGAAGCCGCGGCTT 115
CMS-S           CTAACGTCAATAAA-----GTCCTCCAACTTATGATGCCAGTTTTCCGAAGCCGCGGCTT 115
NUMT_2          CTAACGTCAATAAA-----GTCCTCCAACTTATGATGCCAGTTTTCCGAAGCCGCGGCTT 115
CMS-T           CTAACGTCAATAAA     GTCCTCCAACTTATGATGCCAGTTTTCCGAAGCCGCGGCTT 120
                **************     *****************************************

NUMT_1          TTACCCGCTTTATAAGCGATGAGTAGGGCGATGCATAAAAAGTCATATTTCTTGGTGTAG 175
NUMT_3          TTACCCGCTTTATAAGCGATGAGTAGGGCGATGCATAAAAAGTCATATTTCTTGGTGTAG 175
NA              TTACCCGCTTTATAAGCGATGAGTAGGGCGATGCATAAAAAGTCATATTTCTTGGTGTAG 175
Zmp             TTACCCGCTTTATAAGCGATGAGTAGGGCGATGCATAAAAAGTCATATTTCTTGGTGTAG 175
CMS-S           TTACCCGCTTTATAAGCGATGAGTAGGGCGATGCATAAAAAGTCATATTTCTTGGTGTAG 175
NUMT_2          TTACCCGCTTTATAAGCGATGAGTAGGGCGATGCATAAAAAGTCATATTTCTTGGTGTAG 175
CMS-T           TTACCCGCTTTATAAGCGATGAGTAGGGCGATGCATAAAAAGTCATATTTCTTGGTGTAG 180
                ************************************************************

NUMT_1          GGATGGATCTCATAGGAAAAGAGATACCGAGGCCCACCAACCGTATACTTGATTTATGGT 235
NUMT_3          GGATGGATCTCATAGGAAAAGAGATACCGAGGCCCACCAACCGTATACTTGATTTATGGT 235
NA              GGATGGATCTCATAGGAAAAGAGATACCGAGGCCCACCAACCGTATACTTGATTTATGGT 235
Zmp             GGATGGATCTCATAGGAAAAGAGATACCGAGGCCCACCAACCGTATACTTGATTTATGGT 235
CMS-S           GGATGGATCTCATAGGAAAAGAGATACCGAGGCCCACCAACCGTATACTTGATTTATGGT 235
NUMT_2          GGATGGATCTCATAGGAAAAGAGATACCGAGGCCCACCAACC TATACTTGATTTATGGT 235
CMS-T           GGA    TCTCATAGGAAAAGAGATACCGAGGCCCACCAACCGTATACTTGATTTATGGT 236
                ***    *********************************** *****************

NUMT_1          TTGGTGGGGAAAGAAGAGTGGGTATGGGGCTTCTTTCATGGTGCCATTCTTTACTTTACG 295
NUMT_3          TTGGTGGGGAAAGAAGAGTGGGTATGGGGCTTCTTTCATGGTGCCATTCTTTACTTTACG 295
NA              TTGGTGGGGAAAGAAGAGTGGGTATGGGGCTTCTTTCATGGTGCCATTCTTTACTTTACG 295
Zmp             TTGGTGGGGAAAGAAGAGTGGGTATGGGGCTTCTTTCATGGTGCCATTCTTTACTTTACG 295
CMS-S           TTGGTGGGGAAAGAAGAGTGGGTATGGGGCTTCTTTCATGGTGCCATTCTTTACTTTACG 295
NUMT_2          TTGGTGGGGAAAGAAGAGTGGGTATGGGGCTTCTTTCATGGTGCCATTCTTTACTTTACG 295
CMS-T           TTGGTGGGGAAAGAAGAGTGGGTATGGGGCTTCTTTCATGGTGCCATTCTTTACTTTACG 296
                ************************************************************

NUMT_1          TAATAAAAATCAGAGAGGGACTGAACACTTGTTTTGATCTACGAAGAGTTGAAAAACAAT 355
NUMT_3          TAATAAAAATCAGAGAGGGACTGAACACTTGTTTTGATCTACGAAGAGTTGAAAAACAAT 355
NA              TAATAAAAATCAGAGAGGGACTGAACACTTGTTTTGATCTACGAAGAGTTGAAAAACAAT 355
Zmp             TAATAAAAATCAGAGAGGGACTGAACACTTGTTTTGATCTACGAAGAGTTGAAAAACAAT 355
CMS-S           TAATAAAAATCAGAGAGGGACTGAACACTTGTTTTGATCTACGAAGAGTTGAAAAACAAT 355
NUMT_2          TAATAAAAATCAGAGAGGGACTGAACACTTGTTTTGATCTACGAAGAGTTGAAAAACAAT 355
CMS-T           TAATAAAAATCAGAGAGGGACTGAACACTTGTTTTGATCTACGAAGAGTTGAAAAACAAT 356
                ************************************************************

NUMT_1          TGAATTGCCTTTATTTGATCTTAAAGAATCGGCATTGGCTTCAGTTCAGATCTTATGGGA 415
NUMT_3          TGAATTGCCTTTATTTGATCTTAAAGAATCGGCATTGGCTTCAGTTCAGATCTTATGGGA 415
NA              TGAATTGCCTTTATTTGATCTTAAAGAATCGGCATTGGCTTCAGTTCAGATCTTATGGGA 415
Zmp             TGAATTGCCTTTATTTGATCTTAAAGAATCGGCATTGGCTTCAGTTCAGATCTTATGGGA 415
CMS-S           TGAATTGCCTTTATTTGATCTTAAAGAATCGGCATTGGCTTCAGTTCAGATCTTATGGGA 415
NUMT_2          TGAATTGCCTTTATTTGATCTTAAAGAATCGGCATTGGCTTCAGTTCAGATCTTATGGGA 415
CMS-T           TGAATTGCCTTTATTTGATCTTAAAGAATCGGCATTGGCTTCAGTTCAGATCTTATGGGA 416
                ************************************************************

NUMT_1          AAAGGCGCGTAGCGAAGAAGTGTATGCTCAATAAACTGAAGAAGCATATATAAGAAAGAA 475
NUMT_3          AAAGGCGCGTAGCGAAGAAGTGTATGCTCAATAAACTGAAGAAGCATATATAAGAAAGAA 475
NA              AAAGGCGCGTAGCGAAGAAGTGTATGCTCAATAAACTGAAGAAGCATATATAAGAAAGAA 475
Zmp             AAAGGCGCGTAGCGAAGAAGTGTATGCTCAATAAACTGAAGAAGCATATATAAGAAAGAA 475
CMS-S           AAAGGCGCGTAGCGAAGAAGTGTATGCTCAATAAACTGAAGAAGCATATATAAGAAAGAA 475
NUMT_2          AAAGGCGCGTAGCGAAGAAGTGTATGCTCAATAAACTGAAGAAGCATATATAAGAAAGAA 475
CMS-T           AAAGGCGCGTAGCGAAGAAGTGTATGCTCAATAAACTGAAGAAGCATATATAAGAAAGAA 476
                ************************************************************
```

```
NUMT_1          GCCATCATCACTATGTTTACTCCTACCCAAGGAAGGAGGTCAGTCAAAGCAATGAAATGA 535
NUMT_3          GCCATCATCACTATGTTTACTCCTACCCAAGGAAGGAGGTCAGTCAAAGCAATGAAATGA 535
NA              GCCATCATCACTATGTTTACTCCTACCCAAGGAAGGAGGTCAGTCAAAGCAATGAAATGA 535
Zmp             GCCATCATCACTATGTTTACTCCTACCCAAGGAAGGAGGTCAGTCAAAGCAATGAAATGA 535
CMS-S           GCCATCATCACTATGTTTACTCCTACCCAAGGAAGGAGGTCAGTCAAAGCAATGAAATGA 535
NUMT_2          GCCATCATCACTATGTTTACTCCTACCCAAGGAAGGAGGTCAGTCAAAGCAATGAAATGA 535
CMS-T           GCCATCATCACTATGTTTACTCCTACCCAAGGAAGGAGGTCAGTCAAAGCAATGAAATGA 536
                ************************************************************

NUMT_1          AGAGGCCGTGACCTAATACCTAT TGTGTTCTCTTGGATAGTGGTCCAGTTTGAATAGTT 595
NUMT_3          AGAGGCCGTGACCTAATACCTAT TGTGTTCTCTTGGATAGTGGTCCAGTTTGAATAGTT 595
NA              AGAGGCCGTGACCTAATACCTATCTGTGTTCTCTTGGATAGTGGTCCAGTTTGAATAGTT 595
Zmp             AGAGGCCGTGACCTAATACCTATCTGTGTTCTCTTGGATAGTGGTCCAGTTTGAATAGTT 595
CMS-S           AGAGGCCGTGACCTAATACCTATCTGTGTTCTCTTGGATAGTGGTCCAGTTTGAATAGTT 595
NUMT_2          AGAGGC GTGACCTAATACCTATCTGTGTTCTCTTGGATAGTGGTCCAGTTTGAATAGTT 595
CMS-T           AGAGGCCGTGACCTAATACCTATCTGTGTTCTCTTGGATAGTGGTCCAGTTTGAATAGTT 596
                ****** **************** ************************************

NUMT_1          GTATAGTTATTTGTAAACCCGGGGGCCCTGAAATTAGCCAAAACAACCCGGTGGGGTAAA 655
NUMT_3          GTATAGTTATTTGTAAACCCGGGGGCCCTGAAATTAGCCAAAACAACCCGGTGGGGTAAA 655
NA              GTATAGTTATTTGTAAACCCGGGGGCCCTGAAATTAGCCAAAACAACCCGGTGGGGTAAA 655
Zmp             GTATAGTTATTTGTAAACCCGGGGGCCCTGAAATTAGCCAAAACAACCCGGTGGGGTAAA 655
CMS-S           GTATAGTTATTTGTAAACCCGGGGGCCCTGAAATTAGCCAAAACAACCCGGTGGGGTAAA 655
NUMT_2          GTATAGTTATTTGTAAACCCGGGGGCCCTGAAATTAGCCAAAACAACCCGGTGGGGTAAA 655
CMS-T           GTATAGTTATTTGTAAACCCGGGGGCCCTGAAATTAGCCAAAACAACCCGGTGGGGTAAA 656
                ************************************************************

NUMT_1          GTCGTCAAGTGGACTATGGTTCACAATAATAGTGACTGACACGAGATGCGATGCCAAGTT 715
NUMT_3          GTCGTCAAGTGGACTATGGTTCACAATAATAGTGACTGACACGAGATGCGATGCCAAGTT 715
NA              GTCGTCAAGTGGACTATGGTTCACAATAATAGTGACTGACACGAGATGCGATGCCAAGTT 715
Zmp             GTCGTCAAGTGGACTATGGTTCACAATAATAGTGACTGACACGAGATGCGATGCCAAGTT 715
CMS-S           GTCGTCAAGTGGACTATGGTTCACAATAATAGTGACTGACACGAGATGCGATGCCAAGTT 715
NUMT_2          GTCGTCAAGTGGACTATGGTTCACAATAATAGTGACTGACACGAGATGCGATGCCAAGTT 715
CMS-T           GTCGTCAAGTGGACTATGGTTCACAATAATAGTGACTGACACGAGATGCGATGCCAAGTT 716
                ************************************************************

NUMT_1          AGAAGGTCAAAAGTGAGAAAGTTGGAGGGGAGATGCCATGATCCTAGGTGTAGATTGGCT 775
NUMT_3          AGAAGGTCAAAAGTGAGAAAGTTGGAGGGGAGATGCCATGATCCTAGGTGTAGATTGGCT 775
NA              AGAAGGTCAAAAGTGAGAAAGTTGGAGGGGAGATGCCATGATCCTAGGTGTAGATTGGCT 775
Zmp             AGAAGGTCAAAAGTGAGAAAGTTGGAGGGGAGATGCCATGATCCTAGGTGTAGATTGGCT 775
CMS-S           AGAAGGTCAAAAGTGAGAAAGTTGGAGGGGAGATGCCATGATCCTAGGTGTAGATTGGCT 775
NUMT_2          AGAAGGTCAAAAGTGAGAAAGTTGGAGGGGAGATGCCATGATCCTAGGTGTAGATTGGCT 775
CMS-T           AGAAGGTCAAAAGTGAGAAAGTTGGAGGGGAGATGCCATGATCCTAGGTGTAGATTGGCT 776
                ************************************************************

NUMT_1          TATCAGCCTATGGCCATTCCTTTAATACTTTTGAACGATGACTTAATGCTTCAAAGCCCT 835
NUMT_3          TATCAGCCTATGGCCATTCCTTTAATACTTTTGAACGATGACTTAATGCTTCAAAGCCCT 835
NA              TATCAGCCTATGGCCATTCCTTTAATACTTTTGAACGATGACTTAATGCTTCAAAGCCCT 835
Zmp             TATCAGCCTATGGCCATTCCTTTAATACTTTTGAACGATGACTTAATGCTTCAAAGCCCT 835
CMS-S           TATCAGCCTATGGCCATTCCTTTAATACTTTTGAACGATGACTTAATGCTTCAAAGCCCT 835
NUMT_2          TATCAGCCTATGGCCATTCCTTTAATACTTTTGAACGATGACTTAATGCTTCAAAGCCCT 835
CMS-T           TATCAGCCTATGGCCATTCCTTTAATACTTTTGAACGATGACTTAATGCTTCAAAGCCCT 836
                ************************************************************

NUMT_1          CCCCATATAGCACAGCCTTCGTAAGGCTTATCACACTCGGGCCTGTAACTGGAATCTGAG 895
NUMT_3          CCCCATATAGCACAGCCTTCGTAAGGCTTATCACACTCGGGCCTGTAACTGGAATCTGAG 895
NA              CCCCATATAGCACAGCCTTCGTAAGGCTTATCACACTCGGGCCTGTAACTGGAATCTGAG 895
Zmp             CCCCATATAGCACAGCCTTCGTAAGGCTTATCACACTCGGGCCTGTAACTGGAATCTGAG 895
CMS-S           CCCCATATAGCACAGCCTTCGTAAGGCTTATCACACTCGGGCCTGTAACTGGAATCTGAG 895
NUMT_2          CCCCATATAGCACAGCCTTCGTAAGGCTTATCACACTCGGGCCTGTAACTGGAATCTGAG 895
CMS-T           CCCCATATAGCACAGCCTTCGTAAGGCTTATCACACTCGGGCCTGTAACTGGAATCTGAG 896
                ************************************************************

NUMT_1          CATTTCTTGTTTGGGGTTCGCAAACCGACTTAAGTCTTTATTTTCTAATAGGCTTGGCCT 955
NUMT_3          CATTTCTTGTTTGGGGTTCGCAAACCGACTTAAGTCTTTATTTTCTAATAGGCTTGGCCT 955
NA              CATTTCTTGTTTGGGGTTCGCAAACCGACTTAAGTCTTTATTTTCTAATAGGCTTGGCCT 955
Zmp             CATTTCTTGTTTGGGGTTCGCAAACCGACTTAAGTCTTTATTTTCTAATAGGCTTGGCCT 955
CMS-S           CATTTCTTGTTTGGGGTTCGCAAACCGACTTAAGTCTTTATTTTCTAATAGGCTTGGCCT 955
NUMT_2          CATTTCTTGTTTGGGGTTCGCAAACCGACTTAAGTCTTTATTTTCTAATAGGCTTGGCCT 955
CMS-T           CATTTCTTGTTTGGGGTTCGCAAACCGACTTAAGTCTTTATTTTCTAATAGGCTTGGCCT 956
                ************************************************************
```

```
NUMT_1          TCGGGGGAACAAAGCCATTCCATGGAACCTGAGACTTTGATTCCAGCCTACTTCTACTTG 1015
NUMT_3          TCGGGGGAACAAAGCCATTCCATGGAACCTGAGACTTTGATTCCAGCCTACTTCTACTTG 1015
NA              TCGGGGGAACAAAGCCATTCCATGGAACCTGAGACTTTGATTCCAGCCTACTTCTACTTG 1015
Zmp             TCGGGGGAACAAAGCCATTCCATGGAACCTGAGACTTTGATTCCAGCCTACTTCTACTTG 1015
CMS-S           TCGGGGGAACAAAGCCATTCCATGGAACCTGAGACTTTGATTCCAGCCTACTTCTACTTG 1015
NUMT_2          TCGGGGGAACAAAGCCATTCCATGGAACCTGAGACTTTGATTCCAGCCTACTTCTACTTG 1015
CMS-T           TCGGGGGAACAAAGCCATTCCATGGAACCTGAGACTTTGATTCCAGCCTACTTCTACTTG 1016
                ************************************************************

NUMT_1          AAAAGCTTCTTCGTCGACAAATTCTTAGACCCCGTTGTCCAGTTTTGAACGATGGGAAGT 1075
NUMT_3          AAAAGCTTCTTCGTCGACAAATTCTTAGACCCCGTTGTCCAGTTTTGAACGATGGGAAGT 1075
NA              AAAAGCTTCTTCGTCGACAAATTCTTAGACCCCGTTGTCCAGTTTTGAACGATGGGAAGT 1075
Zmp             AAAAGCTTCTTCGTCGACAAATTCTTAGACCCCGTTGTCCAGTTTTGAACGATGGGAAGT 1075
CMS-S           AAAAGCTTCTTCGTCGACAAATTCTTAGACCCCGTTGTCCAGTTTTGAACGATGGGAAGT 1075
NUMT_2          AAAAGCTTCTTCGTCGACAAATTCTTAGACCCCGTTGTCCAGTTTTGAACGATGGGAAGT 1075
CMS-T           AAAAGCTTCTTCGTCGACAAATTCTTAGACCCCGTTGTCCAGTTTTGAACGATGGGAAGT 1076
                ************************************************************

NUMT_1          AGAAATATCTTTCCCACATTAGCGATTTGAGCAACCTCCGTTGAAAGCTTTCTTTGCCCC 1135
NUMT_3          AGAAATATCTTTCCCACATTAGCGATTTGAGCAACCTCCGTTGAAAGCTTTCTTTGCCCC 1135
NA              AGAAATATCTTTCCCACATTAGCGATTTGAGCAACCTCCGTTGAAAGCTTTCTTTGCCCC 1135
Zmp             AGAAATATCTTTCCCACATTAGCGATTTGAGCAACCTCCGTTGAAAGCTTTCTTTGCCCC 1135
CMS-S           AGAAATATCTTTCCCACATTAGCGATTTGAGCAACCTCCGTTGAAAGCTTTCTTTGCCCC 1135
NUMT_2          AGAAATATCTTTCCCACATTAGCGATTTGAGCAACCTCCGTTGAAAGCTTTCTTTGCCCC 1135
CMS-T           AGAAATATCTTTCCCACATTAGCGATTTGAGCAACCTCCGTTGAAAGCTTTCTTTGCCCC 1136
                ************************************************************

NUMT_1          GCCAAAGGAAAGGGCATTTGTCATCCTTGGTCTTCGTTCAGGCAAGTCTAATCACACAAG 1195
NUMT_3          GCCAAAGGAAAGGGCATTTGTCATCCTTGGTCTTCGTTCAGGCAAGTCTAATCACACAAG 1195
NA              GCCAAAGGAAAGGGCATTTGTCATCCTTGGTCTTCGTTCAGGCAAGTCTAATCACACAAG 1195
Zmp             GCCAAAGGAAAGGGCATTTGTCATCCTTGGTCTTCGTTCAGGCAAGTCTAATCACACAAG 1195
CMS-S           GCCAAAGGAAAGGGCATTTGTCATCCTTGGTCTTCGTTCAGGCAAGTCTAATCACACAAG 1195
NUMT_2          GCCAAAGGAAAGGGCATTTGTCATCCTTGGTCTTCGTTCAGGCAAGTCTAATCACACAAG 1195
CMS-T           GCCAAAGGAAAGGGCATTTGTCATCCTTGGTCTTCGTTCAGGCAAGTCTAATCACACAAG 1196
                ************************************************************

NUMT_1          TGAGATAGAAACACTGATTCGGGAGTATCAACTAATTTCATTTAAATGGCTGGGAAAGAA 1255
NUMT_3          TGAGATAGAAACACTGATTCGGGAGTATCAACTAATTTCATTTAAATGGCTGGGAAAGAA 1255
NA              TGAGATAGAAACACTGATTCGGGAGTATCAACTAATTTCATTTAAATGGCTGGGAAAGAA 1255
Zmp             TGAGATAGAAACACTGATTCGGGAGTATCAACTAATTTCATTTAAATGGCTGGGAAAGAA 1255
CMS-S           TGAGATAGAAACACTGATTCGGGAGTATCAACTAATTTCATTTAAATGGCTGGGAAAGAA 1255
NUMT_2          TGAGATAGAAACACTGATTCGGGAGTATCAACTAATTTCATTTAAATGGCTGGGAAAGAA 1255
CMS-T           TGAGATAGAAACACTGATTCGGGAGTATCAACTAATTTCATTTAAATGGCTGGGAAAGAA 1256
                ************************************************************

NUMT_1          ATCAACTAATTGGTTGGTAGCCCGCTTGGTACTAAGAGGTTCCTTGCCCTTCTTCCATTT 1315
NUMT_3          ATCAACTAATTGGTTGGTAGCCCGCTTGGTACTAAGAGGTTCCTTGCCCTTCTTCCATTT 1315
NA              ATCAACTAATTGGTTGGTAGCCCGCTTGGTACTAAGAGGTTCCTTGCCCTTCTTCCATTT 1315
Zmp             ATCAACTAATTGGTTGGTAGCCCGCTTGGTACTAAGAGGTTCCTTGCCCTTCTTCCATTT 1315
CMS-S           ATCAACTAATTGGTTGGTAGCCCGCTTGGTACTAAGAGGTTCCTTGCCCTTCTTCCATTT 1315
NUMT_2          ATCAACTAATTGGTTGGTAGCCCGCTTGGTACTAAGAGGTTCCTTGCCCTTCTTCCATTT 1315
CMS-T           ATCAACTAATTGGTTGGTAGCCCGCTTGGTACTAAGAGGTTCCTTGCCCTTCTTCCATTT 1316
                ************************************************************

NUMT_1          GGTAAAGTGATATTTTTAGGATTTGCTGGCAGGGAAGTATGTTTGTAGGTCAAAGTTGGA 1375
NUMT_3          GGTAAAGTGATATTTTTAGGATTTGCTGGCAGGGAAGTATGTTTGTAGGTCAAAGTTGGA 1375
NA              GGTAAAGTGATATTTTTAGGATTTGCTGGCAGGGAAGTATGTTTGTAGGTCAAAGTTGGA 1375
Zmp             GGTAAAGTGATATTTTTAGGATTTGCTGGCAGGGAAGTATGTTTGTAGGTCAAAGTTGGA 1375
CMS-S           GGTAAAGTGATATTTTTAGGATTTGCTGGCAGGGAAGTATGTTTGTAGGTCAAAGTTGGA 1375
NUMT_2          GGTAAAGTGATATTTTTAGGATTTGCTGGCAGGGAAGTATGTTTGTAGGTCAAAGTTGGA 1375
CMS-T           GGTAAAGTGATATTTTTAGGATTTGCTGGCAGGGAAGTATGTTTGTAGGTCAAAGTTGGA 1376
                ************************************************************

NUMT_1          ATTAGAATCGGGGAACTCGGTAAAGTAGCTTTCATAGCTTTCACATATTTGTGAGCCCTA 1435
NUMT_3          ATTAGAATCGGGGAACTCGGTAAAGTAGCTTTCATAGCTTTCACATATTTGTGAGCCCTA 1435
NA              ATTAGAATCGGGGAACTCGGTAAAGTAGCTTTCATAGCTTTCACATATTTGTGAGCCCTA 1435
Zmp             ATTAGAATCGGGGAACTCGGTAAAGTAGCTTTCATAGCTTTCACATATTTGTGAGCCCTA 1435
CMS-S           ATTAGAATCGGGGAACTCGGTAAAGTAGCTTTCATAGCTTTCACATATTTGTGAGCCCTA 1435
NUMT_2          ATTAGAATCGGGGAACTCGGTAAAGTAGCTTTCATAGCTTTCACATATTTGTGAGCCCTA 1435
CMS-T           ATTAGAATCGGGGAACTCGGTAAAGTAGCTTTCATAGCTTTCACATATTTGTGAGCCCTA 1436
                ************************************************************
```

```
NUMT_1          TGGGTAGATAGAAGAAGGTACCCGACATCATCCACCTTACGTTGAGACAGAAGAGAAAGA 1495
NUMT_3          TGGGTAGATAGAAGAAGGTACCCGACATCATCCACCTTACGTTGAGACAGAAGAGAAAGA 1495
NA              TGGGTAGATAGAAGAAGGTACCCGACATCATCCACCTTACGTTGAGACAGAAGAGAAAGA 1495
Zmp             TGGGTAGATAGAAGAAGGTACCCGACATCATCCACCTTACGTTGAGACAGAAGAGAAAGA 1495
CMS-S           TGGGTAGATAGAAGAAGGTACCCGACATCATCCACCTTACGTTGAGACAGAAGAGAAAGA 1495
NUMT_2          TGGGTAGATAGAAGAAGGTACCCGACATCATCCACCTTACGTTGAGACAGAAGAGAAAGA 1495
CMS-T           TGGGTAGATAGAAGAAGGTACCCGACATCATCCACCTTACGTTGAGACAGAAGAGAAAGA 1496
                ************************************************************

NUMT_1          AGTTTATTCCCTCGCTAGCAAGCTTCTTTTCTTCTACTACCTGATGATTGAATTGGACTT 1555
NUMT_3          AGTTTATTCCCTCGCTAGCAAGCTTCTTTTCTTCTACTACCTGATGATTGAATTGGACTT 1555
NA              AGTTTATTCCCTCGCTAGCAAGCTTCTTTTCTTCTACTACCTGATGATTGAATTGGACTT 1555
Zmp             AGTTTATTCCCTCGCTAGCAAGCTTCTTTTCTTCTACTACCTGATGATTGAATTGGACTT 1555
CMS-S           AGTTTATTCCCTCGCTAGCAAGCTTCTTTTCTTCTACTACCTGATGATTGAATTGGACTT 1555
NUMT_2          AGTTTATTCCCTCGCTAGCAAGCTTCTTTTCTTCTACTACCTGATGATTGAATTGGACTT 1555
CMS-T           AGTTTATTCCCTCGCTAGCAAGCTTCTTTTCTTCTACTACCTGATGATTGAATTGGACTT 1556
                ************************************************************

NUMT_1          CCTTCCCCACAGAACCCAAGATGGTTGACTACTAGGCTCACAACTCTACGCAAAGGTAGG 1615
NUMT_3          CCTTCCCCACAGAACCCAAGATGGTTGACTACTAGGCTCACAACTCTACGCAAAGGTAGG 1615
NA              CCTTCCCCACAGAACCCAAGATGGTTGACTACTAGGCTCACAACTCTACGCAAAGGTAGG 1615
Zmp             CCTTCCCCACAGAACCCAAGATGGTTGACTACTAGGCTCACAACTCTACGCAAAGGTAGG 1615
CMS-S           CCTTCCCCACAGAACCCAAGATGGTTGACTACTAGGCTCACAACTCTACGCAAAGGTAGG 1615
NUMT_2          CCTTCCCCACAGAACCCAAGATGGTTGACTACTAGGCTCACAACTCTACGCAAAGGTAGG 1615
CMS-T           CCTTCCCCACAGAACCCAAGATGGTTGACTACTAGGCTCACAACTCTACGCAAAGGTAGG 1616
                ************************************************************

NUMT_1          CTCGATGAAGAAAGCGCAGGGTTACTTTCTTGCCTGAGGAAGTAGTTAGAAGTACTTTCT 1675
NUMT_3          CTCGATGAAGAAAGCGCAGGGTTACTTTCTTGCCTGAGGAAGTAGTTAGAAGTACTTTCT 1675
NA              CTCGATGAAGAAAGCGCAGGGTTACTTTCTTGCCTGAGGAAGTAGTTAGAAGTACTTTCT 1675
Zmp             CTCGATGAAGAAAGCGCAGGGTTACTTTCTTGCCTGAGGAAGTAGTTAGAAGTACTTTCT 1675
CMS-S           CTCGATGAAGAAAGCGCAGGGTTACTTTCTTGCCTGAGGAAGTAGTTAGAAGTACTTTCT 1675
NUMT_2          CTCGATGAAGAAAGCGCAGGGTTACTTTCTTGCCTGAGGAAGTAGTTAGAAGTACTTTCT 1675
CMS-T           CTCGATGAAGAAAGCGCAGGGTTACTTTCTTGCCTGAGGAAGTAGTTAGAAGTACTTTCT 1676
                ************************************************************

NUMT_1          TATTAATCTGCTTGATGTACATAAATCGATGGTTAAGGCGCGCAGCGGTAAGGTTCCCAC 1735
NUMT_3          TATTAATCTGCTTGATGTACATAAATCGATGGTTAAGGCGCGCAGCGGTAAGGTTCCCAC 1735
NA              TATTAATCTGCTTGATGTACATAAATCGATGGTTAAGGCGCGCAGCGGTAAGGTTCCCAC 1735
Zmp             TATTAATCTGCTTGATGTACATAAATCGATGGTTAAGGCGCGCAGCGGTAAGGTTCCCAC 1735
CMS-S           TATTAATCTGCTTGATGTACATAAATCGATGGTTAAGGCGCGCAGCGGTAAGGTTCCCAC 1735
NUMT_2          TATTAATCTGCTTGATGTACATAAATCGATGGTTAAGGCGCGCAGCGGTAAGGTTCCCAC 1735
CMS-T           TATTAATCTGCTTGATGTACATAAATCGATGGTTAAGGCGCGCAGCGGTAAGGTTCCCAC 1736
                ************************************************************

NUMT_1          CGAATCAAGAATTCGGCAATTGAGGAGCTCGATTAGTCATACTCACCACCACCAGCTCTA 1795
NUMT_3          CGAATCAAGAATTCGGCAATTGAGGAGCTCGATTAGTCATACTCACCACCACCAGCTCTA 1795
NA              CGAATCAAGAATTCGGCAATTGAGGAGCTCGATTAGTCATACTCACCACCACCAGCTCTA 1795
Zmp             CGAATCAAGAATTCGGCAATTGAGGAGCTCGATTAGTCATACTCACCACCACCAGCTCTA 1795
CMS-S           CGAATCAAGAATTCGGCAATTGAGGAGCTCGATTAGTCATACTCACCACCACCAGCTCTA 1795
NUMT_2          CGAATCAAGAATTCGGCAATTGAGGAGCTCGATTAGTCATACTCACCACCACCAGCTCTA 1795
CMS-T           CGAATCAAGAATTCGGCAATTGAGGAGCTCGATTAGTCATACTCACCACCACCAGCTCTA 1796
                ************************************************************

NUMT_1          GGCCCATCTTTTATATCTATACCGGAAAAGCGCTTCGCTTGATAACGGCATTCATAAATG 1855
NUMT_3          GGCCCATCTTTTATATCTATACCGGAAAAGCGCTTCGCTTGATAACGGCATTCATAAATG 1855
NA              GGCCCATCTTTTATATCTATACCGGAAAAGCGCTTCGCTTGATAACGGCATTCATAAATG 1855
Zmp             GGCCCATCTTTTATATCTATACCGGAAAAGCGCTTCGCTTGATAACGGCATTCATAAATG 1855
CMS-S           GGCCCATCTTTTATATCTATACCGGAAAAGCGCTTCGCTTGATAACGGCATTCATAAATG 1855
NUMT_2          GGCCCATCTTTTATATCTATACCGGAAAAGCGCTTCGCTTGATAACGGCATTCATAAATG 1855
CMS-T           GGCCCATCTTTTATATCTATACCGGAAAAGCGCTTCGCTTGATAACGGCATTCATAAATG 1856
                ************************************************************

NUMT_1          AAAGGGAACTTCTACTCAGGCAGGCCCATTGGTTTAGTCAAGCCATCCCGTTGAAAAAA  1914
NUMT_3          AAAGGGAACTTCTACTCAGGCAGGCCCATTGGTTTAGTCAAGCCATCCCGTTGAAAAAA  1914
NA              AAAGGGAACTTCTACTCAGGCAGGCCCATTGGTTTAGTCAAGCCATCCCGTTGAAAAAAA 1915
Zmp             AAAGGGAACTTCTACTCAGGCAGGCCCATTGGTTTAGTCAAGCCATCCCGTTGAAAAAAA 1915
CMS-S           AAAGGGAACTTCTACTCAGGCAGGCCCATTGGTTTAGTCAAGCCATCCCGTTGAAAAAAA 1915
NUMT_2          AAAGGGAACTTCTACTCAGGCAGGCCCATTGGTTTAGTCAAGCCATCCCGTTGAAAAAAA 1915
CMS-T           AAAGGGAACTTCTACTCAGGCAGGCCCATTGGTTTAGTCAAGCCATCCCGTTGAAAAAAA 1916
                ***********************************************************
```

```
NUMT_1          TCCTCTGTTTTCTATCAGAGAAGTCTAACTATTCCACTTGGCTTAAGAGAGATAGATACC 1974
NUMT_3          TCCTCTGTTTTCTATCAGAGAAGTCTAACTATTCCACTTGGCTTAAGAGAGATAGATACC 1974
NA              TCCTCTGTTTTCTATCAGAGAAGTCTAACTATTCCACTTGGCTTAAGAGAGATAGATACC 1975
Zmp             TCCTCTGTTTTCTATCAGAGAAGTCTAACTATTCCACTTGGCTTAAGAGAGATAGATACC 1975
CMS-S           TCCTCTGTTTTCTATCAGAGAAGTCTAACTATTCCACTTGGCTTAAGAGAGATAGATACC 1975
NUMT_2          TCCTCTGTTTTCTATCAGAGAAGTCTAACTATTCCACTTGGCTTAAGAGAGATAGATACC 1975
CMS-T           TCCTCTGTTTTCTATCAGAGAAGTCTAACTATTCCACTTGGCTTAAGAGAGATAGATACC 1976
                ************************************************************

NUMT_1          GATACGCCCTCTTTTGCGCCAACAAGTCCCCGTGACGATAGTAGTCTTTATTCAGAAGGG 2034
NUMT_3          GATACGCCCTCTTTTGCGCCAACAAGTCCCCGTGACGATAGTAGTCTTTATTCAGAAGGG 2034
NA              GATACGCCCTCTTTTGCGCCAACAAGTCCCCGTGACGATAGTAGTCTTTATTCAGAAGGG 2035
Zmp             GATACGCCCTCTTTTGCGCCAACAAGTCCCCGTGACGATAGTAGTCTTTATTCAGAAGGG 2035
CMS-S           GATACGCCCTCTTTTGCGCCAACAAGTCCCCGTGACGATAGTAGTCTTTATTCAGAAGGG 2035
NUMT_2          GATACGCCCTCTTTTGCGCCAACAAGTCCCCGTGACGATAGTAGTCTTTATTCAGAAGGG 2035
CMS-T           GATACGCCCTCTTTTGCGCCAACAAGTCCCCGTGACGATAGTAGTCTTTATTCAGAAGGG 2036
                ************************************************************

NUMT_1          CAGGGTGAGATAGGTAGCCACACCTATGTATGATCAAGCTAAAGTATACATCATGGCTGT 2094
NUMT_3          CAGGGTGAGATAGGTAGCCACACCTATGTATGATCAAGCTAAAGTATACATCATGGCTGT 2094
NA              CAGGGTGAGATAGGTAGCCACACCTATGTATGATCAAGCTAAAGTATACATCATGGCTGT 2095
Zmp             CAGGGTGAGATAGGTAGCCACACCTATGTATGATCAAGCTAAAGTATACATCATGGCTGT 2095
CMS-S           CAGGGTGAGATAGGTAGCCACACCTATGTATGATCAAGCTAAAGTATACATCATGGCTGT 2095
NUMT_2          CAGGGTGAGATAGGTAGCCACACCTATGTATGATCAAGCTAAAGTATACATCATGGCTGT 2095
CMS-T           CAGGGTGAGATAGGTAGCCACACCTATGTATGATCAAGCTAAAGTATACATCATGGCTGT 2096
                ************************************************************

NUMT_1          ATCTGCCAGAGCGCCCTTCTTATGCCAATTAGCAGGTTTGTTACAAATCATGGGAACAAA 2154
NUMT_3          ATCTGCCAGAGCGCCCTTCTTATGCCAATTAGCAGGTTTGTTACAAATCATGGGAACAAA 2154
NA              ATCTGCCAGAGCGCCCTTCTTATGCCAATTAGCAGGTTTGTTACAAATCATGGGAACAAA 2155
Zmp             ATCTGCCAGAGCGCCCTTCTTATGCCAATTAGCAGGTTTGTTACAAATCATGGGAACAAA 2155
CMS-S           ATCTGCCAGAGCGCCCTTCTTATGCCAATTAGCAGGTTTGTTACAAATCATGGGAACAAA 2155
NUMT_2          ATCTGCCAGAGCGCCCTTCTTATGCCAATTAGCAGGTTTGTTACAAATCATGGGAACAAA 2155
CMS-T           ATCTGCCAGAGCGCCCTTCTTATGCCAATTAGCAGGTTTGTTACAAATCATGGGAACAAA 2156
                ************************************************************

NUMT_1          TATAAAATACAAGACTCTGATCTACTCAAAGGTCAGATGGGTTGGTTGAATTTCTTCATA 2214
NUMT_3          TATAAAATACAAGACTCTGATCTACTCAAAGGTCAGATGGGTTGGTTGAATTTCTTCATA 2214
NA              TATAAAATACAAGACTCTGATCTACTCAAAGGTCAGATGGGTTGGTTGAATTTCTTCATA 2215
Zmp             TATAAAATACAAGACTCTGATCTACTCAAAGGTCAGATGGGTTGGTTGAATTTCTTCATA 2215
CMS-S           TATAAAATACAAGACTCTGATCTACTCAAAGGTCAGATGGGTTGGTTGAATTTCTTCATA 2215
NUMT_2          TATAAAATACAAGACTCTGATCTACTCAAAGGTCAGATGGGTTGGTTGAATTTCTTCATA 2215
CMS-T           TATAAAATACAAGACTCTGATCTACTCAAAGGTCAGATGGGTTGGTTGAATTTCTTCATA 2216
                ************************************************************

NUMT_1          AAGAGTAGGCTTCTATGCCGCTATCTATGCCACAAGGCTATCCGAAGCGAGCCATAAGAG 2274
NUMT_3          AAGAGTAGGCTTCTATGCCGCTATCTATGCCACAAGGCTATCCGAAGCGAGCCATAAGAG 2274
NA              AAGAGTAGGCTTCTATGCCGCTATCTATGCCACAAGGCTATCCGAAGCGAGCCATAAGAG 2275
Zmp             AAGAGTAGGCTTCTATGCCGCTATCTATGCCACAAGGCTATCCGAAGCGAGCCATAAGAG 2275
CMS-S           AAGAGTAGGCTTCTATGCCGCTATCTATGCCACAAGGCTATCCGAAGCGAGCCATAAGAG 2275
NUMT_2          AAGAGTAGGCTTCTATGCCGCTATCTATGCCACAAGGCTATCCGAAGCGAGCCATAAGAG 2275
CMS-T           AAGAGTAGGCTTCTATGCCGCTATCTATGCCACAAGGCTATCCGAAGCGAGCCATAAGAG 2276
                ************************************************************

NUMT_1          AGCCTTGTCCTAGTATTAGGAGCGATGGAGCTTTTCCAGTGAAAGGAATAC TAGCGAGT 2334
NUMT_3          AGCCTTGTCCTAGTATTAGGAGCGATGGAGCTTTTCCAGTGAAAGGAATAC TAGCGAGT 2334
NA              AGCCTTGTCCTAGTATTAGGAGCGATGGAGCTTTTCCAGTGAAAGGAATACGTAGCGAGT 2335
Zmp             AGCCTTGTCCTAGTATTAGGAGCGATGGAGCTTTTCCAGTGAAAGGAATACGTAGCGAGT 2335
CMS-S           AGCCTTGTCCTAGTATTAGGAGCGATGGAGCTTTTCCAGTGAAAGGAATACGTAGCGAGT 2335
NUMT_2          AGCCTTGTCCTAGTATTAGGAGCGATGGAGCTTTTCCAGTGAAAGGAATACGTAGCGAGT 2335
CMS-T           AGCCTTGTCCTAGTATTAGGAGCGATGGAGCTTTTCCAGTGAAAGGAATACGTAGCGAGT 2336
                *************************************************** ********

NUMT_1          CACGGGAATAATAGAAAAGCACTCTTCGGGGGCTCACTCTCGCCTCTATTACATAACCTT 2394
NUMT_3          CACGGGAATAATAGAAAAGCACTCTTCGGGGGCTCACTCTCGCCTCTATTACATAACCTT 2394
NA              CACGGGAATAATAGAAAAGCACTCTTCGGGGGCTCACTCTCGCCTCTATTACATAACCTT 2395
Zmp             CACGGGAATAATAGAAAAGCACTCTTCGGGGGCTCACTCTCGCCTCTATTACATAACCTT 2395
CMS-S           CACGGGAATAATAGAAAAGCACTCTTCGGGGGCTCACTCTCGCCTCTATTACATAACCTT 2395
NUMT_2          CACGGGAATAATAGAAAAGCACTCTTCGGGGGCTCACTCTCGCCTCTATTACATAACCTT 2395
CMS-T           CACGGGAATAATAGAAAAGCACTCTTCGGGGGCTCACTCTCGCCTCTATTACATAACCTT 2396
                ************************************************************
```

```
NUMT_1          TCCCCGGTATACTCCCCCTTCGAGATGGTCACTCAACCTATTGAAGAGCCTGGCATGGAA 2454
NUMT_3          TCCCCGGTATACTCCCCCTTCGAGATGGTCACTCAACCTATTGAAGAGCCTGGCATGGAA 2454
NA              TCCCCGGTATACTCCCCCTTCGAGATGGTCACTCAACCTATTGAAGAGCCTGGCATGGAA 2455
Zmp             TCCCCGGTATACTCCCCCTTCGAGATGGTCACTCAACCTATTGAAGAGCCTGGCATGGAA 2455
CMS-S           TCCCCGGTATACTCCCCCTTCGAGATGGTCACTCAACCTATTGAAGAGCCTGGCATGGAA 2455
NUMT_2          TCCCCGGTATACTCCCCCTTCGAGATGGTCACTCAACCTATTGAAGAGCCTGGCATGGAA 2455
CMS-T           TCCCCGGTATACTCCCCCTTCGAGATGGTCACTCAACCTATTGAAGAGCCTGGCATGGAA 2456
                ************************************************************

NUMT_1          GACCATCTTTAAGAGAGAAAGTTCCTTGCCAAGAGCAATCCAAGGCCCTCTCAACCCAAG 2514
NUMT_3          GACCATCTTTAAGAGAGAAAGTTCCTTGCCAAGAGCAATCCAAGGCCCTCTCAACCCAAG 2514
NA              GACCATCTTTAAGAGAGAAAGTTCCTTGCCAAGAGCAATCCAAGGCCCTCTCAACCCAAG 2515
Zmp             GACCATCTTTAAGAGAGAAAGTTCCTTGCCAAGAGCAATCCAAGGCCCTCTCAACCCAAG 2515
CMS-S           GACCATCTTTAAGAGAGAAAGTTCCTTGCCAAGAGCAATCCAAGGCCCTCTCAACCCAAG 2515
NUMT_2          GACCATCTTTAAGAGAGAAAGTTCCTTGCCAAGAGCAATCCAAGGCCCTCTCAACCCAAG 2515
CMS-T           GACCATCTTTAAGAGAGAAAGTTCCTTGCCAAGAGCAATCCAAGGCCCTCTCAACCCAAG 2516
                ************************************************************

NUMT_1          ACAAGAGGTCTTGAGTCCATATGGTTACCACCTAAAGCGGATACCGTAAAATCGAGGTAG 2574
NUMT_3          ACAAGAGGTCTTGAGTCCATATGGTTACCACCTAAAGCGGATACCGTAAAATCGAGGTAG 2574
NA              ACAAGAGGTCTTGAGTCCATATGGTTACCACCTAAAGCGGATACCGTAAAATCGAGGTAG 2575
Zmp             ACAAGAGGTCTTGAGTCCATATGGTTACCACCTAAAGCGGATACCGTAAAATCGAGGTAG 2575
CMS-S           ACAAGAGGTCTTGAGTCCATATGGTTACCACCTAAAGCGGATACCGTAAAATCGAGGTAG 2575
NUMT_2          ACAAGAGGTCTTGAGTCCATATGGTTACCACCTAAAGCGGATACCGTAAAATCGAGGTAG 2575
CMS-T           ACAAGAGGTCTTGAGTCCATATGGTTACCACCTAAAGCGGATACCGTAAAATCGAGGTAG 2576
                ************************************************************

NUMT_1          AAATCTCTAGACCTTCCCCCCAGGCGTATAAACGGTTATTGAAAAAACGGAACGATGTTG 2634
NUMT_3          AAATCTCTAGACCTTCCCCCCAGGCGTATAAACGGTTATTGAAAAAACGGAACGATGTTG 2634
NA              AAATCTCTAGACCTTCCCCCCAGGCGTATAAACGGTTATTGAAAAAACGGAACGATGTTG 2635
Zmp             AAATCTCTAGACCTTCCCCCCAGGCGTATAAACGGTTATTGAAAAAACGGAACGATGTTG 2635
CMS-S           AAATCTCTAGACCTTCCCCCCAGGCGTATAAACGGTTATTGAAAAAACGGAACGATGTTG 2635
NUMT_2          AAATCTCTAGACCTTCCCCCCAGGCGTATAAACGGTTATTGAAAAAACGGAACGATGTTG 2635
CMS-T           AAATCTCTAGACCTTCCCCCCAGGCGTATAAACGGTTATTGAAAAAACGGAACGATGTTG 2636
                ************************************************************

NUMT_1          CTTTGCATGGGAAGCTGCCACCTGTCTGTAATAGGAACCGAGTCGCTAAAGGAGTTTCAT 2694
NUMT_3          CTTTGCATGGGAAGCTGCCACCTGTCTGTAATAGGAACCGAGTCGCTAAAGGAGTTTCAT 2694
NA              CTTTGCATGGGAAGCTGCCACCTGTCTGTAATAGGAACCGAGTCGCTAAAGGAGTTTCAT 2695
Zmp             CTTTGCATGGGAAGCTGCCACCTGTCTGTAATAGGAACCGAGTCGCTAAAGGAGTTTCAT 2695
CMS-S           CTTTGCATGGGAAGCTGCCACCTGTCTGTAATAGGAACCGAGTCGCTAAAGGAGTTTCAT 2695
NUMT_2          CTTTGCATGGGAAGCTGCCACCTGTCTGTAATAGGAACCGAGTCGCTAAAGGAGTTTCAT 2695
CMS-T           CTTTGCATGGGAAGCTGCCACCTGTCTGTAATAGGAACCGAGTCGCTAAAGGAGTTTCAT 2696
                ************************************************************

NUMT_1          CAGCATCGAGTTATTGAGCCCACGGAACGGGGAGTGTTAACGAGTCACTAACCCGTAGTG 2754
NUMT_3          CAGCATCGAGTTATTGAGCCCACGGAACGGGGAGTGTTAACGAGTCACTAACCCGTAGTG 2754
NA              CAGCATCGAGTTATTGAGCCCACGGAACGGGGAGTGTTAACGAGTCACTAACCCGTAGTG 2755
Zmp             CAGCATCGAGTTATTGAGCCCACGGAACGGGGAGTGTTAACGAGTCACTAACCCGTAGTG 2755
CMS-S           CAGCATCGAGTTATTGAGCCCACGGAACGGGGAGTGTTAACGAGTCACTAACCCGTAGTG 2755
NUMT_2          CAGCATCGAGTTATTGAGCCCACGGAACGGGGAGTGTTAACGAGTCACTAACCCGTAGTG 2755
CMS-T           CAGCATCGAGTTATTGAGCCCACGGAACGGGGAGTGTTAACGAGTCACTAACCCGTAGTG 2756
                ************************************************************

NUMT_1          CTTCCTAAACCTCGCGATTCGACCGTTGGAGCTGAGTCAATCTTGCCAAATTGGCAAATG 2814
NUMT_3          CTTCCTAAACCTCGCGATTCGACCGTTGGAGCTGAGTCAATCTTGCCAAATTGGCAAATG 2814
NA              CTTCCTAAACCTCGCGATTCGACCGTTGGAGCTGAGTCAATCTTGCCAAATTGGCAAATG 2815
Zmp             CTTCCTAAACCTCGCGATTCGACCGTTGGAGCTGAGTCAATCTTGCCAAATTGGCAAATG 2815
CMS-S           CTTCCTAAACCTCGCGATTCGACCGTTGGAGCTGAGTCAATCTTGCCAAATTGGCAAATG 2815
NUMT_2          CTTCCTAAACCTCGCGATTCGACCGTTGGAGCTGAGTCAATCTTGCCAAATTGGCAAATG 2815
CMS-T           CTTCCTAAACCTCGCGATTCGACCGTTGGAGCTGAGTCAATCTTGCCAAATTGGCAAATG 2816
                ************************************************************

NUMT_1          TCTGGGTCTGTCCTATAGAATAGCTCATGGAACTTGCTTTCCAGATCGTACCAACTGTTA 2874
NUMT_3          TCTGGGTCTGTCCTATAGAATAGCTCATGGAACTTGCTTTCCAGATCGTACCAACTGTTA 2874
NA              TCTGGGTCTGTCCTATAGAATAGCTCATGGAACTTGCTTTCCAGATCGTACCAACTGTTA 2875
Zmp             TCTGGGTCTGTCCTATAGAATAGCTCATGGAACTTGCTTTCCAGATCGTACCAACTGTTA 2875
CMS-S           TCTGGGTCTGTCCTATAGAATAGCTCATGGAACTTGCTTTCCAGATCGTACCAACTGTTA 2875
NUMT_2          TCTGGGTCTGTCCTATAGAATAGCTCATGGAACTTGCTTTCCAGATCGTACCAACTGTTA 2875
CMS-T           TCTGGGTCTGTCCTATAGAATAGCTCATGGAACTTGCTTTCCAGATCGTACCAACTGTTA 2876
                ************************************************************
```

```
NUMT_1          ATAGAGTTTGGAGAGATGCTCATATACCAGGGGAAGGCCGATTTAGTAAGAGAGTTGGCA 2934
NUMT_3          ATAGAGTTTGGAGAGATGCTCATATACCAGGGGAAGGCCGATTTAGTAAGAGAGTTGGCA 2934
NA              ATAGAGTTTGGAGAGATGCTCATATACCAGGGGAAGGCCGATTTAGTAAGAGAGTTGGCA 2935
Zmp             ATAGAGTTTGGAGAGATGCTCATATACCAGGGGAAGGCCGATTTAGTAAGAGAGTTGGCA 2935
CMS-S           ATAGAGTTTGGAGAGATGCTCATATACCAGGGGAAGGCCGATTTAGTAAGAGAGTTGGCA 2935
NUMT_2          ATAGAGTTTGGAGAGATGCTCATATACCAGGGGAAGGCCGATTTAGTAAGAGAGTTGGCA 2935
CMS-T           ATAGAGTTTGGAGAGATGCTCATATACCAGGGGAAGGCCGATTTAGTAAGAGAGTTGGCA 2936
                ************************************************************

NUMT_1          AAGAGCCAAAGCTTGAGGCAGTCCTTGGACCTAGCTTCCCCGCACTGGACCGAGAACCGA 2994
NUMT_3          AAGAGCCAAAGCTTGAGGCAGTCCTTGGACCTAGCTTCCCCGCACTGGACCGAGAACCGA 2994
NA              AAGAGCCAAAGCTTGAGGCAGTCCTTGGACCTAGCTTCCCCGCACTGGACCGAGAACCGA 2995
Zmp             AAGAGCCAAAGCTTGAGGCAGTCCTTGGACCTAGCTTCCCCGCACTGGACCGAGAACCGA 2995
CMS-S           AAGAGCCAAAGCTTGAGGCAGTCCTTGGACCTAGCTTCCCCGCACTGGACCGAGAACCGA 2995
NUMT_2          AAGAGCCAAAGCTTGAGGCAGTCCTTGGACCTAGCTTCCCCGCACTGGACCGAGAACCGA 2995
CMS-T           AAGAGCCAAAGCTTGAGGCAGTCCTTGGACCTAGCTTCCCCGCACTGGACCGAGAACCGA 2996
                ************************************************************

NUMT_1          GCTATATGCTCAATAGTCGATCGACTTATTCTCCTCACCGGAAAAGAGAACAAACTCTGG 3054
NUMT_3          GCTATATGCTCAATAGTCGATCGACTTATTCTCCTCACCGGAAAAGAGAACAAACTCTGG 3054
NA              GCTATATGCTCAATAGTCGATCGACTTATTCTCCTCACCGGAAAAGAGAACAAACTCTGG 3055
Zmp             GCTATATGCTCAATAGTCGATCGACTTATTCTCCTCACCGGAAAAGAGAACAAACTCTGG 3055
CMS-S           GCTATATGCTCAATAGTCGATCGACTTATTCTCCTCACCGGAAAAGAGAACAAACTCTGG 3055
NUMT_2          GCTATATGCTCAATAGTCGATCGACTTATTCTCCTCACCGGAAAAGAGAACAAACTCTGG 3055
CMS-T           GCTATATGCTCAATAGTCGATCGACTTATTCTCCTCACCGGAAAAGAGAACAAACTCTGG 3056
                ************************************************************

NUMT_1          CACCTTGGAAACTGAGGAACTCGGTCGATCCAATCAGGGTATGCTTTCCTAGCCGTCGGC 3114
NUMT_3          CACCTTGGAAACTGAGGAACTCGGTCGATCCAATCAGGGTATGCTTTCCTAGCCGTCGGC 3114
NA              CACCTTGGAAACTGAGGAACTCGGTCGATCCAATCAGGGTATGCTTTCCTAGCCGTCGGC 3115
Zmp             CACCTTGGAAACTGAGGAACTCGGTCGATCCAATCAGGGTATGCTTTCCTAGCCGTCGGC 3115
CMS-S           CACCTTGGAAACTGAGGAACTCGGTCGATCCAATCAGGGTATGCTTTCCTAGCCGTCGGC 3115
NUMT_2          CACCTTGGAAACTGAGGAACTCGGTCGATCCAATCAGGGTATGCTTTCCTAGCCGTCGGC 3115
CMS-T           CACCTTGGAAACTGAGGAACTCGGTCGATCCAATCAGGGTATGCTTTCCTAGCCGTCGGC 3116
                ************************************************************

NUMT_1          CTCAGATGGACGAGACAAAGAACGCAACTCAGGGGGCGAAGGTGGAGGTCCGGGCAAGGC 3174
NUMT_3          CTCAGATGGACGAGACAAAGAACGCAACTCAGGGGGCGAAGGTGGAGGTCCGGGCAAGGC 3174
NA              CTCAGATGGACGAGACAAAGAACGCAACTCAGGGGGCGAAGGTGGAGGTCCGGGCAAGGC 3175
Zmp             CTCAGATGGACGAGACAAAGAACGCAACTCAGGGGGCGAAGGTGGAGGTCCGGGCAAGGC 3175
CMS-S           CTCAGATGGACGAGACAAAGAACGCAACTCAGGGGGCGAAGGTGGAGGTCCGGGCAAGGC 3175
NUMT_2          CTCAGATGGACGAGACAAAGAACGCAACTCAGGGGGCGAAGGTGGAGGTCCGGGCAAGGC 3175
CMS-T           CTCAGATGGACGAGACAAAGAACGCAACTCAGGGGGCGAAGGTGGAGGTCCGGGCAAGGC 3176
                ************************************************************

NUMT_1          TAGAGCCAAGCAGAGGGATGGAGTAGGGGACGGTTGGTGAAAGTAGGAACGAGCGGAATA 3234
NUMT_3          TAGAGCCAAGCAGAGGGATGGAGTAGGGGACGGTTGGTGAAAGTAGGAACGAGCGGAATA 3234
NA              TAGAGCCAAGCAGAGGGATGGAGTAGGGGACGGTTGGTGAAAGTAGGAACGAGCGGAATA 3235
Zmp             TAGAGCCAAGCAGAGGGATGGAGTAGGGGACGGTTGGTGAAAGTAGGAACGAGCGGAATA 3235
CMS-S           TAGAGCCAAGCAGAGGGATGGAGTAGGGGACGGTTGGTGAAAGTAGGAACGAGCGGAATA 3235
NUMT_2          TAGAGCCAAGCAGAGGGATGGAGTAGGGGACGGTTGGTGAAAGTAGGAACGAGCGGAATA 3235
CMS-T           TAGAGCCAAGCAGAGGGATGGAGTAGGGGACGGTTGGTGAAAGTAGGAACGAGCGGAATA 3236
                ************************************************************

NUMT_1          AACGAACGGAATACTCACTCTGTTCTACTCCCCCAGTAGAGCTATATGTAACGAGTCAGA 3294
NUMT_3          AACGAACGGAATACTCACTCTGTTCTACTCCCCCAGTAGAGCTATATGTAACGAGTCAGA 3294
NA              AACGAACGGAATACTCACTCTGTTCTACTCCCCCAGTAGAGCTATATGTAACGAGTCAGA 3295
Zmp             AACGAACGGAATACTCACTCTGTTCTACTCCCCCAGTAGAGCTATATGTAACGAGTCAGA 3295
CMS-S           AACGAACGGAATACTCACTCTGTTCTACTCCCCCAGTAGAGCTATATGTAACGAGTCAGA 3295
NUMT_2          AACGAACGGAATACTCACTCTGTTCTACTCCCCCAGTAGAGCTATATGTAACGAGTCAGA 3295
CMS-T           AACGAACGGAATACTCACTCTGTTCTACTCCCCCAGTAGAGCTATATGTAACGAGTCAGA 3296
                ************************************************************

NUMT_1          CAGACTTTTTACTTGTAGCAAGCGATAAAACGGCTGCACAGTTCTACTGTGCCGAGA 3351
NUMT_3          CAGACTTTTTACTTGTAGCAAGCGATAAAACGGCTGCACAGTTCTACTGTGCCGAGA 3351
NA              CAGACTTTTTACTTGTAGCAAGCGATAAAACGGCTGCACAGTTCTACTGTGCCGAGA 3352
Zmp             CAGACTTTTTACTTGTAGCAAGCGATAAAACGGCTGCACAGTTCTACTGTGCCGAGA 3352
CMS-S           CAGACTTTTTACTTGTAGCAAGCGATAAAACGGCTGCACAGTTCTACTGTGCCGAGA 3352
NUMT_2          CAGACTTTTTACTTGTAGCAAGCGATAAAACGGCTGCACAGTTCTACTGTGCCGAGA 3352
CMS-T           CAGACTTTTTACTTGTAGCAAGCGATAAAACGGCTGCACAGTTCTACTGTGCCGAGA 3353
                *********************************************************
```

Figure S6 Multiple sequence alignment of the three B73 3.3-kb NUMT regions with the corresponding NA, Zmp, CMS-S, and CMS-T mitochondrial genome regions. The 3.3-kb sequence is present within the NA (NCBI Accession DQ490952.1), Zmp (NCBI Accession DQ645539.1), and CMS-S (NCBI Accession DQ490951.2), and CMS-T (NCBI Accession DQ490953.1) mitochondrial genomes. The second copy of the 3.3-kb region within the B73 NUMT has fewer nucleotide differences compared to the NA, Zmp, and CMS-S mitochondrial genomes than the first and third 3.3-kb regions in the NUMT. The sequences were aligned with ClustalW2 (McWilliam *et al.* 2013). The reverse complement of the third 3.3-kb region in the NUMT and the NA genome were used in this alignment.
