# Supplementary material for: Cytogenetic and Sequence Analyses of Mitochondrial DNA Insertions in Nuclear Chromosomes of Maize
Source: G3 (Bethesda). 2015 Sep 1;5(11):2229–39. doi: 10.1534/g3.115.020677 (PMC4632043; doi:10.1534/g3.115.020677)
Supplement: Supporting Information [file supp_g3.115.020677_FigureS7.pdf]

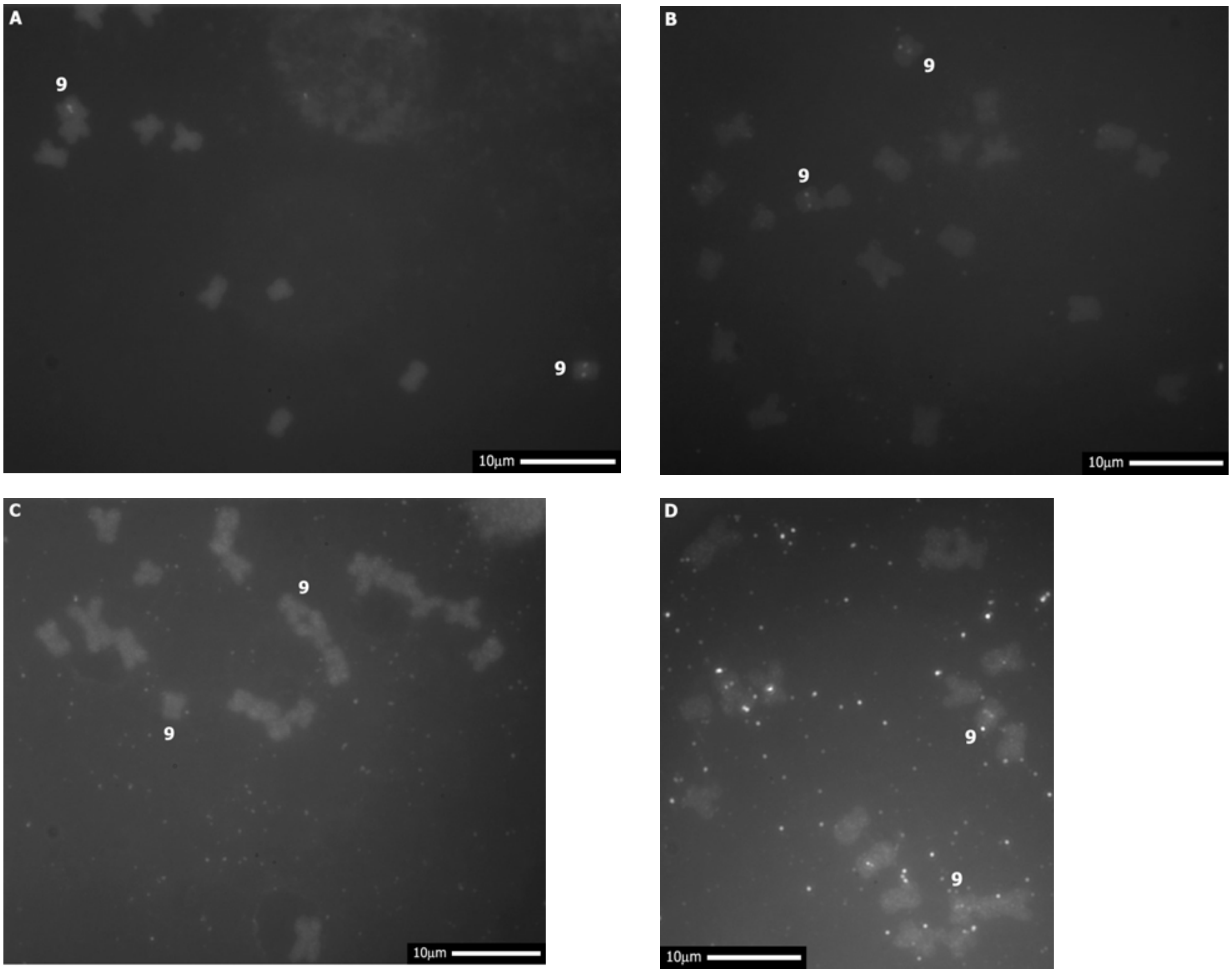

Figure S7 The 2.4- and 3.3-kb probes hybridized to the 9L NUMT in the B73 line. Only small portions of 2.4- and 3.3-kb DNA segments (≤ 82 bp) are found within the NB mitochondrial genome (Table 1); therefore, there is minimal background hybridization to the cytoplasmic mtDNA in the B73 line. Shown here are metaphase chromosome spreads hybridized to the indicated probes. (A) The Texas red-labeled 3.3-kb probe (white) hybridized to B73 chromosomes, including the 9L NUMT. Minimal background hybridization is detectable in B73. (B) The Texas red-labeled 2.4-kb probe (white) hybridized to B73 chromosomes, including the 9L NUMT. (C) In contrast, the Texas red-labeled 3.3-kb probe (white) did not hybridize to 9L in Ky21. The Ky21 line contains the NA mitochondrial genome, which includes the 3.3-kb segment. Therefore, the 3.3-kb probe hybridizes to mtDNA from ruptured mitochondria, creating hybridization signals in the cytoplasm. (D) Similar hybridization to cytoplasmic mtDNA can be observed with B73 when using a probe that is present in the NB mitochondrial genome. The Texas red-labeled

cosmid 2 probe (white) hybridized to both B73 chromosomal NUMTs and cytoplasmic mtDNA. Cosmid 2 contains ~31.7 kb of NB mtDNA. The karyotyping probes used to identify each chromosome are not shown. Scale = 10 μm.
