# Supplementary material for: Cytogenetic and Sequence Analyses of Mitochondrial DNA Insertions in Nuclear Chromosomes of Maize
Source: G3 (Bethesda). 2015 Sep 1;5(11):2229–39. doi: 10.1534/g3.115.020677 (PMC4632043; doi:10.1534/g3.115.020677)
Supplement: Supporting Information [file supp_g3.115.020677_FigureS8.pdf]

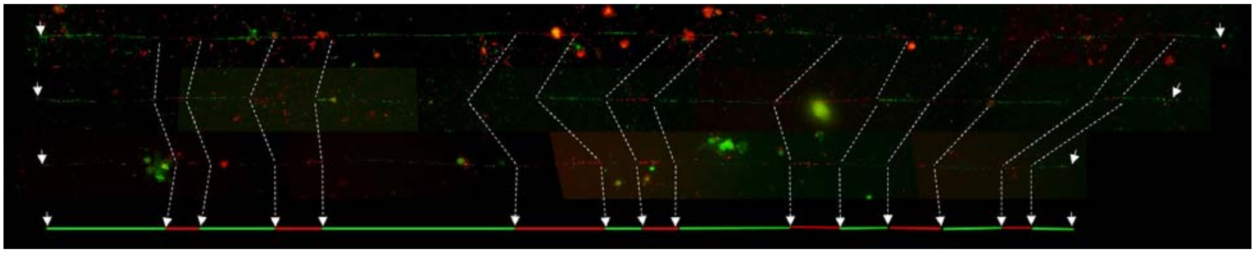

Figure S8 Measurement of the B73 9L NUMT using fiber-FISH. Five intact DNA fibers were measured; the three fibers compared in this figure were hybridized with two mtDNA labels: cosmids 1-7 (red), and cosmids 8-10, 16-18, and 20 (green). The solid red and green line at the bottom of the figure show the composite NUMT. The upper three pairs of arrows on either side of the figure indicate the start and end of the NUMT on the three separate fibers. The red and green colored sections in each fiber are different lengths due to different levels of stretching in the fibers. The alternating green and red labeling pattern among each fiber shows an identical pattern, indicating that each fiber is intact. Dashed lines and arrows that connect the DNA fibers to the solid line at the bottom designate the red and green labels hybridized to the fibers. Sizing was determined similarly to Figure 7. The size of the B73 9L NUMT is estimated to be ~1.8 Mb (1811.6 ± 229.3 kb, n = 5).
