# Supplementary material for: Cytogenetic and Sequence Analyses of Mitochondrial DNA Insertions in Nuclear Chromosomes of Maize
Source: G3 (Bethesda). 2015 Sep 1;5(11):2229–39. doi: 10.1534/g3.115.020677 (PMC4632043; doi:10.1534/g3.115.020677)
Supplement: Supporting Information [file supp_g3.115.020677_FileS1.pdf]

FILE S1

Supporting Materials and Methods

Diverse Maize Lines Examined: For this study, lines were chosen based on their designation in an association panel of 301 diverse maize inbred lines. This panel of lines was genotyped, and the underlying population substructure was established using 89 simple sequence repeat (SSR) loci (Flint-Garcia *et al*. 2005). Three main subgroups were identified: stiff stalk, non-stiff stalk, and tropical/subtropical. Because of their isolated breeding histories, sweet corn and popcorn lines formed their own distinct subgroups, and lines with mixed ancestry were classified as "mixed." From this large panel of inbred lines, 26 inbreds were chosen as founder lines to represent the diversity of maize in a population called the Nested Association Mapping (NAM) population (Yu *et al*. 2008). Each of the 16 lines examined in Figure 1 are NAM founder lines, with the exception of B37, Mo17, and M825.

M825 Pedigree Examined: Sweet corn distinguishes itself based on the presence of one or multiple recessive mutations that affect endosperm starch synthesis (Tracy 2001). The most popular mutations are *sugary1* (*su1*) and *shrunken2* (*sh2*), which both cause an increase of sugar in the endosperm and a decrease in starch content (Neuffer *et al*. 1997; Hannah 2005).

The earliest member of the M825 pedigree (Figure 4) included in this study is the sweet corn line P39. P39 carries the *su1* mutation (McMullen *et al*. 2009) but not *sh2* (Revilla *et al*. 2006; McMullen *et al*. 2009). According to the Maize Genetics and Genomics Database (MaizeGDB; Schaeffer *et al*. 2011), IP39 was produced from a P39 population by selecting for narrow kernels (Schaeffer *et al*. 2011). Ia5125 (also known as I5125 or 5125) was produced by crossing Tendermost onto IP39, then backcrossing by IP39 (Schaeffer *et al*. 2011). Ia5125 also carries *su1* (Ordás *et al*. 2006). Tendermost is a commercial sweet corn line (Wehner 2010) that is not currently available through the Germplasm Resources Information Network (GRIN; USDA 2011). The R825 line was produced from a cross of Ia5125 (*su1*/*su1 Sh2*/*Sh2*) by a *Su1*/*Su1 sh2*/*sh2* stock (stock from Mains 1949), and then repeatedly backcrossing the progeny to Ia5125 (S. Gabay-Laughnan, personal communication; Gerdes *et al*. 1993). R825 was later crossed by the line "Elite" (*sh2*/*sh2 Su1*/*Su1*) and then recurrently self-pollinated to form the line M825

sh2/*sh2 Su1*/*Su1* (S. Gabay-Laughnan, personal communication). Elite is an unavailable sweet corn line most likely made by crossing Golden Cross Bantam (*su1*/*su1*) by the *Su1*/*Su1 sh2*/*sh2* stock (Mains 1949) discussed above (S. Gabay-Laughnan, personal communication). M825 was then crossed onto Wf9 *Sh2*/*Sh2 A1*/*A1* and backcrossed to M825 for 10 generations, recurrently selecting for *Sh2* and the tightly linked *A1* locus (*anthocyaninless1*; S. Gabay-Laughnan, personal communication). Wf9 is a yellow dent line, not a sweet corn line (Gerdes *et al*. 1993; Schaeffer *et al*. 2011). The resulting sweet corn-derived M825 line has been self-pollinated since the backcrossing was completed (S. Gabay-Laughnan, personal communication). The final version of M825 was examined in this study.

Amplification of 2.4 and 3.3 kb Regions: The 2.4 kb region primers were designed based on the BAC AC183911 sequence (Table S2) using the program FastPCR (Kalendar *et al*. 2009). Two sets of 3.3 kb region primers were designed (Table S2) and used to produce PCR products for nick translation probe preparation procedures. The 3.3 kb region primers designed based on the BAC AC187467 were identified using the program FastPCR (Kalendar *et al*. 2009). The 3.3 kb region primers designed based on the BAC AC183911 were identified using the program Primer3 (Rozen and Skaletsky 2000). BAC DNA was isolated for amplification using the QIAGEN Plasmid plus midi kit (catalog number 12943, Valencia, CA).

Forward and reverse primers (Table S2) were used at a final concentration of 0.1 µM each in a PCR reaction with Promega GoTaq Green Master Mix (catalog number M712, Madison WI). Isolated BAC DNA for amplification of the regions was used at a final concentration of ~1 ng in a 100 µl PCR reaction. The PCR reaction mix was made of: 2 µl forward primer (10 µM), 2 µl reverse primer (10 µM), 2 µl isolated BAC DNA, 50 µl Promega GoTaq Green Master Mix, and 44 µl sterile water. The PCR parameters for the 2.4 kb and 3.3 kb regions were: 1) initial denaturation at 95° for 5 minutes; 2) denaturation at 95° for 30 seconds; 3) annealing at 55° for 30 seconds; 4) extension at 72° for 4 minutes; and 5) final extension at 72° for 8 minutes. Steps 2 - 4 were repeated for 40 cycles. The PCR products were purified using the Promega Wizard SV Gel and PCR Cleanup System Kit (catalog number A9282, Madison, WI).

FISH Methodology: The protocols used for preparing slides of root tip chromosome spreads, the labeling of both karyotyping and cosmid probes, and the capturing and processing of FISH images were previously described by Lough *et al*. (2008). Alterations to those methods are detailed below.

FISH Probes: The karyotyping probes used to identify chromosomes included eight regions of repetitive DNA common to most maize lines (Kato *et al*. 2004). These probes were labeled through nick translation with the fluorescence-labeled nucleotides: Cascade Blue-7-dUTP, Alexa Fluor 488-5-dUTP, or Cyanine 5-dUTP (Cy5). Mitochondrial DNA-containing cosmid probes were produced using segments of the NB maize mitochondrial genome and were labeled by nick translation with Texas red-5-dCTP (Lough *et al*. 2008). The NB mitochondrial genome was previously sequenced from the stiff stalk line B37 (Clifton *et al*. 2004) and is also present in B73. These mtDNA probes are either 20 individually labeled segments (Table S1) of the mitochondrial genome or a combination in a 19-cosmid mix probe (Figure 2A). Cosmid 13 is not included in this mix of 19 segments because it contains plastid DNA (Lough *et al*. 2008). The 2.4- and 3.3-kb probes were also labeled with Texas Red-5-dCTP.

Root Tip Mitotic Metaphase Chromosome Spread Slide Preparation: To obtain mitotic metaphase chromosomes for experiments, root tips were digested with cellulase and pectolyase, then rinsed with 1X TE and 100% ethanol (Lough *et al*. 2008). After removing the ethanol, 100% acetic acid was added to the tube, and the root tip was broken. This suspension was deposited on slides, and the slides were UV crosslinked (120-5 mJ/cm2). Formaldehyde was not used in the preparation or hybridization of slides.

Hybridization of FISH Slides: For experiments using the 19-cosmid probe, the mixed probes for each slide included 2.5 µl of the 19-cosmid mix (final concentration 200 ng/µl), 0.8 µl of 2X SSC/1X TE, and 1.7 µl of the mix of 8 karyotyping probes (Lough *et al*. 2008). The concentrations of the eight karyotyping probes varied (according to Table 1 in Lough *et al*. 2008). For experiments using the individual cosmid probes, the mixed probes for each slide included 1 µl of the labeled single cosmid probe (final concentration 20 ng/µl) and 4 µl of 8 karyotyping probe mix (with 2X SSC/1X TE); or 1 µl of the labeled single cosmid probe (final concentration 20 ng/µl), 0.35 µl Cent C probe (final concentration 3.5 ng/µl), 0.5

µl knob probe (final concentration 20 ng/µl), 0.5 µl 4-12-1 probe (final concentration 20 ng/µl), and 2.65 µl 2X SSC/1X TE. The latter mix was used to recognize chromosome 9 specifically. For experiments using the 2.4- and 3.3-kb probes, the mixed probes for each slide included 1 µl 2.4- or 3.3-kb probe (final concentration 40 ng/µl); 0.35 µl Cent C probe (final concentration 3.5 ng/µl); 0.5 µl knob probe (final concentration 20 ng/µl); 0.5 µl 4-12-1 probe (final concentration 20 ng/µl); and 2.65 µl 2X SSC/1X TE. After the hybridization and washing of the slides, the slides were mounted using Vectashield (catalog number NC9524612 through Fisher Scientific, Vector Laboratories, Burlingame, CA) that contained 4',6-diamidino- 2-phenylindole (DAPI).

Pachytene FISH Methodology: Pachytene slides were made from B73 anthers. Tassels were collected and placed in a 3:1 solution of ethanol and acetic acid for 24 - 48 hours while stored at 4°. The tassels were rinsed in 70% ethanol and stored in -20°. Individual anthers were examined for pachytene chromosomes using acetocarmine staining. A single anther was placed on a slide in a drop of acetocarmine and then smashed using a dissecting needle. A coverslip was put over the smashed anther, and the slide was gently heated over a flame. When pachytene chromosomes were identified, the remaining anthers in that area of the tassel were prepared similar to the root tip slide preparations. After an enzyme digestion, the anthers were rinsed once using TE and three times using 100% ethanol. The anthers were broken in a 3:1 solution of acetic acid and methanol. The resulting solution was dropped onto slides and slides were UV crosslinked.

The BAC-specific (BAC-L1) and *glossy15* (*gl15*) FISH probes were made as described in Danilova and Birchler (2008). Both the BAC-specific and *gl15* probe were prepared using the fluorescence-labeled nucleotide Alexa Fluor 488-5-dUTP. Slides were hybridized using the same procedure as root tip slides. Each of the following probes were used per slide: 0.64 µl Alexa Fluor 488-labeled BAC-specific probe (200 ng/µl), 0.37 µl Alexa Fluor 488-labeled *gl15* probe (200 ng/µl), 1.0 µl Texas Red-labeled cosmid 3 probe (100 ng/µl), 1.0 µl Texas Red-labeled cosmid 9 probe (100 ng/µl), and 1.99 µl 2X SSC/1X TE.

Fiber-FISH Methodology: The fiber-FISH procedures used for preparing slides and labels, and hybridizing slides in this study were defined previously by Koo *et al*. (2011). To detect mtDNA insertions on B73 DNA fibers, different combinations of two-color fiber-FISH procedure were used, as described in Jackson *et al*. (1998). The first combination included only labels of mtDNA: digoxigenin labeled probe A (cosmids 1-7) detected as red and biotin labeled probe B (cosmids 8-10, 16-18, and 20) detected as green. The second combination included labels of mtDNA and 5-methylcytosine. The mtDNA was detected using a biotin-16-dUTP (catalog number 11093070910, Roche, Indianapolis, IN) labeled probe for 19 of the 20 available overlapping sections of the 570-kb NB maize mitochondrial genome cloned into cosmid vectors (Lough *et al*. 2008). Cosmid 13 was omitted because it contains a large amount of plastid DNA. After hybridization, the slides were washed, and the mtDNA label was detected with goat anti-biotin antibody (catalog number SP-3000, Vector Laboratories, Burlingame, CA) and then rhodamine-conjugated donkey anti-goat antibody (catalog number 705-296-147, Jackson ImmunoResearch, West Grove, PA) was used to stain (Koo *et al*. 2011). For the detection of 5-methylcytosine, slides were incubated with mouse antiserum that was raised against 5-methylcytosine (catalog number MABE146, Millipore, Massachusetts); these antibodies were subsequently exposed to rabbit anti-mouse Alexa Fluor 488 antibody (catalog number A27023, Invitrogen, Grand Island, NY). When measuring the 9L NUMT DNA fibers, red and green colored sections derived from each fiber showed different lengths, reflecting the different stretching degrees in individual fibers. However, the alternative green and red labeling pattern among individual DNA fibers showed an identical pattern, indicating intact fibers. Five intact fibers were measured: one fiber with mtDNA and methylation labels and four fibers with only mtDNA labels (Figure S8). The cytological measurements of the fiber-FISH signals were converted into kilobases using a 3 kb per µm conversion rate (Cheng *et al.* 2002).

Sequence Analysis of the B73 Chromosome 9L NUMT: The maize NB mitochondrial genome was compared to the maize nuclear reference genome assembly version 2 using the ZeAlign program available through MaizeGDB (Sen *et al*. 2010). The NB mitochondrial genome was used for these comparisons because B73 contains this mitochondrial genome and because the FISH experiments used probes for this genome. The resulting files from this comparison were uploaded as a custom track on the

maize genome browser at MaizeGDB to visualize the mtDNA insertions relative to the chromosome 9 centromere, BACs, and the gene models (Sen *et al*. 2010). The gene models displayed on the MaizeGDB genome browser were identified by the Maize Sequencing Consortium release 5b.60 (Sen *et al*. 2010). Retrotransposons were detected using the program RepeatMasker (Smit *et al*. 1996-2010). The annotated gene models were given a functional identification based on the presence of a known mitochondrial gene in that location (NCBI Accession AY506529.1; Clifton *et al*. 2004) and InterProScan results, which identify protein domains (Zdobnov and Apweiler 2001).
