# Supplementary material for: Cytogenetic and Sequence Analyses of Mitochondrial DNA Insertions in Nuclear Chromosomes of Maize
Source: G3 (Bethesda). 2015 Sep 1;5(11):2229–39. doi: 10.1534/g3.115.020677 (PMC4632043; doi:10.1534/g3.115.020677)
Supplement: Supporting Information [file supp_g3.115.020677_TableS1.pdf]

Table S1 Segments of the NB mitochondrial genome within cosmids.

Cosmid	NB Mitochondrial Start Position (bp)	NB Mitochondrial End Position (bp)
1	560671	28913
2	11115	42912
3	40337	75268
4	65967	103378
5	92547	132562
6	128405	164326
7	158622	195116
8	182577	220863
9	205820	244602
10	239824	273611
11	254638	295072
12	283267	319446
13	312072	351606
14	341466	378752
15	377800	416027
16	411027	447039
17	442367	473713
18	458736	487130
19	487000	520351
20	522351	5100

The base-pair positions of the NB mtDNA represented within each cosmid were estimated from the restriction endonuclease maps (Fauron *et al.* 1987; Fauron and Havlik 1988) compared to the NB mitochondrial genome sequence (NCBI Accession AY506529.1; Clifton *et al*. 2004) using SeqBuilder™ (DNASTAR, Madison, WI).
