# Supplementary material for: Cytogenetic and Sequence Analyses of Mitochondrial DNA Insertions in Nuclear Chromosomes of Maize
Source: G3 (Bethesda). 2015 Sep 1;5(11):2229–39. doi: 10.1534/g3.115.020677 (PMC4632043; doi:10.1534/g3.115.020677)
Supplement: Supporting Information [file supp_g3.115.020677_TableS2.pdf]

Table S2 Primers used to amplify the 2.4 and 3.3 kb regions from BAC DNA.

Region	BAC	Product Size (bp)	Primer Name	Tm (°C)	Primer Sequence
2.4 kb	AC183911	2639	4F1_331-354	59.2	GCTGAAGTAGCCTAAGCGCTTCAA
			6R1_2948-2969	56.7	CTGCCAACTTGTATGCATCACC
3.3 kb	AC187467	3704	6F1_243-266	60.4	ACATCAGCTCAACCTTCTTGCCCA
			2R1_3925-3946	59.3	TGGCTACGAACCACGATGTTGG
3.3 kb	AC183911	3968	AL183911-1F	59.8	ATAGACGGAGGAATGGATACGA
			AL183911-1R	59.7	ACCGTAGAACAAATTCGAGAGG
