# Supplementary material for: Cytogenetic and Sequence Analyses of Mitochondrial DNA Insertions in Nuclear Chromosomes of Maize
Source: G3 (Bethesda). 2015 Sep 1;5(11):2229–39. doi: 10.1534/g3.115.020677 (PMC4632043; doi:10.1534/g3.115.020677)
Supplement: Supporting Information [file supp_g3.115.020677_TableS3.pdf]

Table S3 Locations of retrotransposons in the B73 chromosome 9L NUMT region.

Retrotransposon Sequence[a]	Type	Length (bp)	Nuclear Start Position (bp)	Nuclear End Position (bp)
Gypsy29-ZM_I-int[b]	LTR/Gypsy	254	72725993	72726247
Gypsy-175_ZM-LTR	LTR/Gypsy	721	72736759	72737480
Gypsy-175_ZM-I	LTR/Gypsy	823	72737481	72738304
Gypsy-174_ZM-I	LTR/Gypsy	4963	72738297	72743260
Gypsy-175_ZM-LTR	LTR/Gypsy	584	72743261	72743845
Gypsy-127_ZM-I	LTR/Gypsy	288	72746025	72746313
PREM1A_ZM_LTR	LTR/Copia	224	72746314	72746538
Gypsy-127_ZM-I	LTR/Gypsy	53	72746539	72746592
Gypsy-127_ZM-I	LTR/Gypsy	1850	72747177	72749027
Gypsy-127_ZM-LTR	LTR/Gypsy	252	72749028	72749280
Gypsy-188_ZM-LTR	LTR/Gypsy	669	72768138	72768807
Gypsy-175_ZM-I	LTR/Gypsy	6090	72768747	72774837
Gypsy-188_ZM-LTR	LTR/Gypsy	669	72774838	72775507
Gypsy-109_ZM-I[b]	LTR/Gypsy	447	72803659	72804106
LINE1-12_ZM[b]	LINE/L1	98	72811368	72811466
Gypsy-175_ZM-I	LTR/Gypsy	4812	72820412	72825224
Gypsy-188_ZM-I	LTR/Gypsy	4354	72822853	72827207
ZEON2_ZM_LTR	LTR/Gypsy	663	72827208	72827871
LINE1-50_ZM[b]	LINE/L1	101	72838354	72838455
HOPSCOTCH_ZM_I-int[b]	LTR/Copia	1162	72850584	72851746
Copia33-ZM_I-int[b]	LTR/Copia	57	72875960	72876017
Gypsy-100_ZM-I[b]	LTR/Gypsy	108	72889185	72889293
Gypsy-198_ZM-I[b]	LTR/Gypsy	128	72900074	72900202
Gypsy-127_ZM-LTR	LTR/Gypsy	252	72951840	72952092
Gypsy-127_ZM-I	LTR/Gypsy	1850	72952093	72953943
Gypsy-127_ZM-I	LTR/Gypsy	53	72954528	72954581
PREM1A_ZM_LTR	LTR/Copia	224	72954582	72954806
Gypsy-127_ZM-I	LTR/Gypsy	2884	72954807	72957691
Gypsy-174_ZM-I	LTR/Gypsy	933	72957792	72958725
Gypsy-175_ZM-LTR	LTR/Gypsy	721	72958726	72959447
Gypsy-174_ZM-I	LTR/Gypsy	3869	72959448	72963317
Gypsy-174_ZM-I	LTR/Gypsy	1880	72963434	72965314
Gypsy-175_ZM-I	LTR/Gypsy	824	72965307	72966131
Gypsy-175_ZM-I	LTR/Gypsy	253	72966131	72966384
Gypsy-188_ZM-LTR	LTR/Gypsy	669	72967168	72967837
Gypsy-175_ZM-I	LTR/Gypsy	6090	72967777	72973867
Gypsy-188_ZM-LTR	LTR/Gypsy	669	72973868	72974537
ZDE_ZD	LTR/Gypsy	483	72975345	72975828
ZDE_ZD	LTR/Gypsy	56	72975822	72975878
ZDE_ZD	LTR/Gypsy	105	72975931	72976036
Gypsy-127_ZM-LTR	LTR/Gypsy	435	72976070	72976505
PREM1_ZM	LTR/Copia	514	72976506	72977020
Gypsy-127_ZM-LTR	LTR/Gypsy	51	72977021	72977072
Gypsy-127_ZM-I	LTR/Gypsy	487	72977205	72977692
Gypsy-175_ZM-LTR	LTR/Gypsy	584	72979872	72980456
Gypsy29-ZM_I-int[b]	LTR/Gypsy	254	72990963	72991217

[a]The retrotransposons were identified using the program RepeatMasker (Smit *et al*. 1996-2010).
[b]These retrotransposon fragments overlap with sections of NB mtDNA (Table 1).

Retrotransposons present among the mitochondrial sequences within the 9L NUMT (Table 1) include those whose LTRs have 100% identity.
